# Activity-dependent isomerization of Kv4.2 by Pin1 regulates cognitive flexibility

**DOI:** 10.1038/s41467-020-15390-x

**Published:** 2020-03-26

**Authors:** Jia–Hua Hu, Cole Malloy, G. Travis Tabor, Jakob J. Gutzmann, Ying Liu, Daniel Abebe, Rose-Marie Karlsson, Stewart Durell, Heather A. Cameron, Dax A. Hoffman

**Affiliations:** 1 Section on Molecular Neurophysiology and Biophysics, The Eunice Kennedy Shriver National Institute of Child Health and Human Development, Bethesda, MD 20892 USA; 2 Section on Neuroplasticity, National Institute of Mental Health, Bethesda, MD 20892 USA; 3 Laboratory of Cell Biology, National Cancer Institute, Bethesda, MD 20892 USA; 4 Present Address: Medical Scientist Training Program, Washington University School of Medicine in St. Louis, St. Louis, MO 63110 USA

**Keywords:** Cellular neuroscience, Ion channels in the nervous system

## Abstract

Voltage-gated K^+^ channels function in macromolecular complexes with accessory subunits to regulate brain function. Here, we describe a peptidyl-prolyl *cis*-*trans* isomerase NIMA-interacting 1 (Pin1)-dependent mechanism that regulates the association of the A-type K^+^ channel subunit Kv4.2 with its auxiliary subunit dipeptidyl peptidase 6 (DPP6), and thereby modulates neuronal excitability and cognitive flexibility. We show that activity-induced Kv4.2 phosphorylation triggers Pin1 binding to, and isomerization of, Kv4.2 at the pThr^607^-Pro motif, leading to the dissociation of the Kv4.2-DPP6 complex. We generated a novel mouse line harboring a knock-in Thr607 to Ala (Kv4.2TA) mutation that abolished dynamic Pin1 binding to Kv4.2. CA1 pyramidal neurons of the hippocampus from these mice exhibited altered Kv4.2-DPP6 interaction, increased A-type K^+^ current, and reduced neuronal excitability. Behaviorally, Kv4.2TA mice displayed normal initial learning but improved reversal learning in both Morris water maze and lever press paradigms. These findings reveal a Pin1-mediated mechanism regulating reversal learning and provide potential targets for the treatment of neuropsychiatric disorders characterized by cognitive inflexibility.

## Abstract (web summary)

K + channels function in macromolecular complexes with accessory subunits to regulate neuronal function. Here, the authors describe Pin1-mediated regulation of the Kv4.2 complex, which impacts reversal learning in mice, providing potential treatment for disorders characterized by cognitive inflexibility.

## Introduction

Rapidly activating and inactivating somatodendritic voltage-gated K^+^ (Kv) A-type currents regulate action potential (AP) repolarization and repetitive firing and prevent backpropagation into the dendrites of hippocampal pyramidal neurons^1,2^. Kv4.2, a member of the Shal-type family, is the prominent A-type voltage-gated potassium channel expressed in hippocampal CA1 pyramidal neuron dendrites^1^. Kv4.2’s role in controlling of dendritic excitability impacts neuronal plasticity and contributes to learning and memory^3–5^. Kv4.2 activity remodels synaptic NMDA receptors by regulating the relative synaptic NR2B/NR2A subunit composition ratio at hippocampal synapses^6^. Ablation of Kv4.2 in mice abolishes the gradual reduction in GluN2B/GluN2A subunit ratio during post-natal development and results in a higher proportion of silent synapses in adulthood^7^. Aberrant Kv4.2 activity is also implicated in Autism Spectrum Disorder (ASD)^8^, temporal lobe epilepsy^9–11^, and Fragile X syndrome^12,13^.

Considerable evidence suggests that Kv4.2 channels function in macromolecular protein complexes with accessory subunits, including the K^+^ channel interacting proteins (KChIP1–4) and dipeptidyl peptidases 6 and 10 (DPP6 and DPP10)^14^. DPP6 is a type II transmembrane protein that increases Kv4.2 membrane expression and single channel conductance and accelerates the inactivation and recovery from inactivation of Kv4 subunit-containing channels^15,16^. In CA1 hippocampal pyramidal neurons, Kv4.2-mediated currents increase in density from the soma to distal dendrites^1^. However, this gradient is abolished in DPP6 KO mice^17^. In addition to its roles in modulating multiple aspects of Kv4.2 function, DPP6 appears to regulate hippocampal synaptic development independently of Kv4.2 (ref. ^18^). Recent studies have identified *DPP6* and *DPP10* as genes associated with autism^19^, amyotrophic lateral sclerosis^20,21^ and neurodegeneration^22^. Thus, the regulation of the Kv4.2-DPP6 complex may not only affect Kv4.2 channel activity but also influence Kv4.2-independent functions of DPP6. However, little is known about how the stability or composition of this complex is regulated.

In the present study, we report a Pin1-dependent mechanism that regulates the composition of the Kv4.2-DPP6 complex, neuronal excitability and cognitive flexibility. Pin1 is a prolyl isomerase that selectively binds to and isomerizes phospho-Ser/Thr-Pro (pSer/Thr-Pro) bonds^23^. pSer/Thr-Pro motifs in certain proteins can exist in two sterically distinct *cis* and *trans* conformations and Pin1 specifically accelerates the *cis*/*trans* conversion to regulate post-phosphorylation signaling^23^. Mis-regulation of Pin1 plays an important role in a growing number of pathological conditions including Alzheimer disease (AD), where it may protect against age-dependent neurodegeneration^24–27^. We identified Pin1 as a Kv4.2 binding partner via a TAP-MS pulldown assay. Subsequent biochemical studies revealed that Pin1-Kv4.2 binding is direct and via the canonical Pin1 binding motif. Stimuli including seizure induction and exposure to enriched, novel environments increased Kv4.2 phosphorylation at the Pin1 binding site T607 by p38 MAPK in the mouse cortex and hippocampus. Using biochemical and electrophysiological techniques, we showed that Pin1 activity is required for the dissociation of the Kv4.2-DPP6 complex and this action alters neuronal excitability. To confirm these observations, we generated a mouse line containing a Kv4.2 T607A (Kv4.2TA) mutation that abolished the phosphorylation and subsequent isomerization of an important C-terminal Pin1 motif. These mutant mice phenocopied those treated with pharmacological inhibitors of Pin1, which suggests a Pin1-dependent mechanism of Kv4.2 regulation. Intriguingly, Kv4.2TA mice exhibited normal initial learning but improved reversal learning in multiple behavioral tasks, introducing Pin1 isomerase regulation of Kv4.2 as a novel mechanism impacting cognitive flexibility.

## Results

### Pin1 binds to Kv4.2 at T607

**Fig. 1:**
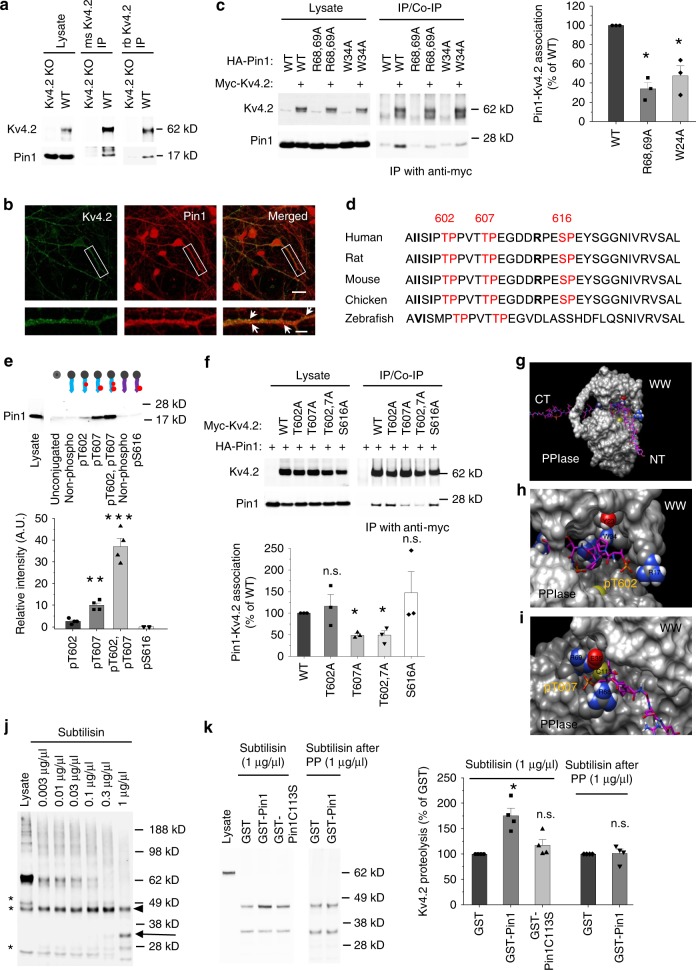
Pin1 binds to Kv4.2 at pT607 and elicits structural rearrangements in Kv4.2. **a** Pin1 co-immunoprecipitated with Kv4.2 in mouse brain lysates. Forebrain lysates from WT and Kv4.2 KO were immunoprecipitated with mouse (ms) or rabbit (rb) anti-Kv4.2 antibodies. Both total lysates and immunoprecipitates were blotted with anti-Kv4.2 or Pin1 antibodies. Data from three independent experiments. **b** Cultured hippocampal neurons (DIV 10) were immunostained with anti-Pin1 along with anti-Kv4.2. Pin1 co-localized with Kv4.2, indicated with arrows. Scale bars: 20 μm top panels, 5 μm bottom. Data from four coverslips in two independent experiments. **c**, Pin1 mutants reduced Pin1-Kv4.2 binding. Myc-Kv4.2 was co-transfected alongside HA-Pin1 with or without WW (W34A) or PPIase domain (R68, R69A) point mutants into HEK-293T cells. Kv4.2 was immunoprecipitated from detergent lysates with anti-Myc antibody. Samples were analyzed by western blotting with anti-HA and anti-Myc antibodies. *n* = 3 each group. **d** Alignment of Kv4.2 C-terminal sequences from various species. The putative Pin1 binding site is conserved. Bold residues show preferred Pin1 binding context. **e** Pin1 selectively binds to the phosoho-T607-containing Kv4.2 peptide. Synthetic Kv4.2-peptides were conjugated to Affi-Gel 15 Sepharose beads and incubated with lysate from HA-Pin1 transfected HEK-293T cells. *n* = 4 each group. **f** Kv4.2 T607A mutation significantly reduced Pin1 binding. HA-Pin1 and Myc-Kv4.2 mutants were co-transfected into HEK-293T cells. Pin1 co-immunoprecipitation with Kv4.2 was assayed. Kv4.2 T607 is required for Pin1 binding. *n* = 3 each group. **g** Molecular modeling of Kv4.2 phospho-peptide binding to Pin1. **h** Highlight of Kv4.2 pT602 peptide binding to the Pin1 WW domain. **i** Highlight of Kv4.2 pT607 peptide binding to the Pin1 PPIase domain. **j** Dose-dependent proteolysis of Kv4.2 by subtilisin. Asterisk, non-specific bands; arrowhead, 47kD band; arrow, 33kD band. Data repeated in two independent experiments. **k** Pin1 blocked Kv4.2 subtilisin digestion while Pin1C113S (an isomerase dead mutant) did not. Pin1 block was lost when Kv4.2 was dephosphorylated by Lambda protein phosphatase (PP). Quantification of the 47kD degradation fragment. *n* = 4 each group. Data was repeated in four independent experiments. Data are presented as mean ± SEM, *p < 0.05, ***p < 0.001, Paired *t*-tests.

Kv4.2 accessory subunits were identified by yeast two-hybrid screens and immunopurification over a decade ago^28,29^. Whether there are other Kv4.2 binding proteins that modulate Kv4.2 function is unknown. Here we took advantage of recently-developed Tandem Affinity Purification (TAP) combined with mass spectrometry (MS) techniques to identify Kv4.2 binding proteins in neurons and HEK-293T cells. We purified complexes of lentivirally expressed TAP-tagged Kv4.2 in cultured hippocampal neurons (Supplementary Fig. 1a). MS analysis showed interaction with the well-established Kv4.2 accessory subunits DPP6/10 and KChIP1-4, verifying the validity of our Kv4.2 TAP-MS screen (Supplementary Fig. 1b). This result is similar to the proteomic analyses of Kv4.2 complex in mouse brain using Kv4.2 antibody pulldown^30^. Using the same TAP technique to purify exogenously-expressed TAP-tagged Kv4.2 from HEK-293T cells, we identified Pin1 as a Kv4.2 binding partner (Supplementary Fig. 1c-f). As shown in the MS list, Kv4.2 has many intracellular binding partners when expressed in HEK-293T cells. However, the majority of the binding partners are protein synthesis and degradation machinery proteins (Supplementary Fig. 1c, d). This binding was confirmed by the co-immunoprecipitation (co-IP) of endogenous Pin1 with Kv4.2 in mouse brain lysates (Fig. 1a, uncropped images of all western blots are provided in the Supplementary Information file), and immunostaining of cultured hippocampal neurons revealed that Pin1 colocalized with Kv4.2 (Fig. 1b). Since Pin1 substrate binding requires phosphorylation, we showed that Kv4.2 binding to Pin1 is significantly reduced when it’s dephosphorylated by Lambda protein phosphatase (Supplementary Fig. 2a). To examine if Kv4.2 and Pin1 binding occurs via the canonical Pin1 binding interface, we employed the Pin1 WW domain mutant (W34A) and the PPIase domain mutant (R68A, R69A). When co-expressed with Kv4.2 in HEK-293T cells, both Pin1(W34A) and Pin1(R68A, R69A) mutants exhibited significantly reduced binding to Kv4.2 (Fig. 1c). Thus, the Kv4.2-Pin1 interaction appears to be direct and involves conventional Pin1 binding domains.

There are three S/T-P sites at the C-terminus of Kv4.2 that can be phosphorylated by extracellular signal–regulated kinases (ERKs)^31^. These phosphorylated S/T-P motifs might serve as putative Pin1 binding sites. Pin1 preferentially targets to a pSer/Thr-Pro motif that is surrounded by multiple upstream hydrophobic residues such as isoleucine, valine, tyrosine and/or phenylalanine, and a downstream arginine or lysine residue^23,32,33^. There are three isoleucine prior to the two T-P motifs and an arginine downstream (Fig. 1d), providing a better Pin1 binding context. Sequence alignment revealed that these S/T-P sites are conserved from zebrafish to human (Fig. 1d), suggesting a conserved function of Kv4.2 across the species. To identify the Pin1-binding site(s) in Kv4.2, we synthesized non-phospho- and phospho-Kv4.2 peptides containing T602, T607 or S616 and conjugated them with Affi-Gel 15 Sepharose beads. Peptide pulldown assays revealed that Pin1 binds weakly to the T602 phosphorylated peptide but strongly to the T607 phosphorylated peptide (Fig. 1e). Interestingly, Pin1 showed even stronger binding to the peptide with dual phosphorylation of T602 and T607 (Fig. 1e). Pin1 did not bind to the S616 phosphorylated peptide (Fig. 1e). Furthermore, co-IP studies in HEK-293T cell lysates using Kv4.2 mutants with abolished phosphorylation sites showed that T602A or S616A mutants did not affect Kv4.2-Pin1 binding while T607A or T602A/T607A mutants dramatically reduced the binding (Fig. 1f), which is consistent with the peptide pulldown assay (Fig. 1e). These data support the idea that Pin1 directly binds to Kv4.2 at T602 and T607 sites, where the latter site is involved in a greater degree of binding. Previous studies have shown that the PPIase domain of Pin1 is able to bind to the pS/T-P motif in addition to the WW domain^34,35^. pS/T-P motifs that have an additional P residue in the +1 position, pS/T-P-P, seem to be targeted by the WW domain but not the PPIase domain of Pin1 (ref. ^36^). Therefore, a substrate with multiple phosphate binding sites could allow for the simultaneous binding of multiple Pin1 domains^36,37^. Kv4.2-Pin1 binding modeled by an interplay of “manual” manipulation with the UCSF Chimera software showed the first pT-P-P binding to the Pin1 WW domain and the second pT-P binding to the Pin1 PPIase domain (Fig. 1g–i).

The Shal-type family contains three members: Kv4.1, Kv4.2, and Kv4.3. They are all expressed in the hippocampus but with different expression patterns^38^. We examined the possibility of Pin1 binding to other Shal-type family members. Human sequence alignment revealed that these S/T-P sites are conserved among the members (Supplementary Fig. 2b). The Pin1 binding context is also conserved except Kv4.1 lacking an arginine after the two T-P sites (Supplementary Fig. 2b). Co-IP showed that Pin1 binds to all the Kv4 members but not a non-Shal-type family member, Kv3.4, that does not contain the S/T-P motif at its C-terminal (Supplementary Fig. 2c). Pin1 binds to Kv4.2 and Kv4.3 better than Kv4.1 (Supplementary Fig. 2c), which is consistent with the lack of arginine in Kv4.1 (Supplementary Fig. 2b). Thus, Pin1 likely regulates all Kv4 members.

### Pin1 elicits structural rearrangements in Kv4.2

To verify whether the prolyl-isomerase activity of Pin1 induces conformational changes in Kv4.2, we performed a partial proteolysis assay on purified Kv4.2. This assay relies on the observation that Pin1-dependent structural changes impair proteolysis by subtilisin serine endopeptidase that is sensitive to substrate structure^39–41^. Myc-Kv4.2 was purified from HEK-293T cells that were transfected with myc-Kv4.2 construct and subjected to subtilisin digestion. Myc-Kv4.2 was dose-dependently degraded by subtilisin (Fig. 1j). It was mainly degraded into a 47kD fragment and a 33kD fragment (Fig. 1j). Incubation with GST-Pin1 prior to subtilisin significantly blocked the 47kD fragment degradation compared to GST control and GST-Pin1C113S, an isomerase dead mutant (Fig. 1k). Furthermore, the blockage effect of GST-Pin1 was abolished by Lambda protein phosphatase treatment before GST-Pin1 incubation (Fig. 1k). These data indicate that Pin1 is recruited by Kv4.2 in a phosphorylation-dependent manner and it promotes structural rearrangements.

### Kv4.2 T607 phosphorylation and Pin1 binding is dynamic

**Fig. 2:**
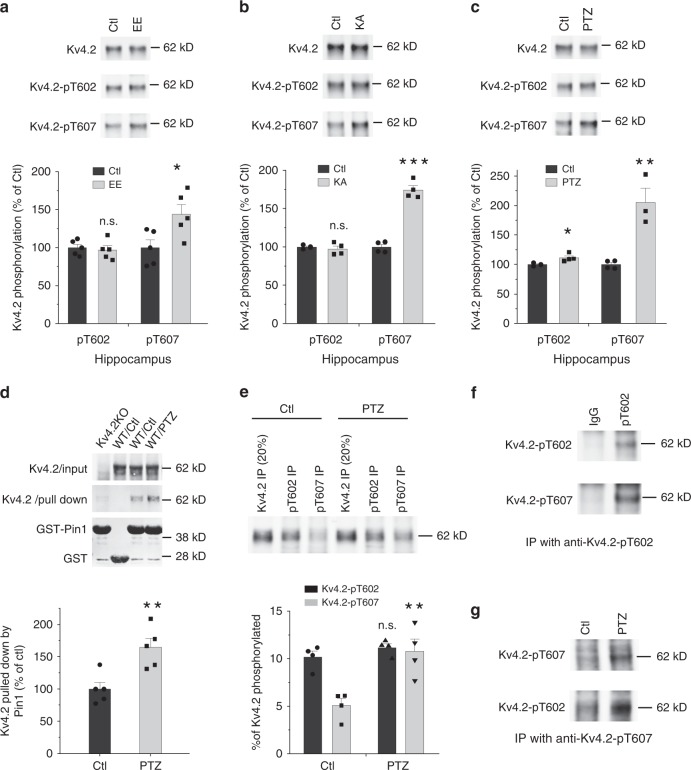
Enriched novel environment exposure and seizure induce Kv4.2 phosphorylation at a Pin1 binding site. **a** Enriched novel environment (EE, 1 h) induces phosphorylation of Kv4.2 at Thr607 but not Thr602 in mouse hippocampus. *n* = 5 in each group. *T*-test, *p < 0.05. **b** Kainic acid-induced seizure (25 mg/kg, i.p., 15 min) induces phosphorylation of Kv4.2 at Thr607 but not Thr602 in mouse hippocampus. *n* = 4 in each group. *T*-test, ***p < 0.001. **c** PTZ-induced seizure (50 mg/kg, i.p., 15 min) induces phosphorylation of Kv4.2 at Thr607 and Thr602 in mouse hippocampus. *n* = 4 in each group. *p < 0.05, *T*-test, **p < 0.01. **d** PTZ-induced seizure increases Pin1 binding to Kv4.2. GST or GST-Pin1-linked beads were incubated with brain lysates from mice subjected to saline or PTZ administration. *n* = 5 in each group. *T*-test, **p < 0.01. **e** Mouse brain lysates from WT mice w or w/o PTZ administration (50 mg/kg, i.p., 15 min) were incubated with excess anti-Kv4.2, anti-Kv4.2-pT602 or anti-Kv4.2-pT607 antibodies. Immunoprecipitation (IP) samples were blotted with Kv4.2 antibody. In WT mouse brains, pT607 Kv4.2 is almost half as abundant as pT602. However, PTZ administration increased the amount of pT607 until it reached a similar level as pT602. *n* = 4 for each group. *T*-test, **p < 0.01. **f** Mouse brain lysates from WT mice were incubated with excess anti-Kv4.2-pT602 or normal IgG antibodies. Immunoprecipitation (IP) samples were blotted with anti-Kv4.2 pT607 antibody. pT602 and pT607 dual phosphorylation was observed in mouse brain. Data was repeated in two independent experiments. **g** Mouse brain lysates from WT mice w or w/o PTZ administration (50 mg/kg, i.p., 15 min) were incubated with excess anti-Kv4.2-pT607 antibody. IP samples were blotted with anti-Kv4.2 pT602 antibody. PTZ-induced seizure increases the dual phosphorylation of T602 and T607 in mouse brain. Data was repeated in two independent experiments. Data are presented as mean ± SEM.

In order to further study the role of Kv4.2 phosphorylation at T602 and T607, we characterized phospho-T602 and phospho-T607-specific antibodies using site-specific mutations of Kv4.2 (Supplementary Fig. 3a, b) and Lambda protein phosphatase treatment (Supplementary Fig. 3c). Phosphorylation of Kv4.2 at T602 and T607 sites were detected in mouse brain lysates by western blot (Fig. 2a–c). To investigate the dynamic regulation of Kv4.2 phosphorylation, we exposed mice to a novel, enriched environment (EE) which has previously been shown to downregulate dendritic Kv4.2 function^42^. EE exposure induced T607 phosphorylation but not T602 phosphorylation in the mouse hippocampus (Fig. 2a). EE exposure induced similar changes in the cortex (Supplementary Fig. 4a).

Temporal lobe epilepsy has also been shown to decrease Kv4.2 availability^9,11^. Here we found seizure induced by kainic acid (KA) increased T607 phosphorylation but not T602 phosphorylation in the mouse hippocampus (Fig. 2b). Seizure induced by pentylenetetrazole (PTZ) showed increased T607 phosphorylation and T602 phosphorylation in the mouse hippocampus (Fig. 2c). It also increases Kv4.2 T607 phosphorylation but not T602 phosphorylation in the mouse cortex (Supplementary Fig. 4b). These data suggest that Kv4.2 T607 phosphorylation is dynamically regulated in the mouse brain. As Pin1 only binds to phosphorylated substrates, we hypothesized that the induction of Kv4.2 T607 phosphorylation would increase Pin1-Kv4.2 association. Accordingly, GST-Pin1 pulldown experiments revealed that PTZ-induced seizures significantly enhanced Kv4.2 pulldown by Pin1 (Fig. 2d).

We next investigated the prevalence of phosphorylated T602 and T607 in the mouse brain. Total, phospho-T602, and phospho-T607 Kv4.2 was immunoprecipitated by saturating specific antibodies and quantified by western blot. We found 10.20 ± 0.62% of Kv4.2 was phospho-T602 and 5.10 ± 0.71% was phospho-T607 in the mouse forebrain (Fig. 2e). Phospho-T607 increased to 10.78 ± 1.30% while T602 was un-altered (11.18 ± 0.41%) with seizure induction by PTZ (Fig. 2e). We also detected phospho-T607 Kv4.2 when brain samples were immunoprecipitated with phospho-T602 antibody (Fig. 2f). Furthermore, increased T602 phosphorylation was detected when immunoprecipitation was performed with the phospho-T607 antibody after PTZ administration (Fig. 2g). These data suggest that Kv4.2 is dually phosphorylated at sites T602 and T607 in the mouse brain and that their phosphorylation is regulated by neuronal activity.

### P38 phosphorylates Kv4.2 at T607

**Fig. 3:**
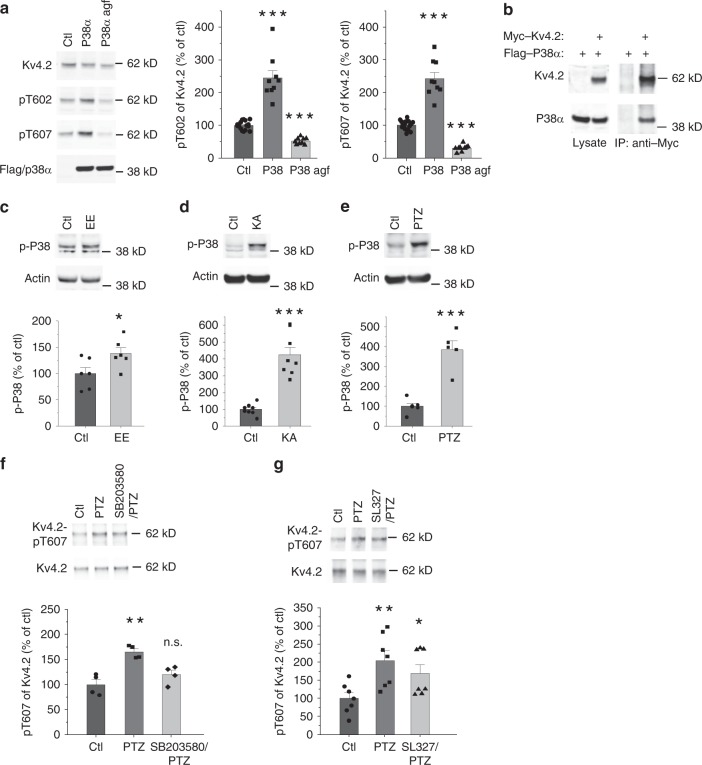
P38 phosphorylates Kv4.2 at a C-terminal Pin1 binding site. **a** P38 phosphorylates Kv4.2 at Thr602 and Thr607. Kv4.2 and p38α constructs (agf: p38 kinase dead mutant) were co-transfected into HEK-293T cells. Lysates were analyzed by western blotting with anti-pT602 and anti-pT607 specific antibodies. *n* = 15 for ctl, 9 for p38 and p38 agf. *T*-test, ***p < 0.001. **b** p38 binds to Kv4.2. Kv4.2 and p38α were co-transfected into HEK-293T cells. Detergent lysates were incubated with anti-Myc antibody and analyzed by western blotting with anti-Flag and anti-Myc antibodies. Data was repeated in two independent experiments. **c**, Enriched novel environment activates p38 in mouse hippocampus. *n* = 6 in each group. *T*-test, *p < 0.05. **d** KA-induced seizure activates p38 in mouse hippocampus. *n* = 8 in each group. *T*-test, ***p < 0.001. **e** PTZ-induced seizure activates p38 in mouse hippocampus. *n* = 6 for ctl and 5 for PTZ. *T*-test, ***p < 0.001. **f** SB203580, a potent p38 inhibitor (20 mg/kg, i.p., 15 min) blocked PTZ-induced phosphorylation of Kv4.2 T607 in mouse hippocampus. *n* = 4 in each group. *T*-test, **p < 0.01. **g** SL327, a selective MEK inhibitor (30 mg/kg, i.p., 15 min) did not block PTZ-induced phosphorylation of Kv4.2 T607 in mouse hippocampus. *n* = 7 in each group. *p < 0.05, **p < 0.01. *t*-test. Data are presented as mean ± SEM.

Kv4.2 T607 has previously been reported to be phosphorylated by ERK in vitro^31^. We verified this finding with co-expression assays in HEK-293T cells. Kv4.2 phosphorylation at T602 and T607 was increased when Kv4.2 was co-expressed with Erk1 or MEKDD (a constitutively active MEK mutant) (Supplementary Fig. 5a). However, these increases were small, which led us to consider other proline-directed kinases that could phosphorylate these two sites. We examined the individual effects of CDK5/p35, GSK3β and p38α on Kv4.2 phosphorylation. Among these proline-directed kinases, p38α had the most robust effect on Kv4.2 phosphorylation (Fig. 3a). Moreover, a p38α point mutant with abolished kinase activity largely blocked Kv4.2 phosphorylation when both constructs were expressed in HEK-293T cells (Fig. 3a). Exogenous p38α co-immunoprecipitated with Kv4.2 in HEK-293T cell lysates (Fig. 3b) which supports the notion that Kv4.2 is a substrate of p38α. Furthermore, EE exposure phospho-activated p38 in mouse hippocampus and cortex as reported by western blot using a phospho-p38 antibody (Fig. 3c, Supplementary Fig. 5b). Seizure induced by KA or PTZ also activated p38 in the hippocampus (Fig. 3d, e). These data are consistent with the effects of EE, KA, and PTZ on the induction of Kv4.2 T607 phosphorylation (Fig. 2a–c). Interestingly, the p38 inhibitor SB203580 blocked the induction of Kv4.2 phosphorylation by PTZ-induced seizure in the mouse hippocampus (Fig. 3f), while PTZ-induced Kv4.2 phosphorylation is only partly reduced by the MEK inhibitor SL327 (Fig. 3g). These findings suggest that p38 is the primary kinase responsible for the dynamic phosphorylation of Kv4.2 at the T607 site in mouse hippocampus.

### P38-Pin1-Kv4.2 pathway regulates Kv4.2-DPP6 complex formation

**Fig. 4:**
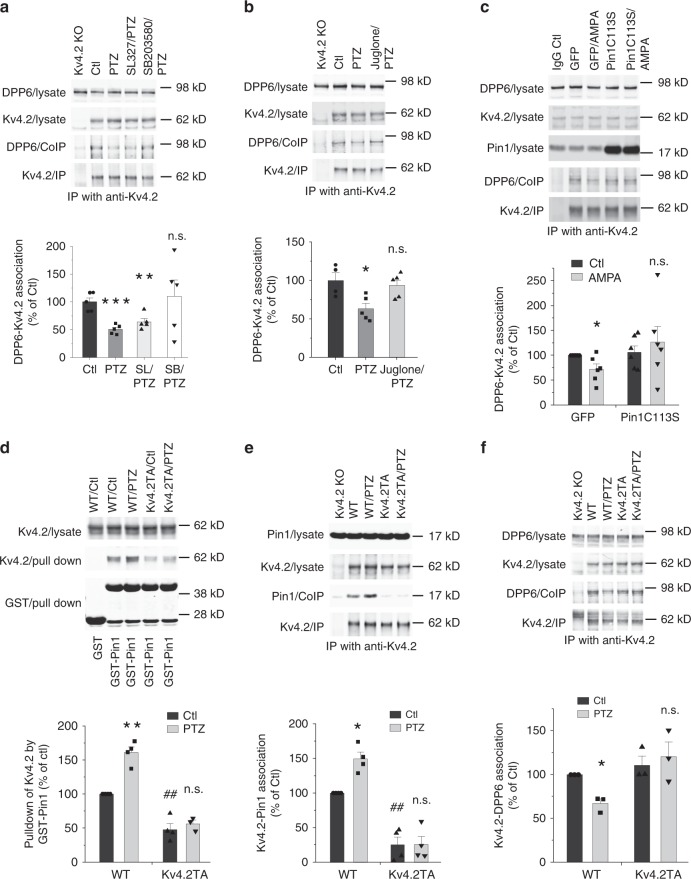
P38-Pin1 pathway regulates composition of the Kv4.2-DPP6 complex. **a** P38 inhibitor SB203580 blocked PTZ-induced Kv4.2-DPP6 dissociation while MEK inhibitor SL327 did not. Mouse forebrain lysates with or w/o SB203580 (20 mg/kg, i.p., 20 min) or SL327 (30 mg/kg, i.p., 20 min) or PTZ administration (60 mg/kg, i.p., 20 min) were immunoprecipitated with anti-Kv4.2 antibody. PTZ-injected mice showed decreased Kv4.2-DPP6 binding, blocked by preinjection of SB203580 but not SL327. *n* = 5 for each group. **b** Pin1 inhibitor juglone blocked PTZ-induced Kv4.2-DPP6 dissociation. Forebrain lysates with or w/o juglone (15 mg/kg, i.p., 15 min) or PTZ administration (60 mg/kg, i.p., 20 min) were immunoprecipitated with an anti-Kv4.2 antibody. PTZ-injected mice showed decreased Kv4.2-DPP6 binding while juglone-preinjected mice exhibited normal Kv4.2-DPP6 binding. *n* = 4 for ctl, 5 for PTZ and Juglone/PTZ. **c** Pin1C113S mutant blocked AMPA-induced Kv4.2-DPP6. Cultured cortical neurons infected with GFP or Pin1C113S lentivirus were treated with 50uM AMPA for 15 min and processed for immunoprecipitation with anti-Kv4.2 antibody. AMPA treatment reduced Kv4.2-DPP6 binding in GFP but not in Pin1C113S infected neurons. *n* = 6 for each group. **d** Seizure-induced Pin1-Kv4.2 association is abolished in Kv4.2TA mice. GST or GST-Pin1-linked beads were incubated with brain lysates from WT and Kv4.2TA mice with or without PTZ administration (60 mg/kg, i.p., 15 min). *n* = 4 WT/Ctl, WT/PTZ and Kv4.2TA/Ctl, *n* = 3 for Kv4.2TA/PTZ. **e** Seizure-induced Pin1-Kv4.2 association is abolished in Kv4.2TA mice. Forebrain lysates from WT and Kv4.2TA mice were immunoprecipitated with rabbit anti-Kv4.2 antibody, with or without PTZ administration (60 mg/kg, i.p., 15 min). Pin1-Kv4.2 association is induced by PTZ in WT but abolished in Kv4.2TA mice. *n* = 4 each group. **f** PTZ-induced Kv4.2-DPP6 dissociation is abolished in Kv4.2TA mice. Forebrain lysates from WT and Kv4.2TA mice with or w/o PTZ administration (60 mg/kg, i.p., 20 min) were immunoprecipitated with anti-Kv4.2 antibody. PTZ treatment decreased Kv4.2-DPP6 binding in WT but not in Kv4.2TA mice. *n* = 3 for each group. Data are presented as mean ± SEM. Paired *T*-test, **p < 0.01, **p < 0.01 vs ctl, ^##^*p* < 0.01 Kv4.2TA vs WT, ***p < 0.001.

Both the biophysical properties and surface expression of Kv4.2 are regulated by its auxiliary subunit DPP6 (refs. ^29,43^). We wondered if the Kv4.2-DPP6 complex is regulated by Kv4.2 phosphorylation and Pin1 activity. As PTZ-induced seizure enhanced Kv4.2 phosphorylation at T607 by p38 (Figs. 2, 3), we sought to determine if this seizure model also alters Kv4.2-DPP6 binding. From co-IP, we found that PTZ-induced seizure reduced Kv4.2-DPP6 binding in the mouse brain (Fig. 4a, b). This Kv4.2-DPP6 complex dissociation was blocked by the p38 inhibitor SB203580, but not the MEK inhibitor SL327 (Fig. 4a), suggesting that p38 is required for the dissociation of the Kv4.2-DPP6 complex. Furthermore, the PTZ-induced Kv4.2-DPP6 complex dissociation was blocked by the Pin1 inhibitor Juglone (Fig. 4b). In cultured mouse neurons, synaptic stimulation with α-amino-3-hydroxy-5-methyl-4-isoxazolepropionic acid (AMPA, 50 µM) for 15 min resulted in decreased Kv4.2-DPP6 binding, which was opposed by the expression of PinC113S, an isomerase dead mutant (Fig. 4c). These data suggest that Pin1 activity is required for the dissociation of the Kv4.2-DPP6 complex in response to neuronal activity.

To further study Pin1’s role in regulating Kv4.2 channel complexes, we created a Kv4.2 T607A mutant knock-in mouse (Kv4.2TA) where Thr607 was mutated to Ala using CRISPR-Cas9 techniques to prevent its phosphorylation and subsequent Pin1 binding (Supplementary Fig. 6a). The mice were identified by PCR followed by sequencing (Supplementary Fig. 6b). Kv4.2TA mice were born with expected Mendelian ratios, with no differences in mortality rate or weight of heterozygous or homozygous Kv4.2TA mice compared to WT littermates. There were no significant differences in the total protein expression of Kv4.2 in the hippocampus between WT and Kv4.2TA mice (Supplementary Fig. 6c). Kv4.2TA mice were also verified to have abolished Kv4.2 T607 phosphorylation by western blot (Supplementary Fig. 6c). Furthermore, the structure of the hippocampus appears normal by Nissl staining (data not shown). Additionally, the general distribution of Kv4.2 in apical dendrites appears to be unaltered in the hippocampus of Kv4.2TA mice relative to WT as detected by immuno-labelling (Supplementary Fig. 6d). To determine if Pin1 binding to Kv4.2 was impaired, we measured Kv4.2 pulldown by GST-Pin1 in Kv4.2TA and WT mouse forebrains with or without PTZ administration. Kv4.2 pulldown was reduced in Kv4.2TA mice compared to that of WT littermates (Fig. 4d) in basal conditions. Interestingly, PTZ-induced seizure did not increase Pin1 binding to Kv4.2 in Kv4.2TA mice as it did in WT littermates (Fig. 4d). We also performed the Kv4.2 and Pin1 Co-IP experiment under the same condition, and the result is consistent with the GST-Pin1 pulldown (Fig. 4e). These data indicate that Pin1-Kv4.2 binding is dynamically regulated in WT mice but abolished in Kv4.2TA mice. We next examined if the regulation of Kv4.2-DPP6 binding is altered in Kv4.2TA mice. Total DPP6 expression is normal in Kv4.2TA mice (Fig. 4f). However, Kv4.2-DPP6 dissociation by PTZ-induced seizure was abolished in Kv4.2TA mice (Fig. 4f). This data supports the notion that both Kv4.2 phosphorylation at T607 and Pin1 activity regulate Kv4.2-DPP6 complex formation.

### Pin1 activity and phosphorylation of Kv4.2 at T607 regulate neuronal excitability

**Fig. 5:**
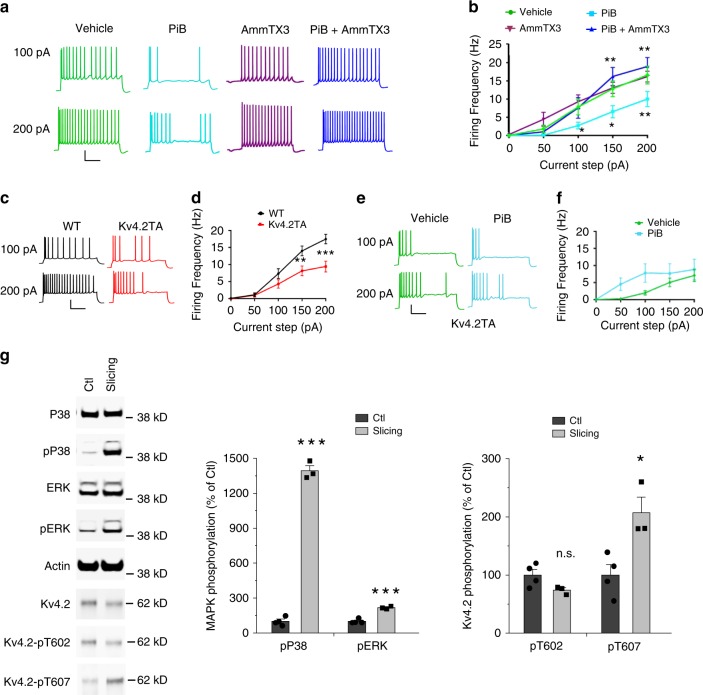
P38-Pin1-Kv4.2 pathway regulates neuronal excitability. **a**, **b** Pin1 inhibitor PiB (4 µM) reduces pyramidal cell excitability in WT mouse hippocampal brain slices. **a** Current steps at 100 pA and 200 pA result in reduced firing frequency with PiB application (teal) (*n* = 13) relative to vehicle (green) (*n* = 14). This reduction is rescued by co-application of AmmTX3 (250 nM) (blue) (*n* = 7). Scale: 30 mV / 250 ms. **b**, PiB significantly reduces AP firing frequency relative to vehicle in response to 100, 150, and 200 pA somatic current injections. This reduction is rescued by AmmTX3 application. Two-way ANOVA, *p < 0.05, **p < 0.01. **c**, **d** Mutation of Kv4.2 T607 Pin1 binding site phenocopies pharmacological inhibition of Pin1 in WT. **c** Pyramidal cells from Kv4.2TA slices display reduced firing frequency relative to WT over a range of increasing current injections. Scale: 30 mV/250 ms. **d** AP firing frequency is significantly reduced after 150 and 200 pA somatic current injections in Kv4.2TA (*n* = 22) relative to WT (*n* = 20), two-way ANOVA, **p < 0.01, ***p < 0.001. **e**, **f** Pin1 inhibition with PiB has no effect in Kv4.2TA mice. **e** Pyramidal cells from Kv4.2TA slices display similar AP firing patterns when treated with vehicle (*n* = 14) and PiB (*n* = 14). Scale: 30 mV/250 ms. **f** No significant changes are observed in Kv4.2TA pyramidal cell AP firing frequency with PiB exposure, Two-Way ANOVA, *p* > 0.05. **g** Brain slicing and recovery activates p38 and increases pT607 of Kv4.2. Adult mouse brains were either sliced as for electrophysiological recordings or dissected as for biochemical assays. Same brain regions were used. *n* = 4 for ctl and 3 for slicing. *T*-test. *p < 0.05, ***p < 0.001. Data are presented as mean ± SEM.

Substantial evidence supports a role for Kv4.2-containing A-type K^+^ channels and their associated auxiliary subunits in the regulation of the intrinsic excitability of CA1 pyramidal neurons. Along with other voltage-gated ion channels localized to the somatodendritic compartment of pyramidal cells, Kv4.2 contributes to the firing mode of the cell by regulating back-propagating action potential amplitude and the after-hyperpolarization of individual spikes in a train^1,44,45^. Thus, Pin1 regulation of the Kv4.2 channel complex could impact the excitability of hippocampal pyramidal neurons. To test whether Pin1 isomerization of Kv4.2 affects excitability, whole-cell somatic current-clamp recordings were performed in CA1 pyramidal neurons in adult mouse acute slices. We first asked if Pin1 regulates membrane excitability in WT mice. We utilized a Pin1 inhibitor, PiB, that has been shown to block the catalytic activity of Pin1 (ref. ^46^). Recordings were performed in the presence PiB (4 µM) following pre-incubation of slices with PiB included in the recovery solution. PiB significantly reduced the AP firing frequency of CA1 pyramidal cells compared to vehicle (0.1% DMSO) at each current step (Fig. 5a, b). Notably, PiB application resulted in a characteristic irregularity of spiking during current injections with prolonged intermittent pauses (Fig. 5a, b). To assess whether the PiB-induced reduction in excitability is mediated through a Kv4-dependent mechanism, we co-applied the Kv4-specific blocker, AmmTX3 (250 nM)^47^ along with PiB in the extracellular bath. Indeed, bath application of AmmTX3 reversed the suppressive effects of PiB alone (Fig. 5a, b), indicating that the effect of PiB on neuronal excitability is mediated by Kv4 channels. Additional electrical properties, including the resting membrane potential (RMP), membrane capacitance, and the shape of individual APs were unchanged between vehicle, PiB, and AmmTX3 treatments although PiB application did slightly reduce input resistance and increase rheobase (Table 1).

**Table 1:** Neuronal excitability and subthreshold membrane properties in WT and Kv4.2TA hippocampal CA1 pyramidal neurons with and without pharmacological treatment.

Parameter	WT	Kv4.2TA	WT with PiB	WT with vehicle (0.1%DMSO)	WT with AmmTX3	WT with PiB + AmmTX3	Kv4.2TA with vehicle	Kv4.2TA with PiB
RMP (mV)	−59.91 ± 0.85; *n* = 20	−60.75 ± 0.98; *n* = 22	−62.69 ± 1.07; *n* = 13	−60.21 ± 0.52; *n* = 14	−59.875 ± 1.30; *n* = 8	−63.43 ± 1.72; *n* = 7	−60.94 ± 0.89; *n* = 16	−60.79 ± 0.98; *n* = 14
Whole-cell capacitance (pF)	24.77 ± 4.4; *n* = 20	19.91 ± 1.2; *n* = 22	33.97 ± 9.12; *n* = 13	22.82 ± 3.62; *n* = 14	14.3 ± 1.29; *n* = 8	36.08 ± 8.93; *n* = 7	25.72 ± 6.0; *n* = 16	24.05 ± 5.6; *n* = 14
Input (MΩ)	212.2 ± 13.16; *n* = 20	196.4 ± 11.46; *n* = 22	178.99 ± 11.96; *n* = 13^a^	233.25 ± 11.15; *n* = 14	190.48 ± 15.50; *n* = 8	233.01 ± 13.39; *n* = 7	217.4 ± 13.3; *n* = 16	211.9 ± 21.65; *n* = 14
AP onset (ms)	11.60 ± 1.7; *n* = 20	12.17 ± 1.6; *n* = 22	16.19 ± 6.15; *n* = 13	6.94 ± 1.48; *n* = 14	5.24 ± 0.52; *n* = 8	12.19 ± 2.20; *n* = 7	9.33 ± 0.86; *n* = 16	10.5 ± 1.94; *n* = 14
AP threshold (mV)	−36.82 ± 0.82; *n* = 20	−37.1 ± 1.3; *n* = 22	−38.73 ± 1.07; *n* = 13	−37.09 ± 1.34; *n* = 14	−43.69 ± 1.41; *n* = 8^b^	−36.22 ± 2.35; *n* = 7	−36.49 ± 0.76; *n* = 16	−36.92 ± 0.94; *n* = 14
Rheobase (pA)	86.0 ± 5.9; *n* = 15	103.3 ± 8.5; *n* = −15	75.38 ± 8.52; *n* = 13^a^	52.14 ± 6.12; *n* = 14	53.13 ± 11.01; *n* = 8	64.28 ± 7.43; *n* = 7	96.88 ± 9.10; *n* = 16	83.93 ± 14.26; *n* = 14
AP amplitude (mV)	73.94 ± 2.8; *n* = 20	76.64 ± 1.5; *n* = 22	76.44 ± 1.85; *n* = 13	72.80 ± 2.12; n = 14	89.43 ± 0.72; *n* = 8^c^	69.13 ± 2.05; *n* = 7	68.65 ± 2.11; *n* = 16	72.2 ± 2.78; *n* = 14
Time to AP peak (ms)	0.888 ± .04; *n* = 20	0.784 ± .04	0.68 ± 0.04; *n* = 13	0.72 ± 0.05; *n* = 14	0.76 ± 0.03 *n* = 8	0.87 ± 0.10; *n* = 7	0.85 ± 0.04; *n* = 16	0.79 ± 0.41; *n* = 14
After-hyperpolarization (mV)	−1.20 ± 0.82; *n* = 20^b^	−4.7 ± .92; *n* = 22	−10.22 ± 1.32; *n* = 13	−8.14 ± 1.59; *n* = 14	−2.67 ± 1.14; *n* = 8	−4.78 ± 2.35; *n* = 7	−3.62 ± 1.06; *n* = 16	−5.69 ± 0.99; *n* = 14
AP half-width (ms)	1.56 ± .07; *n* = 20	1.36 ± .07; *n* = 22	1.17 ± 0.03; *n* = 13	1.33 ± 0.10; *n* = 14	1.56 ± 0.04; *n* = 8	1.55 ± 0.23; *n* = 7	1.40 ± 0.06; *n* = 16	1.43 ± 0.06; *n* = 14
Inter-spike interval (ms)	10.78 ± 1.5; *n* = 20^b^	15.96 ± 2.0; *n* = 22	23.67 ± 6.43; *n* = 13	13.25 ± 1.53; *n* = 14	36.32 ± 2.7; *n* = 8^c^	17.96 ± 3.22; *n* = 7	18.16 ± 2.41; *n* = 16	17.43 ± 2.79; *n* = 14
Peak firing frequency (spikes/sec)	17.46 ± 1.4; *n* = 20^c^	9.4 ± 1.6; *n* = 22	10.00 ± 2.06; *n* = 13^a^	16.59 ± 2.44; *n* = 14	16.17 ± 1.46; *n* = 8	18.95 ± 2.47; *n* = 7	7.04 ± 1.80; *n* = 16	8.83 ± 3.01; *n* = 14
SAG ratio (mv)	1.34 ± .02; *n* = 20	1.31 ± .03; *n* = 22	1.28 ± 0.02; *n* = 13	1.27 ± 0.10; *n* = 14	1.42 ± 0.01; *n* = 8^a^	1.24 ± 0.04; *n* = 7	1.28 ± 0.02; *n* = 16	1.30 ± 0.03; *n* = 14

^a^*p* < 0.01; ^b^*p* < 0.05; ^c^*p* < 0.001

We next assessed whether the Pin1-Kv4-dependent reduction in excitability through pharmacological manipulation was replicated by mutation of the Pin1 binding site T607 within Kv4.2. To test this, we performed whole-cell current-clamp recordings in hippocampal slices from WT and Kv4.2TA mice in regular ACSF. We found that the input/output curves of firing frequency displayed a rightward shift in Kv4.2TA cells relative to WT (Fig. 5c, d). At peak current injection (+200 pA), the average firing frequency in Kv4.2TA pyramidal cells was nearly half of that in WT cells (Fig. 5c, d). As with pharmacological blockade of Pin1 in WT, we noted an irregular AP spiking pattern in Kv4.2TA cells (Fig. 5c, d). We also found that the peak fast after hyperpolarization (fAHP) was significantly increased in Kv4.2TA (Table 1), which coincided with an overall, significant increase in the inter-spike interval (Table 1). Additional properties including RMP, membrane capacitance and AP shape were unchanged between the two mouse lines (Table 1). These data suggest a role for Kv4.2 phosphorylation at T607 in the regulation of neuronal excitability. Furthermore, we found that PiB exposure to Kv4.2TA slices did not significantly affect excitability in hippocampal pyramidal neurons (Fig. 5e, f), contrary to its effect in WT cells (Fig. 5a, b) and also note that reduced excitability of Kv4.2TA neurons was consistent in each experimental condition (Fig. 5). Therefore, pharmacological blockade of Pin1 did not augment any reduction in excitability induced by genetic manipulation of the Pin1 binding site, suggesting an important role for this specific Pin1-Kv4.2 interaction in its regulation of neuronal excitability.

The basal level of Kv4.2-DPP6 protein complex seems unaltered in Kv4.2TA mice compared to WT littermates in biochemistry experiments (Fig. 4f) whereas we found reduced neuronal excitability in Kv4.2TA mice (Fig. 5c, d). We hypothesized that the slicing and recovery process in recording experiments activates p38 and triggers Pin1-dependent changes. To examine this, we measured p38 phosphorylation in sliced brain in comparison with un-sliced brain. The results showed that slicing and recovery did not alter the expression of p38 protein (Ctl: 100 ± 6.23%; slicing: 93.30 ± 2.80%, *p* = 0.4262) and ERK (Ctl: 100 ± 3.85%; slicing: 100.36 ± 3.31%, *p* = 0.9492), but largely activates p38 (over 10 fold) and increases Kv4.2 phosphorylation at T607 (Fig. 5g). These data suggest that the slicing and recovery process before recording activated the p38-Pin1-Kv4.2 pathway, leading to the excitability changes in the Kv4.2TA mice.

### Pin1 activity and phosphorylation of Kv4.2 at T607 regulate A-current

**Fig. 6:**
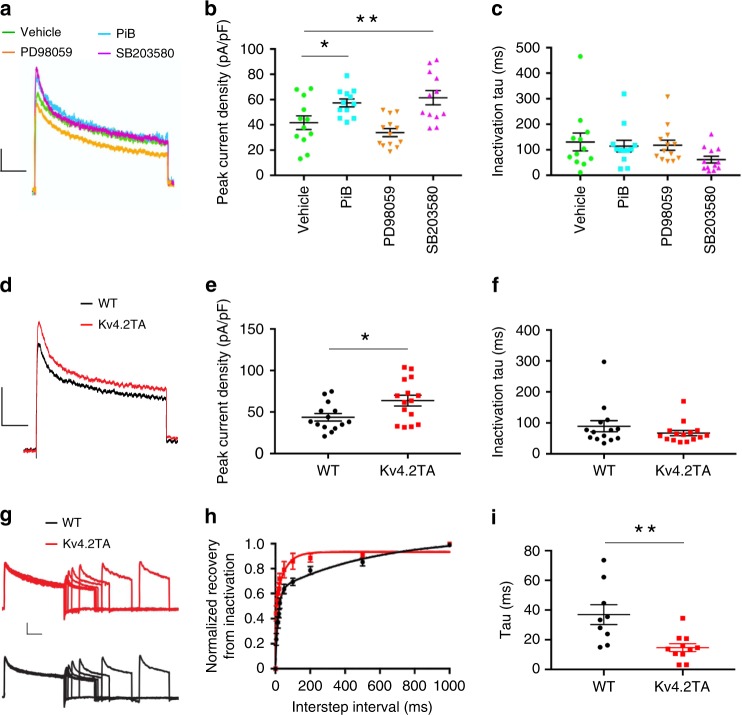
P38-Pin1-Kv4.2 pathway regulates A-current. **a**–**c** I_A_ recorded from outside-out somatic patches from CA1 pyramidal cells from WT mice. **a**, Trace of transient I_A._ inactivating I_A_ was isolated by subtracting total K^+^ measured from a step to +40 mV from a −120 mV pre-pulse from a subsequent step to +40 mv from −30 mV. Scale: 10 pA/100 ms. **b** I_A_ density in outside-out patches is significantly increased with PiB (*n* = 12) or SB203580 (*n* = 12) but not PD98059 (*n* = 13), ordinary one-way ANOVA (two-tailed), *p < 0.05, **p < 0.01. **c** No significant difference in decay kinetics was observed among the drug treatments and vehicle control, Kruskal–Wallis test (two-tailed), *p* > 0.05**. d** Trace of transient I_A_ in WT and Kv4.2TA mice. **e** I_A_ density in outside-out patches is significantly increased in Kv4.2TA mice (*n* = 15) relative to WT (*n* = 14), two-tailed unpaired *T*-test, *p < 0.05. **f** No significant difference in decay kinetics was observed between Kv4.2TA and WT, two-tailed Mann–Whitney test, *p* > 0.05. **g**–**i** I_A_ recovery from inactivation. **g** Sample traces of I_A_ recovery from inactivation in Kv4.2TA and WT. Scale: 20 pA/200 ms. **h** Normalized recovery curves from Kv4.2TA (*n* = 11) shows faster recovery relative to WT (*n* = 9). **i** Single-exponentials fitted to normalized recovery curves yielded significantly reduced tau in Kv4.2TA relative to WT, unpaired two-tailed *T*-test. **p < 0.01. Data are presented as mean ± SEM.

The reduced excitability observed in Kv4.2TA neurons and in response to pharmacological blockade of Pin1 in WT is suggestive of enhanced I_A_ in these cells. Studies of DPP6’s effect on Kv4.2 have revealed that DPP6 increases macro I_A_ amplitude and accelerates recovery from inactivation^17,48^. Since the Kv4.2-DPP6 complex is mis-regulated in Kv4.2TA mice (Fig. 4f), we anticipated disruption of Pin1-Kv4.2 interaction would alter I_A_. To test this, we performed voltage-clamp recordings from outside-out patches pulled from CA1 pyramidal somata. As in analysis of firing properties, we first measured I_A_ in WT slices exposed to Pin1 blocker, PiB (4 µM). We found that PiB exposure significantly increased I_A_ density relative to vehicle (.1% DMSO) (Fig. 6a, b). Additionally, as we identified p38 and MEK-mediated phosphorylation at the Pin1 binding site on Kv4.2, we tested their effect in facilitating Pin1 regulation of I_A_. We identified that pharmacological blockade of p38 (SB230580) also significantly increased I_A_ density relative to vehicle while MEK inhibition (PD98059) displayed no significant effect (Fig. 6a, b). Further, consistent with the observed effects on firing suggestive of enhanced I_A_ in Kv4.2TA mice, isolation of I_A_ revealed a significant increase in current density in patches pulled from Kv4.2TA cells relative to WT (Fig. 6d, e). Additionally, while changes in macro current inactivation, rise time and voltage-dependence of activation and inactivation were indistinguishable between the lines (Fig. 6c, Supplementary Tab. 1), a leftward shift in the normalized recovery from inactivation curve was identified in Kv4.2TA cells (Fig. 6h, i). Single exponentials fitted to the normalized recovery curves yielded a statistically significant reduction in the time constant of I_A_ recovery in Kv4.2TA cells, suggesting these channels recover more quickly from inactivation relative to WT (Fig. 6i). Importantly, pharmacological blockade of Pin1 had no effect on I_A_ in Kv4.2TA mice (Supplementary Fig. 7). Taken together, we show that block of p38 kinase and Pin1 results in enhanced I_A_ density in the soma of CA1 pyramidal cells. The T607A mutation occludes the effects of pharmacolocal Pin1 bockade on neuronal excitability and I_A_, supporting the notion that Kv4.2 phosphorylation at T607 and Pin1 isomerization of the Kv4.2 pT607-P bond regulate the intrinsic excitability of CA1 pyramidal neurons through the modulation of both Kv4.2 channel availability and recovery from inactivation kinetics. Furthermore, these data provide evidence that blocking Pin1-Kv4.2 interaction may increase the proportion of Kv4.2 channels in complex with DPP6.

### Kv4.2TA mice demonstrate enhanced cognitive flexibility

**Fig. 7:**
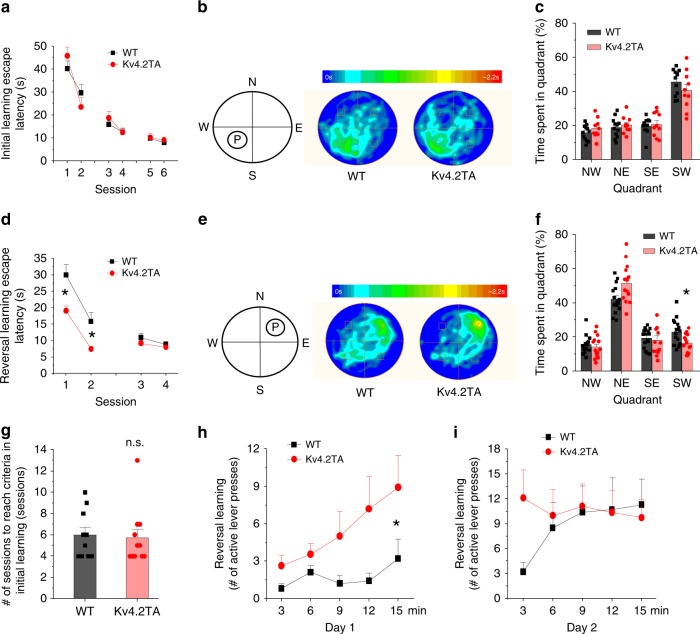
Improved reversal learning in Kv4.2TA relative to WT mice. **a–c** Kv4.2TA mice showed normal learning of the initial platform location in the Morris water maze task. Average escape latencies of four trials per round for WT (*n* = 16 mice) and Kv4.2TA mice (*n* = 14 mice) over a training period of 3 days (two rounds each day). **b** Swimming time heat maps during the probe trial of the WT and Kv4.2TA mice at day 5. **c** Time spent in each quadrant during the probe trial. **d**–**f** Kv4.2TA mice showed improved reversal learning in the Morris water maze. **d** Average escape latencies of four trials per round for WT and Kv4.2TA mice over a reversal training period of 2 days (two rounds each day). **e** Swimming time heat maps during the reversal probe trial of the WT and Kv4.2TA mice at day 8. **f** Time spent in each quadrant during the reversal probe trial. Two-way ANOVA, *p < 0.05. **g**–**i** Kv4.2TA mice displayed improved lever press reversal learning. **g** Number of trainings to reach criteria in initial learning. **h** Number of active lever press in the 1st day of reversal learning. *n* = 10 for WT, *n* = 11 for Kv4.2TA. Two-way ANOVA, *p < 0.05. **i** Number of active lever press in the 2nd day of reversal learning. *n* = 10 for WT, *n* = 11 for Kv4.2TA. Two-way ANOVA, *p < 0.05. Data are presented as mean ± SEM.

Kv4.2 KO mice have shown impairment in learning and memory^3^. In light of the physiological deficit and the accompanying biochemical changes, we sought to determine whether the disruption of Kv4.2 phosphorylation and Pin1 binding might alter any cognitive functions. In an open field test, Kv4.2TA mice showed normal locomotion, not significantly different from WT littermates (Supplementary Fig. 8a, b). In addition, Kv4.2TA mice displayed similar center vs perimeter time as WT littermates (Supplementary Fig. 8a, b), suggesting that their anxiety level was normal, too. We then employed the Morris water maze task to test hippocampal-dependent spatial memory. Both WT and Kv4.2TA mice showed similar performance in the training sessions (no main effect of genotype or genotype × session interaction, Fig. 7a) as well as in a probe trial (Fig. 7b, c). We then tested reversal learning by moving the hidden platform to the opposite quadrant of the pool. Kv4.2TA mice learned the new target location faster than WT littermates (effect of genotype: *F*_1,27_ = 11.92, *p* = 0.0018 session: *F*_3,81_ = 33.58, *p* = 1E-6; genotype × session: *F*_3,81_ = 3.71, *p* = 0.015; Fig. 7d). In addition, they spent more time in the new target quadrant and less time in old target quadrant during the reversal probe trial (Fig. 7e, f). This difference suggests that, although Kv4.2 T607 phosphorylation deletion did not affect the acquisition of spatial memory itself, it led to enhanced behavioral flexibility when the location of the platform was changed during the reversal learning. To investigate whether other forms of behavioral flexibility were also affected, we performed an operant reversal test^49^. In this task, Kv4.2TA mice showed normal acquisition of lever pressing behavior and no significant difference in reaching the learning criteria on a fixed ratio (FR1) schedule (Fig. 7g). In the following 5 random ratio (RR2) sessions, Kv4.2TA mice and WT littermates received similar numbers of rewards. However, when the reward lever was switched, Kv4.2TA mice exhibited faster reversal learning than WT littermates (Fig. 7h, i), as in the water maze (Fig. 7d). Kv4.2TA mice decreased inactive lever pressing and increased active lever pressing more rapidly than WT controls (effect of genotype: *F*_1,19_ = 5.017, *p* = 0.037; session: *F*_4,76_ = 4.110, *p* = 0.0045; Fig. 7h). Kv4.2TA mice reached the high level of active lever press while WT littermates barely started the reversal learning on the first day (Fig. 7h). On the second day of reversal learning, Kv4.2TA mice retained the high active lever pressing activity while WT littermates started reversal learning and caught up Kv4.2TA mice (effect of genotype x session: *F*_4,76_ = 4.514, *p* = 0.0025; Fig. 7i). These data show that disruption of Kv4.2 phosphorylation at T607 site and Pin1 binding/isomerization contributes to an enhanced rate of reversal learning suggesting improved cognitive flexibility.

## Discussion

**Fig. 8:**
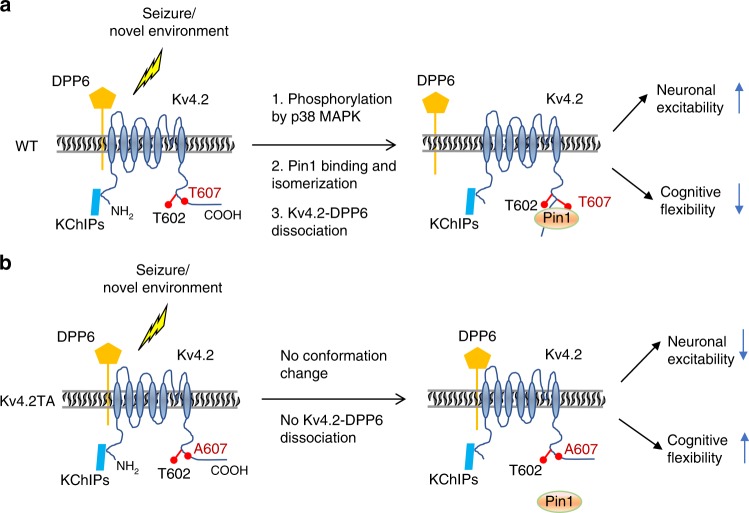
Working model of Pin1-dependent Kv4.2-DPP6 complex remodeling that underlies neuronal excitability and cognitive inflexibility. **a** In WT mice, stimulations such as seizure and exposure to a novel environment trigger the phosphorylation of Kv4.2 at T607, which allows Pin1 binding to pT602 and pT607 which subsequently isomerizes the pT607-P bond. This process changes the conformation of Kv4.2, which dissociates the Kv4.2-DPP6 complex and increases neuronal excitability and cognitive inflexibility. **b** In Kv4.2 TA mice, the 607 site is no longer phosphorylatable so that Pin1’s effect on Kv4.2 is abolished. The Kv4.2-DPP6 complex is stable, neuronal excitability is reduced, and cognitive flexibility is improved.

The present study describes a Pin1 isomerase-dependent mechanism that regulates the composition of the Kv4.2-DPP6 complex, neuronal excitability, and cognitive flexibility (Fig. 8). This mechanism occurs in a subset of neurons that are activated by neuronal activity or other stimulations. Pin1 was identified as a Kv4.2 binding partner by a TAP-MS assay in HEK-293T cells. As Pin1 is a cell proliferation regulator, examination of its substrates thus far has mainly focused on cell cycle proteins that play a pivotal role in cancer^50^. Increasingly, studies have shown that Pin1 isomerizes proteins in the brain such as APP^25^, Tau^51^, mGluR5 (ref. ^52^), PSD-95 (ref. ^41^), and CRMP2A^53^. We provide here the first report of a voltage-gated channel, Kv4.2, that is directly modified by Pin1. The effects of this modification were found to be important for neuronal and cognitive function. Pin1 is a peptidyl-prolyl *cis*-*trans* isomerase that catalyzes the isomerization of peptidyl-prolyl peptide bonds. Pin1 differs from other isomerases as it is, so far, the only known prolyl isomerase that specifically catalyzes isomerization of certain Ser/Thr-Pro bonds upon their phosphorylation^54^. Isomerization of Ser/Thr-Pro motifs is especially important because kinases and phosphatases specifically recognize the *cis* or *trans* conformation of the prolyl peptide bond of their substrates^55^ and phosphorylation further slows down the isomerization rate of proline^56^. Pin1 enhances the *cis*/*trans* conformational changes by reducing the free energy barrier, resulting in a markedly increased conversion rate up to 100- to 1000-fold^57^. The fast switch provides the correct conformation and precise timing for further activation and could be critical for modulating channel function in response to transient neuronal activity. Loss of phosphorylation essentially locks the channel into one confirmation. As described here, we show Pin1 acts as a molecular switch that mediates the activity-dependent regulation of a channel complex, thereby affecting neuronal excitability.

By using the TAP technique to purify exogenously-expressed TAP-tagged Kv4.2 from HEK-293T cells, many intracellular proteins were identified in addition to Pin1 (Supplementary Fig. 1c, d). The majority of the binding partners are protein synthesis and degradation machinery proteins, such as ribosomal proteins, eukaryotic initiation factors, proteasome subunits and ubiquitin-specific proteases (Supplementary Fig. 1c, d). This is reasonable since the exogenously expressed protein underwent active translation and degradation. Kv4.2-Pin1 binding is direct and requires critical amino acids that can bind to other substrates in the Pin1 WW (W34) and PPIase (R68, R69) domains. The Pin1 binding motif in Kv4.2 involves two adjacent pT-P motifs (TPPVTTP) which is similar to that of mGluR5 (TPPSPF)^52^. They even share the same pattern of phospho-regulation, i.e., the first T-P phosphorylation is not altered by stimulation while the second S/T-P phosphorylation is dynamic^52^. Interestingly, the first phosphorylation site of both proteins contains the T-P-P motif that is a better fit for binding the Pin1 WW domain while the second S/T-P motif binds to the catalytic domain^36^. This could be a common mechanism of how Pin1 regulates of dually phosphorylated proteins. In mGluR5, there is a second Pin1 binding motif in its C-terminal. The Kv4.2 mutant experiment (Fig. 2c) showed there is about 40% Pin1 binding left when the T602 and T607 sites were mutated, suggesting there may exist another Pin1 binding site. However, dynamic Pin1 binding to Kv4.2 is dependent on T607 site (Fig. 5d, e).

Although ERK can phosphorylate the three proline-directed sites (T602, T607, and S616) in vitro^31^, we found that p38 is a better proline-directed kinase for the T607 site of Kv4.2. Extensive and intensive studies highlighted the role of p38 in the stress responses, such as osmotic shock, UV irradiation, and inflammatory cytokines^58^. We have found exposure to an enriched novel environment and seizure induction by PTZ or KA activate p38 and increase Kv4.2 phosphorylation at T607 in mice (Figs. 2, 3). Importantly, we also found that p38-Pin1-Kv4.2 pathway regulates Kv4.2-DPP6 complex (Fig. 4) and neuronal excitability (Fig. 5). The mechanism how Pin1-elicited Kv4.2 conformation change leads to Kv4.2-DPP6 disassociation is interesting and needs to be elucidated in follow up studies. The p38 MAPK pathway is possible target for the treatment a number of neurodegenerative diseases, such as AD^59^. Thus, this Kv4.2 phosphorylation-Pin1 mechanism could be applied to treat pathological conditions and neurodegeneration diseases^22^.

As Kv4.2 containing channels are the primary carriers of the subthreshold, transient A-current, their impact on membrane excitability in rodent hippocampal pyramidal cells is well-documented^2,60^. We confirmed the significant contribution of T607 phosphorylation in mediating this influence as reduced excitability was observed in CA1 pyramidal cells of Kv4.2TA mice. This was further bolstered by our finding that the Pin1 blocker, PiB, decreased neuronal excitability in WT but not Kv4.2TA neurons, implying a floor effect in the mutant cells where the engagement of Pin1 and Kv4.2 is already maximally inhibited. Further, we show this reduction in excitability can be traced to alterations in I_A_. Pharmacological and genetic disruption of the p38-Pin1-Kv4.2 cascade resulted in enhanced I_A_ density in CA1 pyramidal somata. It is well established that modulation of Kv4.2 surface expression and/or kinetics/voltage-dependent properties, through the alteration of auxiliary subunits, impacts intrinsic excitability^43^. Although we identified a remarkable similarity in the firing properties of neurons from WT mice treated with Pin1 inhibitors and Kv4.2TA mice without pharmacological intervention, we did not observe significant alterations in subthreshold excitability in these mice relative to WT. This indicates the possibility that additional ion channels impacting sub-threshold membrane properties in CA1 pyramidal cells, such as Kv4.1 and Kv4.3 (Supplementary Fig. 2b, c), may be regulated by Pin1 as these interactions would also be impaired by broad Pin1 inhibition.

Our biochemistry data showed that Kv4.2-DPP6 dissociation is impaired in Kv4.2TA mice, indicating that the Kv4.2-DPP6 complex is more stable without phosphorylation at T607. In heterologous expression systems, association of DPP6 in the tripartite Kv4.2-KChIP-DPP6 complex leads to increased current density, faster recovery from inactivation, and more rapid inactivation^15,16^. Our voltage-clamp recordings support this notion as we identified increased I_A_ density in Kv4.2TA mice compared to WT littermates and a non-significant trend toward faster macro current decay, which was also observed with p38 inhibition. Interestingly, the recovery from inactivation kinetics in Kv4.2TA mice displayed a shift to faster recovery, consistent with more channels in complex with DPP6, further supporting our hypothesis. Moreover, that the Kv4-specific blocker AmmTX3 (250 nM) occluded the effects of PiB on WT mice, suggests that the PiB-induced reduction in excitability is mediated by Kv4 channels that are associated with DPP6, since the high-affinity blockade of Kv4 channels by AmmTX3 depends on the presence of DPP6 (ref. ^47^). It is intriguing that constitutive knockout of DPP6 does not result in significant alterations in somatic I_A_ (ref. ^17^); however, evidence is suggestive of homeostatic compensation in the soma of DPP6 KO mice, which preserves relative excitability^17^. It is likely this compensation is absent in Kv4.2TA mice given our findings that firing properties are also significantly altered.

The basal level of Kv4.2-DPP6 protein complex is not altered in Kv4.2TA mice compared to WT littermates in biochemistry experiments (Fig. 4f). However, we saw reduced neuronal excitability (Fig. 5c, d) and increased I_A_ (Fig. 6d, e) in Kv4.2TA mice compared to WT littermates. This difference likely results from technical differences between biochemical and electrophysiological experiments. To determine this, we measured p38 phosphorylation in sliced brain in comparison with un-sliced brain. The result showed that slicing and recovery largely activates p38, and Kv4.2 phosphorylation at T607 is also increased after slicing (Fig. 5g). These data suggested that slicing and recovery process before recording has already activated p38-Pin1-Kv4.2 pathway, and the data is consistent with our hypothesis. Taken together, our data demonstrate that Pin1 regulates the composition of the Kv4.2-DPP6 complex and neuronal excitability. These changes may then impart additional, so-far undetermined, downstream effects in the neuron.

Cognitive flexibility is the ability to appropriately adjust one’s behavior according to a changing environment. Greater cognitive flexibility is associated with favorable outcomes throughout the lifespan. Here we showed that reduced neuronal excitability unexpectedly left initial learning and memory intact and improved reversal learning in Kv4.2TA mice. Cognitive flexibility has previously been associated with both NMDAR- and mGluR-dependent long term depression^61–64^. Further research is required to attribute a cellular function to the enhancement in reversal learning observed in Kv4.2TA mice. Cognitive inflexibility is observed in various psychiatric disorders such as autism spectrum disorder (ASD)^65^, schizophrenia^66^, suicidal ideation^67^, and anxiety and mood disorders^68^. Considering that both Kv4.2 and DPP6 are implicated in such psychiatric disorders^8,22,69^, the stability of the Kv4.2-DPP6 complex might be a common factor of pathophysiology. It will be interesting to examine if the T607A mutation can rescue cognitive inflexibility in mouse models of psychiatric or neurodegenerative disorders.

Taken together, our results reveal that disrupting the activity-dependent isomerization of Kv4.2 by Pin1 stabilizes the Kv4.2-DPP6 complex and improves cognitive flexibility. Stabilization of the Kv4.2-DPP6 complex might represent a promising strategy for enhancing adaptive cognitive behavior and correcting maladaptive cognitive deficits in a number of neuropsychiatric conditions.

## Methods

### Expression constructs

The human Myc-DDK-Kv4.2 construct was purchased from Origene (RC215266). All of the other expression constructs were made by PCR. Internal deletions and point mutations were generated using either the QuikChange Site–Directed Mutagenesis Kit (Stratagene) or the megaprimer method. PCR products were cloned into expression vectors pGEX 4T2 (Pharmacia) and pRK5 (Genentech), with Myc, flag or HA tags as we reported previously^52^. All constructs were verified by sequencing.

### Chemicals

All chemicals were purchased: KA (Sigma, K0250), PTZ (Sigma, P6500), SB203580 (Tocris, 1202), PD 98059 (Tocris, 1213), SL327 (Tocris, 1969), S-AMPA (Tocris, 0254), Juglone (Millipore, 420120), PiB (Sigma, B7688), AmmTX3 (Alomone, 305). For injections, KA and PTZ were dissolved in saline; SB203580, SL327 and Juglone were dissolved in DMSO and 10% Tween 80.

### Antibodies

Mouse anti-Kv4.2 (NeuroMab, 75-016) was used at 1:2000 for western blot, 1:200 for immunostaining, Rabbit anti-Kv4.2 (Sigma, P0233) was used at 1:2000 for western blot, rabbit anti-Kv4.2 (Sigma, HPA029068) was used at 1:200 for staining, pT602 (Santa Cruz, SC-16983-R) was used at 1:1000 for western blot, pT607 (Santa Cruz, SC-22254-R) was used at 1:500 for western blot, Pin1 (Santa Cruz, SC-46660) was used at 1:100 for staining, 1:1000 for western blot, Pin1 (Millipore, 07-091) was used at 1:3000 for western blot, p38 (Cell Signaling, 9212 s) was used at 1:1000 for western blot, p-p38 (Cell Signaling, 4511 s) at 1:1000 for western blot, DPP6 (Abcam, 41811) was used at 1:2000 for western blot, Myc (Millipore, 05-419) was used at 1:10000 for western blot, HA(Santa Cruz, SC-805) was used at 1:1000 for western blot, Actin (Sigma, A-1978) was used at 1:10000 for western blot; Alexa Fluor 488 goat anti-mouse (Invitrogen, A-11029) was used at 1:500; Alexa Fluor 488 goat anti-rabbit (Invitrogen, A-11034) was used at 1:500; Alexa Fluor 555 goat anti-mouse (Invitrogen, A-21424) was used at 1:500; Alexa Fluor 555 goat anti-rabbit (Invitrogen, A-21429) was used at 1:500; Alexa Fluor 680 goat anti-mouse (Invitrogen, A-21057) was used at 1:10000; Alexa Fluor 680 goat anti-rabbit (Invitrogen, A-21076) was used at 1:10000; IRDye 800CW goat anti-mouse (Licor, 926-32210) was used at 1:10000, IRDye 800CW goat anti-rabbit (Licor, 926-32211) was used at 1:10000.

### Mouse models

Kv4.2TA mice were generated using CRISPR-Cas9 techniques. Briefly, CRISPR sgRNA (CCTGTCGTCGCCTTCTGGGG) was made by in vitro transcription using ThermoFisher’s sgRNA synthesis service. B6D2F1 mice (JAX Stock No. 100006) were used as embryo donors for this study. For the injection step, sgRNA (20 µg / ml), Cas9 mRNA (100 µg/ml, Trilink Biotechnologies) and corresponding single strand oligos with mutations (100 µg/ml) were injected into the cytoplasm of fertilized eggs, which then were cultured overnight in M16 medium. Those embryos that reached the two-cell stage of development were implanted into the oviducts of pseudo-pregnant surrogate mothers (CD-1, Charles River). Mice born to these foster mothers were genotyped by PCR amplification followed by DNA sequencing to identify mice with correct mutations.

Mice were group housed in plastic mouse cages with free access to standard rodent chow and water. The colony room was maintained at 22 ± 2 °C with a 12 hr: 12 h light: dark cycle. Kv4.2TA mice were backcrossed at least three generations onto C57/Bl6J mice. All animal procedures were performed in accordance with guidelines approved by the National Institute of Child Health and Human Development Animal Care and Use Committee and in accordance with NIH guidelines.

### Cell culture and transfection

HEK-293T cells used in biochemistry experiments were obtained from Dr. Paul Worley’s lab^70^. HEK-293T cells were cultured in DMEM medium containing 10% FBS. Transfections were performed with X-tremeGENE 9 according to the manufacturer’s specifications. Cells were harvested about 40 h after transfection.

### Neuronal culture

Mouse hippocampal neuron cultures from embryonic day 18 (E18) pups were prepared as reported previously^71^. In all, 1 × 10^6^ neurons were added to each well of a six–well plate (Corning) with cover slips coated with poly–L–lysine. Growth medium consisted of Neurobasal medium (Invitrogen) supplemented with 5% FBS (Hyclone), 2% B27, 1% Glutamine (Invitrogen), 100 U/mL penicillin, and 100 U / mL streptomycin (Invitrogen). Neurons were fed twice per week with glia-conditioned growth medium. DIV14-17 neurons were used for biochemistry experiments. Rat hippocampal neuron cultures from embryonic day 18 (E18) pups were prepared similarly as above. DIV14-17 neurons were used for the immunostaining experiment.

### Tandem affinity purification-mass spectrometry (TAP-MS) assay

Kv4.2 was subcloned into the TAP tag vector that was obtained from Agilent (pCTAP, #240102). TAP-tagged Kv4.2 was then subcloned into the lentivirus vector (Dr. Paul Worley’s lab) to generate TAP-Kv4.2-IRES-GFP Lentivirus. TAP-Kv4.2-IRES-GFP Lentivirus and IRES-GFP control Lentivirus were generated using a standard protocol^72^. Rar hippocampal neuron cultures from embryonic day 18 (E18) pups were prepared as described above. Neurons were infected by TAP-Kv4.2-IRES-GFP Lentivirus or IRES-GFP control Lentivirus on the day of the culture and harvested at DIV14. TAP-Kv4.2 was purified using the TAP purification kit from Agilent (#240107) with some modifications. The samples were run on 10% SDS-PAGE gel (Novex/Invitrogen). The gels that contains the protein samples were excised, separated into high molecular and low molecular weight samples, and sent to the Taplin Mass Spectrometry Facility at Harvard University for in-gel digestion using trypsin and mass spectrometric analysis.

### Peptide pulldown

The following peptides were synthesized: Non-Phospho for T602 and T607: KAIISIPTPPVTTPEGDDR; pT602: KAIISIP-pT-PPVTTPEGDDR; pT607: KAIISIPTPPVT-pT-PEGDDR; pT602, pT607: KAIISIP-pT-PPVT-pT-PEGDDR; Non-Phospho for S616: KEGDDRPESPEYSGG; pS616: KEGDDRPE-pS-PEYSGG. The peptides were conjugated to Affi-Gel 15 (Bio-Rad) according to the manufacturer’s instructions. Peptide-linked Affi-Gel was incubated for 3 h at 4 °C with Pin1 protein that was expressed in HEK-293T cells, and then washed clear for western blot analysis.

### Co-immunoprecipitation and immunoprecipitation assays

Mouse brain tissues or HEK-293T cells were used in co–immunoprecipitation assays as previous reported in ref. ^70^. For Kv4.2-Pin1 co-IP experiment, co-IP buffer (1 X PBS, pH 7.4, with 0.8% Triton X–100, phosSTOP and Complete™ EDTA–Free protease inhibitors) were added (1:20 for brain tissues and 400 µl for a 6-well of HEK-293T cells), and the samples were sonicated. After centrifugation, the supernatant was mixed with 2–3 µg of Kv4.2 (NeuroMab, 75-016, or Sigma, P0233) or myc (Millipore, 05-419) antibodies for 3–4 h at 4 °C. Next, 40 µl of protein G magnetic beads (Bio-Rad, 161-4023) was added for an additional 2 h or overnight. The protein beads were washed three times with co-IP buffer. The protein samples were eluted with SDS loading buffer and analyzed by gel electrophoresis and western blotting. For Kv4.2-DPP6 co-IP experiment, co-IP buffer (1 X PBS, pH 7.4, with 1% Triton X–100, 0.2% Chaps, phosSTOP and Complete™ EDTA–Free protease inhibitors) were added (1:20 for brain tissues), and the samples were sonicated. After centrifugation, the supernatant was mixed with 3–4 µg of Kv4.2 antibody (NeuroMab, 75-016) for 3 h at 4 °C. Next, 50 µl of 1:1 protein G–Sepharose slurry (GE Healthcare, 17-0886-02) was added for an additional 3 h. The protein beads were washed three times with IP buffer. The protein samples were eluted with SDS loading buffer and analyzed by gel electrophoresis and western blotting. For Kv4.2 phosphorylation detection, brain tissues sonicated in co-IP buffer (1 X PBS, pH 7.4, with 1% Triton X–100, phosSTOP and Complete™ EDTA–Free protease inhibitors). After centrifugation, the supernatant was mixed with 3 µg of Kv4.2 (NeuroMab, 75-016) antibody for 3 h at 4 °C. Next, 50 µl of 1:1 protein G–Sepharose slurry (GE Healthcare, 17-0886-02) was added for an additional 3 h. The protein beads were washed three times with IP buffer. The protein samples were eluted with SDS loading buffer and analyzed by gel electrophoresis and western blotting.

### Molecular modeling

Models of the bound phosphotyrosine-proline sequence motifs were developed from published structures. These models were primarily based on PDB: 2N10 (ref. ^73^) for the WW domain, and PDB: 2Q5A^74^ for the Catalytic domain. Modeling was done by an interplay of “manual” manipulation with UCSF Chimera^75^ and energy minimization (to prevent steric overlap and optimize salt-bridges and hydrogen bonds) with CHARMM^76^. Images were generated with UCSF Chimera.

### Subtilisin proteolysis

Myc-Kv4.2 (co-transfected with p38) was expressed in HEK293T cells and purified with myc-magnetic beads (Thermo Scientific, #88842). Kv4.2 Phosphorylation at T602 and T607 after purification was measured by saturated phospho-specific and total Kv4.2 antibody pulldown combined with western blot. About 2/3 of purified Myc-Kv4.2 was phosphorylated at T607 and was used for proteolysis. Equal amount of Myc-Kv4.2 were then incubated with 100 ng of either GST, GST-Pin1, and GST-Pin1C113S in a buffer containing 50 mM HEPES, pH 7.5, 100 mM NaCl, 1 mM MgCl_2_, supplemented with phosphatase inhibitors. After 30 min incubation at room temperature, reaction mixtures were cooled on ice, and subtilisin (Sigma-Aldrich, P5380) was added for a further 1 min on ice. The reaction was stopped by the addition of boiling sample buffer, and the proteolytic fragments were resolved by 4–12% SDS-PAGE and visualized by western blot analysis.

### Western blot and quantification

Protein samples were mixed with 4x LDS sample buffer (Invitrogen NP0007) and 10x sample reducing agent (Invitrogen NP0007) to a final concentration of 1×. Samples were loaded on 4–12% Bis-Tris gradient gel (Invitrogen 12-well, NP0322; 15-well, NP0323). The proteins were transferred to Immobilon-FL PVDF membrane (EMD Millipore, IPFL00010). The membrane was blocked with Odyssey blocking buffer (Li-COR, 927-40000) for 1 h at room temperature, followed by incubation with primary antibody in PBS overnight at 4 °C. The membrane was then washed with PBST (PBS, pH 7.4, and 0.1% Tween-20) three times and incubated with secondary antibody in PBS for another hour. After three washes with PBS, the membrane was scanned using an Odyssey imaging system (LI-COR) according to the manufacturer’s protocol. Quantification of western blots was carried out using the gel analysis function in ImageJ within the linear range of detection which is determined by using serial dilutions of a representative sample.

### Immunostaining

Cultured hippocampal neurons (DIV10) were fixed with 4% PFA, and permeabilized with 0.2% Triton X-100 in PBS. Cells were then blocked with 10% horse serum at RT for 1 h and then incubated with mouse anti-Pin1 antibody (Santa Cruz, SC-46660, 1:100) and rabbit Kv4.2 antibody (Sigma, HPA029068, 1:200) at 4°C overnight. After washing, cells were incubated with anti-mouse-555 and anti-rabbit-488 secondary antibodies at RT for 1 h. After washing, cells were then mounted on slides with anti-fade mounting medium containing 4′,6-diamidino-2-phenylindole (DAPI, Invitrogen, P36962) and imaged using a Zeiss 710 laser scanning confocal microscope equipped with a ×63 objective.

### Acute hippocampal slice preparation

For all electrophysiological recordings, adult male (5–7 weeks) mice were used. Mice were anesthetized in isoflurane and decapitated. Brains were removed and washed with ice-cold sucrose cutting solution. The sucrose solution was made up of the following (in mM): 60 NaCl, 3 KCl, 28 NaHCO_3_, 1.25 NaH_2_PO_4_, 5 Glucose, 0.5 CaCl_2_, 7 MgCl_2_. Brain hemispheres were dissected and mounted following a 45° cut of the dorsal cerebral hemisphere(s). Modified transverse slices (300 μm) were made by a Leica VT1200S vibrating microtome in ice-cold sucrose that was continuously bubbled with carbogen (95% O_2_/5% CO_2_). Slices were recovered at 32 °C in sucrose solution for 30 min at which time the solution temperature was slowly lowered to room temperature where it remained for the remainder of the recording day.

### Whole-cell current-clamp recordings

Following a 1-hour recovery in sucrose cutting solution, hippocampal slices were transferred to a recording chamber submerged in artificial cerebral spinal fluid (ACSF) with the temperature maintained at 33 °C (±1 °C). The ACSF contained the following (in mM): 125 NaCl, 2.5 KCl, 25 NaHCO_3_, 1.25 NaH_2_PO_4_, 25 Glucose, 2 CaCl_2_, 1 MgCl_2_(pH 7.4). The recording chamber was continuously perfused with carbogen-bubbled ACSF at a rate of 2–3 mL/min. Somatic whole-cell patch-clamp recordings were performed on identified somata of hippocampal CA1 pyramidal neurons. Pyramidal neurons were identified using infrared Differential Interference Contrast (DIC) on an upright Leica Axioskop 2. Cells were patched with 3–6 MΩ borosilicate glass pipettes pulled from a Narishige vertical puller and filled with K^+^ Gluconate-based intracellular solution consisting of the following (in mM): 20 KCl, 125 K-Gluconate, 1 EGTA, 4 NaCl, 4 Na_2_ATP, 0.3 NaGTP, 10 HEPES, 10 Phosphocreatine with pH adjusted with KOH and HCl to a final value of 7.25–7.30 and an osmolarity of 290–300 mOsm.

Firing properties were measured from whole-cell recordings in the conditions described above unless otherwise noted (described in detail below). All data were recorded with a Multiclamp 700b amplifier (Molecular Devices) and a Digidata 1440 A digitizer. Signals were low-pass filtered at 5 kHz and digitized at 10 kHz using Clampex 10.7 software and were acquired in bridge balance mode to compensate series resistance. Liquid junction potential was not corrected for. Subthreshold membrane properties were measured after initial break-in in order to avoid alteration in equilibrium potentials and dialysis as a result of solution exchange. Whole-cell capacitance and series resistance were measured from Multiclamp 700B commander. A voltage step of −10 mV was initiated, and the decay tau of the whole-cell capacitive transient was used to calculate these parameters. Resting membrane potential was measured after switching to *I* = 0. Any recordings where series resistance exceeded 25 MΩ or resting membrane potential was greater (more depolarized) than −55 mV were discarded. Input resistance was calculated as the slope of the I–V curve in response to current steps from −50 to 50 pA in 50 pA steps (three steps in total). To evoke APs in patched CA1 pyramidal neurons, square 1 s current pulses were elicited in 50 pA steps with current injections ranging from −200 to +200 pA in 50 pA steps. In some cases, for instance in the measurement of rheobase, smaller step sizes of 20 pA were used to enhance the resolution of the average minimum current magnitude required to elicit APs. Three sweeps at each magnitude were elicited and the average response of the three sweeps was used for each cell. All measures of AP waveform were taken from the first spike in a train in response to a 200 pA injection, and inter-spike measurements, including inter-spike interval and after-hyperpolarization amplitude, were recorded between the first two spikes in a train elicited by a 200 pA square current injection (see associated figures).

### Outside-out somatic patch voltage-clamp recordings of A-current

A-current was recorded in voltage-clamp mode from outside-out patches pulled from somata of identified pyramidal cells in hippocampal slices. Outside-out patch pipettes (3–6 MΩ) were filled with K^+^ Gluconate-based intracellular solution consisting of the following (in mM): 20 KCl, 125 K-Gluconate, 1 EGTA, 4 NaCl, 4 Na_2_ATP, 0.3 NaGTP, 10 HEPES, 10 Phosphocreatine. All recordings were performed at room temperature (24–25 °C). Tetrodotoxin citrate (TTX) (500 nM), Gabazine (2 µM), and CNQX (2 µM) were added to the extracellular bath (ACSF) in order to block voltage-gated Na^+^ channels and ligand-gated channels. A-current density was calculated using a standard subtraction protocol. In short, a pre-pulse step to −120 mV for 600 ms from a holding potential of -65 mV was initiated to relieve inactivation of A-type K^+^ channels. Voltage was stepped to +40 mV for 500 ms to measure total outward K^+^ current. A subsequent sweep consisted of a pre-pulse step to −30 mV to inactivate the transient current followed by a step to +40 mV to measure the non-inactivating current. Peak amplitude of the non-inactivating current was subtracted from total outward K^+^ current amplitude offline to isolate I_A_. Leakage and capacitive currents were digitally subtracted. Peak amplitude was normalized to current density by dividing by patch capacitance, which was measured following formation of the outside-out patch configuration and was analyzed with custom written code in MATLAB version R2018a (MathWorks). Inactivation kinetics were measured by fitting a single exponential to each isolated I_A_ current trace and were analyzed with Graphpad Prism. Traces are averages of 10-30 sweeps. Recovery from inactivation was calculated using a double voltage-step (−120 to +30 mV) protocol with increasing interstep intervals between steps. Interstep intervals were (in ms): 5, 10, 15, 20, 25, 50, 100, 200, 500, and 1000. Recovery from inactivation was calculated as the ratio of the peak amplitude of the second current trace relative to the point where the initial A-current was fully inactivated and was normalized to the peak amplitude of the initial A-current. Single exponentials were fitted to the non-linear fitted curves of normalized recovery from inactivation and the taus from each individual recovery curve were averaged and compared. Voltage-dependent activation was recorded using a voltage step protocol consisting of a 600 ms prestep to −120 mV, followed by a series of 13 steps (10 mV each from −80 to 40 mV). Leakage and capacitive currents were digitally subtracted. The same protocol but with a 600 ms prestep from -65 mV to -30 mV was used to record non-inactivating currents, which were offline subtracted from the overall K^+^ currents to obtain inactivating A-type K^+^ currents. Currents were then converted into conductances, normalized to peak conductance at 40 mV, plotted against the holding voltage and fitted with a Boltzman-function to obtain V_1/2-activation_ and k_activation_. Voltage-dependent inactivation was recorded using a voltage-step protocol consisting of a series of 14 presteps (each 600 ms and 10 mV, from −120 to 10 mV) followed by an activating voltage step to 20 mV. Leakage and capacitive currents were digitally subtracted. Peak currents were measured for each step and the I/V relationship was fitted with a Boltzmann function to obtain V_1/2-inactivation_ and k_inactivation_. All data were recorded with a Multiclamp 700b amplifier (Molecular Devices) and a Digidata 1440A digitizer, were digitized at 10 kHz and low-pass filtered at 2 kHz using a Bessel filter.

### Behavioral assays

#### Open field task

Novelty-induced locomotor activity was assessed in a novel open-field square arena (50 × 50 cm) constructed of white Plexiglas as previously described^77^. Mice were acclimated to the testing room for at least ~10 min and then placed in the arena and left to explore freely for 30 min. Sessions were performed once a day for two successive days. The distance traveled and time spent in different areas of the maze were measured. Results were analyzed with “ANY-maze” software (ANY-maze, Wood Dale, IL, USA). Data were compiled from two independent experimental cohorts. Male mice used in the first and second cohorts were ~8.5–10 and ~15–16 weeks old, respectively.

#### Morris water maze

The Morris water maze task was performed to evaluate hippocampus-dependent spatial navigation learning and memory^78,79^. The water maze consisted of a 120 cm circular pool (depth 50 cm), filled ~40 cm deep with 20–22 °C water containing a 10 cm wide square platform. External high contrast cues were placed on the interior of the pool above the water surface to aid with spatial navigation. Trials were video recorded and scored by ANY-maze software (ANY-maze, Wood Dale, IL, USA) for measures including latency to find the hidden platform, total distance traveled, and swim speed. The latency to the platform of the training trials was measured manually with a stopwatch.

General mouse handling was performed as follows: Mice were acclimated to the testing room for at least ~1 h before testing. Each mouse was placed into the water maze facing the wall in one of four possible quadrant positions, which was pseudo-randomly varied by training session. Mice were given 60 s to find the platform and a ~15 s platform rest interval. If a mouse was unable to find the platform in the allocated time, it was gently guided to the platform and allowed to rest for ~15 s. Mice were then patted dry with a cloth and put back into a warm cage for ~15 s after each trail. For data analyses, a latency time of 60 s was ascribed to mice that failed to reach the platform without guidance.

On Day 1 mice were trained in the visible platform version of the Morris water maze task to assess general swimming and visual ability. The platform was ~1 cm above the clear water surface with a red flag placed on the platform to increase its visibility. Each mouse underwent two visible platform training sessions and the location of the platform was varied between sessions. No significant difference in escape latency was apparent between genotypes. The water was made opaque with nontoxic white and red paint between Days 1 and 2. On Day 2 through Day 4 (sessions 1–6), mice were trained for the Hidden Platform protocol where the flag was removed from the platform and additional water was added to the pool to submerge the platform ~1 cm below the surface. Mice were given a total of 24 training trials (4 trials per session, two sessions per day for three successive days). On Day 5, the platform was removed, and mice underwent a 60 s probe trial to determine the amount of time spent exploring the target quadrant. On Day 6 and Day 7, mice were trained for reversal learning (2 sessions per day for 4 total sessions) where the Hidden Platform was moved to the opposite quadrant. On Day 8, the platform was removed, and the mice underwent a 60 s probe trial to determine the amount of time spent exploring the target quadrant. ~16–17-weeks-old male mice were used. Data were compiled from two independent experimental cohorts.

#### Lever press

Operant reversal learning was performed as previously described in ref. ^49^. Mice were food restricted to 85–90% of their free-feeding weight over several days prior to testing and throughout the experiment. Mice were trained to lever press on an FR1 schedule in daily sessions with an endpoint of 30 rewards or 30 min. When they collected all of the rewards in the allotted time for 3 consecutive days, animals progressed to RR2 sessions lasting 25 min, during which they could earn unlimited rewards. After 5 training days, levers were reversed such that the reinforced lever became non-reinforced and vice versa. Mice were given daily 15-minute reversal learning sessions for 5 days.

### Statistical analysis

Biochemistry and behavior data were analyzed by Origin 2018b by two–tailed Student’s t test and two–way ANOVA, respectively. Electrophysiology data were analyzed by GraphPad Prism 7 (7.0d). For all measures of I_A_ in outside-out somatic patches the experimenter was blinded to the genotype. For measures of firing properties, the experimenter was aware of these conditions. Sample sizes were not predetermined with any statistical methods but were chosen based on numbers reported in similar publications in the field. All statistical tests were two-tailed. Specifically, for electrophysiological analysis of I_A_ and AP shape in WT and Kv4.2TA mice and pharmacological analysis (PiB treatment) in Kv4.2TA, unpaired t-test (with Welch’s correction) was used (passed normality testing). For electrophysiological analysis of the pharmacological impact on I_A_ in WT slices a One-way ANOVA (ordinary), or One-Way ANOVA on Ranks (Kruskal–Wallis) was used and were corrected for multiple comparisons with Dunnett’s test (ordinary) or Dunn’s test (Ranks) respectively. The use of parametric or non-parametric analysis was determined after testing for normal distribution in the data using the D’Agostino & Pearson normality test for all electrophysiological data (alpha Level = 0.05). Non-parametric statistics were used if the data failed normality testing. For all analysis of pharmacological treatment effects in WT, significance was probed relative to control (vehicle). All analysis of firing frequency in response to sequential current steps, a two-way ANOVA with Sidak’s post hoc test was used. All the data are presented as mean ± SEM.

### Reporting summary

Further information on experimental design is available in the Nature Research Reporting Summary linked to this paper.

## Supplementary information

Supplementary Information

Peer Review

Reporting summary

## Data Availability

PDB:2N10, PDB:2Q5A, and other data that support the findings of this study are available from the corresponding authors in reasonable request. The source data underlying all figures and tables are provided as a Source Data file.

## source data

source data
