# Supplementary material for: Activity-dependent isomerization of Kv4.2 by Pin1 regulates cognitive flexibility
Source: Nat Commun. 2020 Mar 26;11:1567. doi: 10.1038/s41467-020-15390-x (PMC7099064; doi:10.1038/s41467-020-15390-x)

a

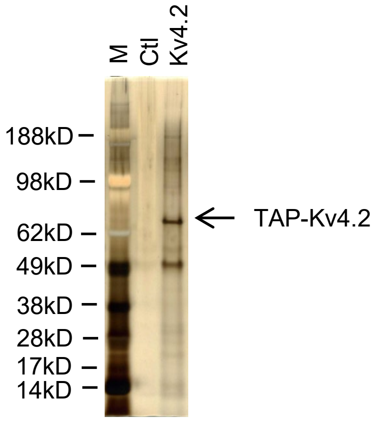

b

Protein	Number of peptides (Unique)	Number of peptides (Total)	% of aa sequence coverage
Kv4.2	27	126	30.00%
Kv4.1	7	11	14.92%
Kv4.3	15	17	25.19%
DPP6	30	40	33.99%
DPP10	26	26	29.27%
KChIP1	9	9	22.47%
KChIP2	5	5	16.30%
KChIP3	6	7	35.94%
KChIP4	12	16	43.60%

e

Pin1 sequence:

MADEEKLPPG WEKRMSRSSG RVYYFNHITN ASQWERPSGN SSSGGKNGQG EPARVRCSHL LVKHSQSRRP SSWRQEKITR <u>TKEEALELIN GYIQK</u>IKSGE EDFESLASQF SDCSSAKAR<u>G DL GAFSRGQM</u> <u>QKPFEDASFA</u> <u>LRTGEMSGPV</u> <u>FT DSGIHIIL</u> <u>RTE</u>

f

Position	Peptide	∆corr	Xcorr
81-95	TKEEALELINGYIQK	0.362	4.21
120-127	GDLGAFSR	0.185	2.20
128-142	GQMQKPFEDASFALR	0.482	3.63
143-163	TGEMSGPVFTDSGIHIILRTE	0.509	4.54
143-161	TGEMSGPVFTDSGIHIILR	0.595	5.07

c

Gene symbol	# of peptides (Unique)	# of peptides (Total)
EIF3A	53	56
DHX9	52	54
CAND1	41	42
SPTAN1	39	39
EIF4G1	35	39
EIF3B	33	33
USP9X	32	33
MYO6	30	30
EIF4G2	29	31
PSMD1	29	29
MYH9	27	27
CYFIP1	25	27
MYH10	25	26
KIF5B	25	25
EIF3C	24	25
SPTBN1	22	22
AP3B1	21	21
MYO1C	18	18
ATP2B1	18	18
UBE3C	18	18
USP7	16	16
PSMD2	15	21
MYO1B	15	15
PPP6R3	13	13
ARHGEF2	13	13
CLASP2	11	19
USP24	11	11
PIK3C2A	11	11
AP2A1	10	11
USP48	10	10
PIK3R4	10	10
USP10	10	10
ARHGAP17	9	10
KIF1A	9	9
MYO1D	9	9
AP1B1	8	8
KIF11	8	8
UBA52	7	52
KIF5A	7	7
PI4KA	7	7
CAND2	7	7
USP19	6	6
ARHGEF1	6	6
CYFIP2	6	6
LARS2	6	6
USP5	5	11
USP15	5	6
PPP6R1	5	6
UBE2O	5	5
UBE4B	5	5
KIF23	5	5
MYO1E	5	5
CLASP1	5	5
EIF4G3	5	5
URB1	5	5
ITCH	4	4
XPO6	4	4
RAB3GAP1	4	4
PTPN23	4	4
PPP4R1	3	3
AP2A2	3	3
ARFGEF1	3	3
EIF2AK4	3	3

d

Gene symbol	# of peptides (Unique)	# of peptides (Total)
RPS18	25	39
RPS9	17	37
RPS19	16	30
RPS16	16	30
RPS7	15	20
RPS25	13	16
RPS13	12	14
PSMB2	12	12
RPS14	11	14
PSMB1	11	14
PSMB4	11	13
PSMA2	11	13
ARF1	11	13
PMVK	11	11
PSMA3	10	10
PKM	9	13
RAB7A	9	12
RPL10	9	11
RAB1A	9	9
RPS11	9	9
RPL9	8	12
RHOA	8	9
EIF5A	8	9
RAB13	8	8
RPS24	7	12
UBE2M	7	7
RAC1	7	7
DTYMK	7	7
UBA52	6	10
PIN1	6	6
RPS8	6	6
PARK7	6	6
RPS5	6	6
MYL6	6	6
ATP5F1	5	6
RAN	5	6
PTGES3	5	5
AP1S2	5	5
RHEB	5	5
EIF4A1	4	6
RAB7L1	4	5
EEF1A1	4	4
GSTK1	4	4
RHOG	4	4
ARPC4	4	4
ARL6	4	4
RAB6A	4	4
RAB21	4	4
RPL35	4	4
CMPK1	4	4
RPL18	3	4
SURF4	3	4
EIF3K	3	3
AP1S1	3	3
ARF6	3	3
ARF4	3	3
VAMP7	3	3
ARF5	3	3

Supplementary Figure 1

Supplementary Figure 1. Pin1 is a binding partner of Kv4.2.

a, Silver stain showing the Kv4.2 complex isolated with a TAP-Kv4.2 pulldown assay in cultured hippocampal neurons. M: marker; Ctl: control. **b,** The numbers of unique and total peptides, and the percent (%) amino acid sequence coverage for each protein that are presented in hippocampal neurons. **c,** High molecular weight proteins that are identified by TAP-Kv4.2 pulldown assay in HEK-293T cells. **d,** Low molecular weight proteins that are identified by TAP-Kv4.2 pulldown assay in HEK-293T cells. **e,** Amino acid sequence coverage obtained for the (human) Pin1 protein from HEK-293T cells. **f,** Pin1 protein sequence report. Xcorr: Sequest cross-correlation score; Δcorr: Xcorr difference between the top ranked and next best sequence.

a

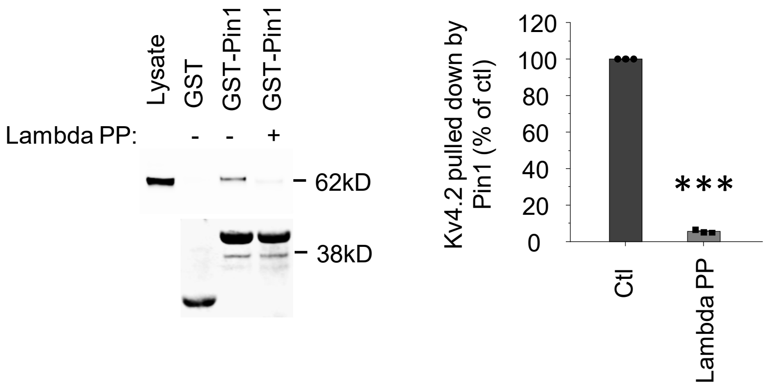

b

Kv4.1 RDFVAA**II**S**I**PTPPANTPDESQPSSPGGGGRAGSTLRNSSLGTPCLFPETVKISSL

Kv4.2 PYVTTA**II**S**I**PTPPVTTPEGDD**R**PESPEYSGGNIVRVSAL

Kv4.3 SQITTA**II**S**I**PTPPALTPEGES**R**PPPASPGPNTNIPSIASNVVKVSVL

c

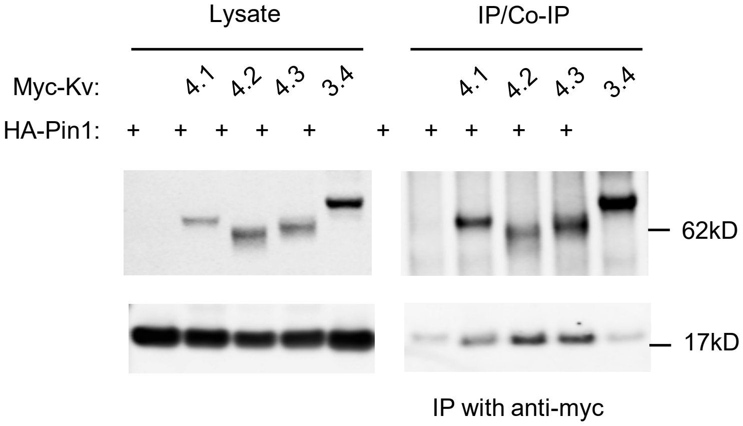

Supplementary Figure 2

Supplementary Figure 2. Pin1 binding to Kv4.2 is phosphorylation-dependent and Pin1 binds to A-type voltage-gated potassium channels.

a, Lambda protein phosphatase treatment significantly reduced Kv4.2 pull-downed by GST-Pin1, suggesting Pin1 binding to Kv4.2 is phosphorylation-dependent. n = 3 each group. Paired t-test, ***p < 0.001. **b,** Alignment of C-terminal sequences of human A-type voltage-gated potassium channels. Note that the putative Pin1 binding site is conserved among Kv4 family members. Bold residues showed a preferred Pin1 binding context. **c,** Pin1 co-immunoprecipitated (co-IP) with Kv4 members but not Kv3.4 which doesn't contain the putative Pin1 binding site when expressed in HEK-293T cells. Data was repeated in three independent experiments. Data are presented as mean ± SEM.

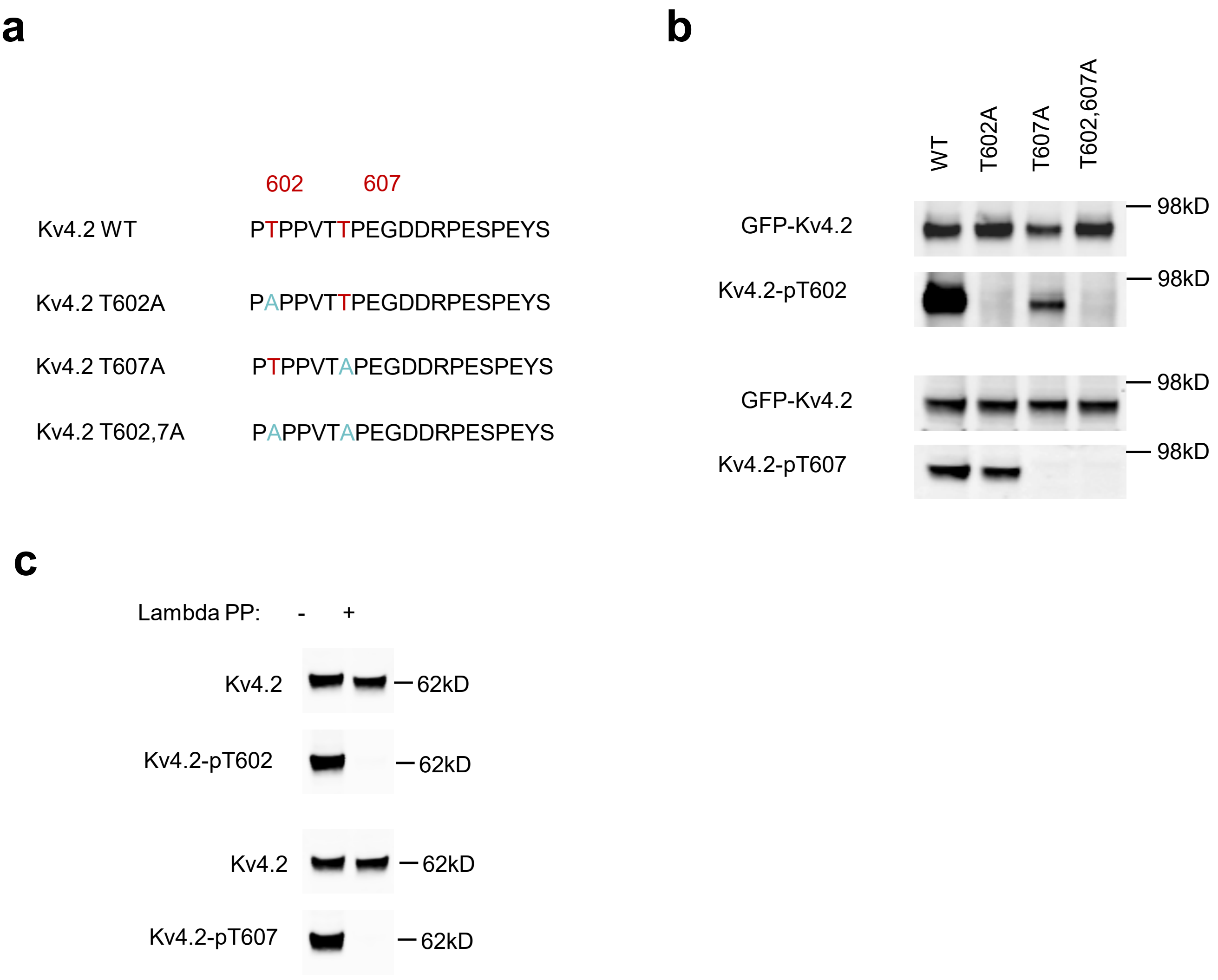

Supplementary Figure 3

Supplementary Figure 3. Characterization of Kv4.2 phosphorylation-specific antibodies with Kv4.2 mutants.

a, Sequences of mutated Kv4.2 regions. **b,** Kv4.2 and its point mutants were transfected into HEK-293T cells. Detergent lysates were analyzed by Western blotting with phospho-specific antibodies. Phospho-T602 and phospho-T607 antibodies of Kv4.2 specifically recognize pT602 and pT607 respectively. Data was repeated in two independent experiments. **c,** Kv4.2 were treated with or without Lambda protein phosphatase (PP) before western blot. Phospho-T602 and phospho-T607 antibodies of Kv4.2 can only detect Lambda PP untreated Kv4.2. Data was repeated in three independent experiments.

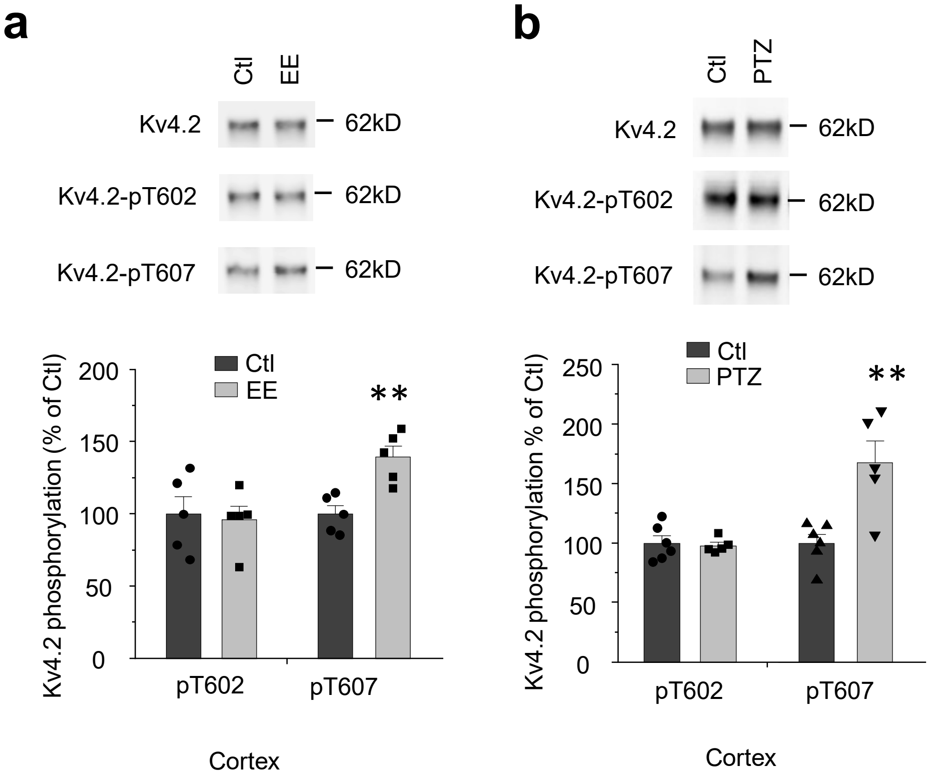

Supplementary Figure 4

Supplementary Figure 4. Enriched novel environment and seizure induce Kv4.2 phosphorylation in mouse cortex.

a, Enriched novel environment (EE, 1hr) induces phosphorylation of Kv4.2 Thr607 but not Thr602 in mouse cortex. $n = 5$ in each group. $^{**}p < 0.01$. **b,** PTZ-induced seizure (50 mg / kg, i.p., 15 min) induces phosphorylation of Kv4.2 Thr607 but not Thr602 in mouse cortex. $n = 6$ forctl and 5 for PTZ. T-test, $^{**}p < 0.01$. Data are presented as mean ± SEM.

a

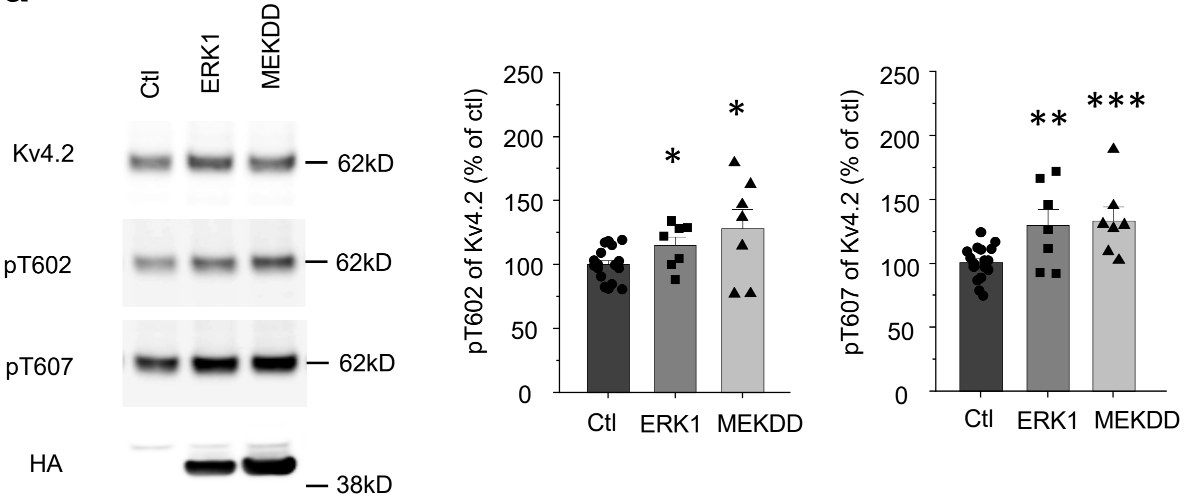

b

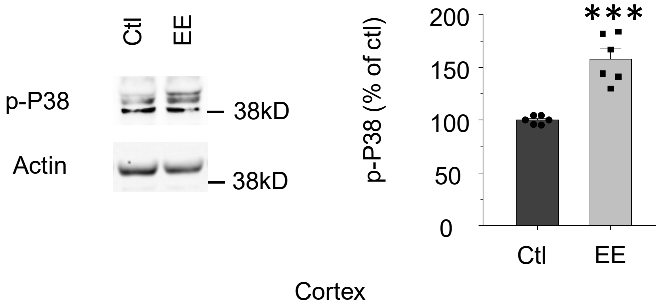

Supplementary Figure 5

Supplementary Figure 5. ERK phosphorylates Kv4.2 in HEK-293T cells and enriched novel environment activates p38 in mouse cortex.

a, Kv4.2 and ERK1 or MEKDD (a constitutively active MEK mutant) constructs were co-transfected into HEK-293T cells. Lysates were analyzed by western blotting with anti-pT602 and anti-pT607 specific antibodies. n = 16 for ctl, 7 for ERK1 and MEKDD. $*p < 0.05$, $**p < 0.01$, $***p < 0.001$. ERK1 and MEKDD increase Kv4.2 phosphorylation at T602 and T607, but to a small degree. **b,** EE induces the phospho-activation of p38 in mouse cortex. n = 6 in each group. T-test, $***p < 0.001$. Data are presented as mean ± SEM.

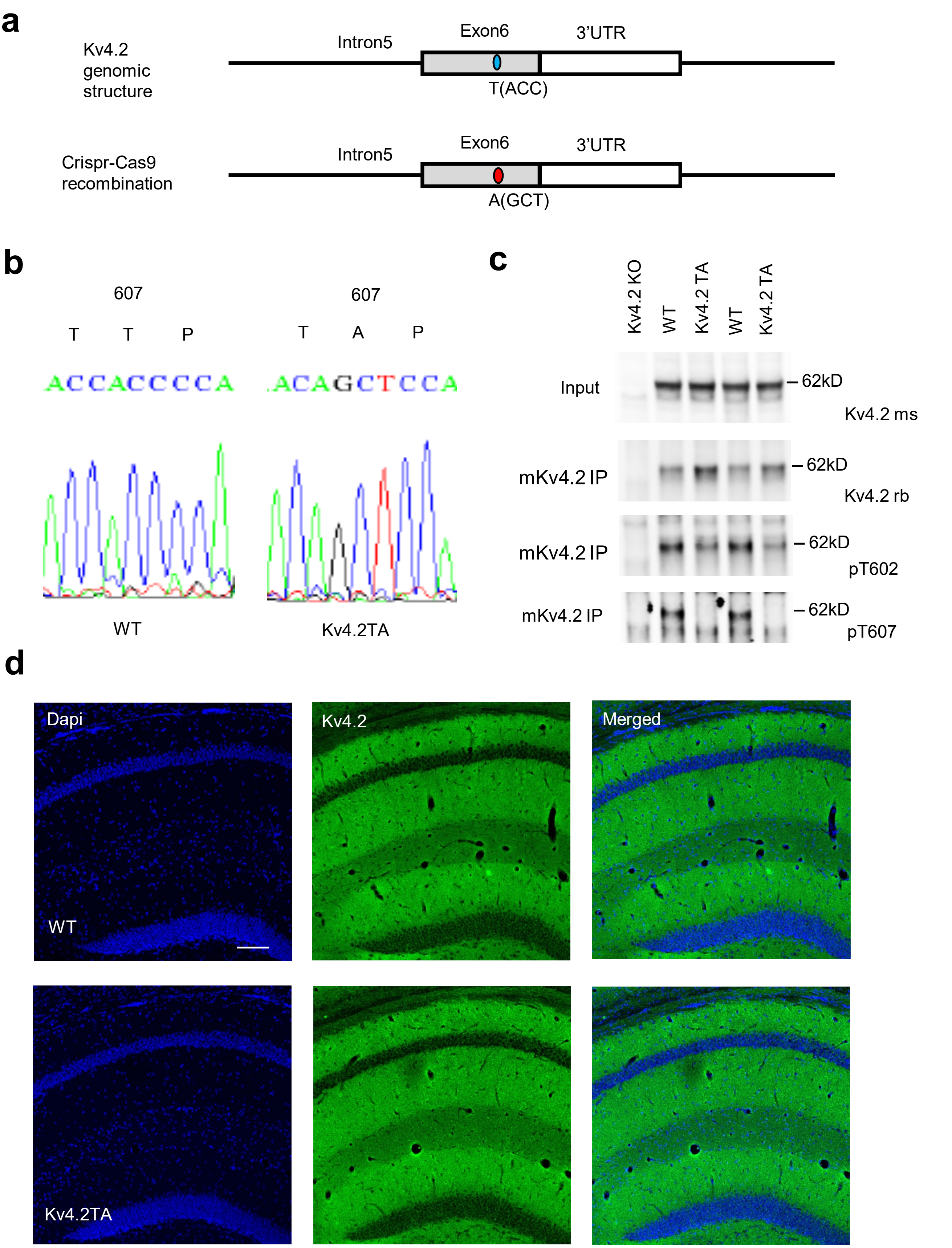

Supplementary Figure 6

Supplementary Figure 6. Kv4.2TA mouse generation.

a, Strategy of Kv4.2 T607A mutant mouse (Kv4.2TA) generation. The mutation was introduced with the Crispr-Cas9 technique. **b,** Genomic DNA was extracted and PCR products were purified for sequencing. Sequencing data verified the mutations were produced as intended. **c,** Brain lysates from WT and Kv4.2TA mice were incubated with Kv4.2 antibody. IP samples were blotted with pT602 and pT607 antibodies. Phosphorylation of Kv4.2 T607 was disrupted in Kv4.2TA mice. The pT602 level is reduced in Kv4.2TA mice, probably because of the decrease of pT602 antibody recognition or pT607's impact on pT602. Data was repeated in three independent experiments. **d,** Immunostaining of Kv4.2 in WT and Kv4.2TA brain showing the Kv4.2 expression pattern was not altered in Kv4.2TA hippocampus. Scale bars: 100 μm. Data was repeated in four independent experiments.

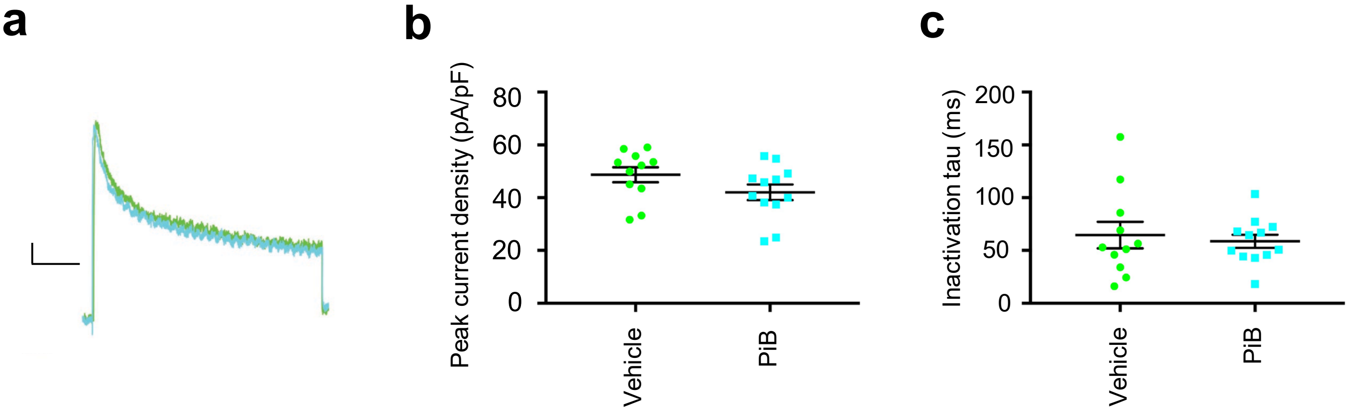

Supplementary Figure 7

Supplementary Figure 7. Pin1 inhibition with PiB has no effect on A-current in Kv4.2TA mice.

a, Trace of transient I_A in response to PiB (4 μM) treatment in Kv4.2TA. Inactivating I_A was isolated by subtracting total K^+ measured from a step to +40 mV from a -120 mV pre-pulse from a subsequent step to +40 mV from -30 mV. Scale: 10 pA / 100 ms. **b,** I_A density in outside-out patches is not affected by PiB treatment in Kv4.2TA mice (n = 11 for vehicle, 12 for PiB), unpaired two-tailedT-test, $p > 0.05$. **c,** No significant difference in decay kinetics was observed in response to the treatment and vehicle control, unpaired two-tailedT-test, $p > 0.05$. Data are presented as mean ± SEM.

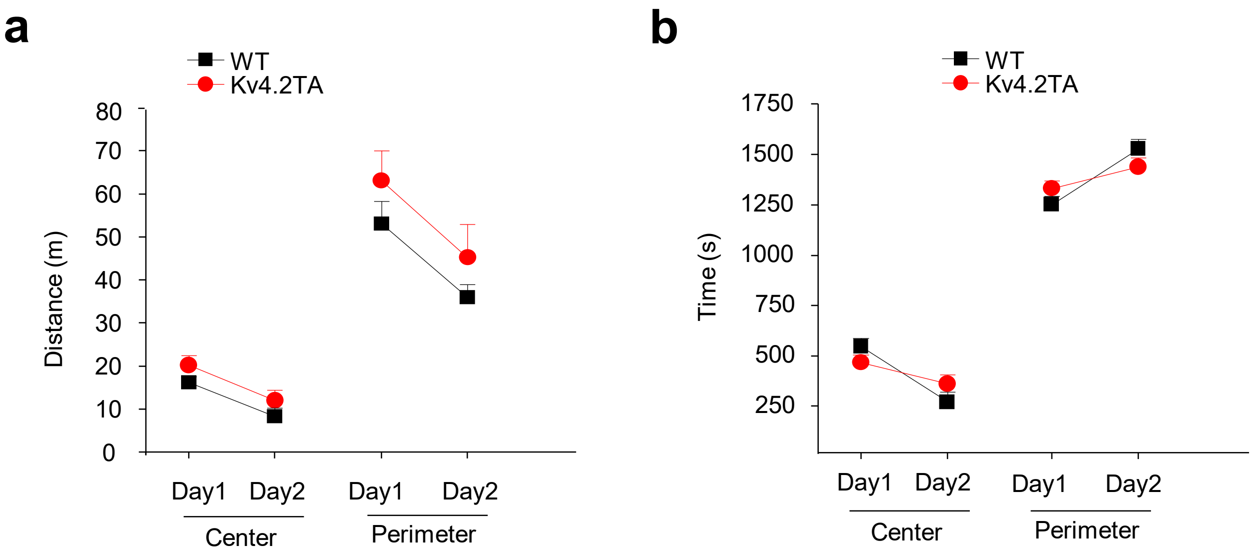

Supplementary Figure 8

Supplementary Figure 8. Kv4.2TA mice showed normal motor activity in open field test.

a, Total distance traveled in various regions of the open field apparatus by WT and Kv4.2TA mice. The mice were tested for 30 min sessions on two consecutive days. **b,** Time spent in the center or perimeter of the open field apparatus. n = 12 for WT, 16 for Kv4.2TA. Data are presented as mean ± SEM.

Parameter	WT (mean ± SEM; n)	Kv4.2TA (mean ±SEM; n)
Rise time (ms)	11.69 ±.74; n=14	11.27 ±1.06; n=15
Half-activation (V_H) (mV)	24.76 ±3.47; n=12	34.74 ±4.2; n=11
Half-activation (k) (mV)	18.74 ±74; n=12	19.94 ±1.14; n=11
Half-inactivation (V_H)(mv)	-61.77 ±1.57; n=13	-64.55 ±2.42; n=9
Half-inactivation (k) (mV)	4.60 ±1.00; n=13	5.97 ±0.83; n=9

Supplementary Table 1. Outside-out somatic patch recordings of A-current. Inactivation kinetics, rise time and voltage dependence of activation and inactivation.

Fig. 1a

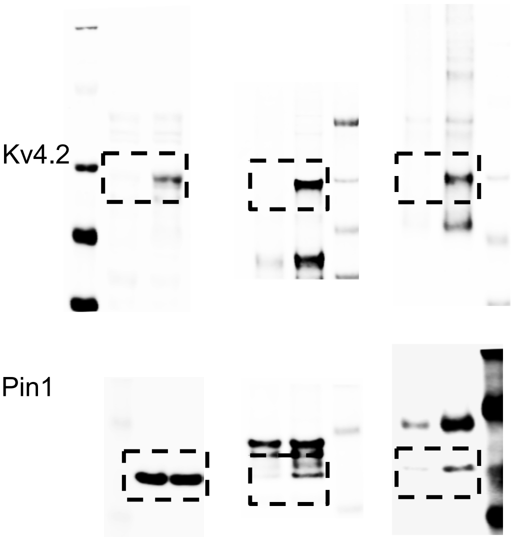

Fig. 1c

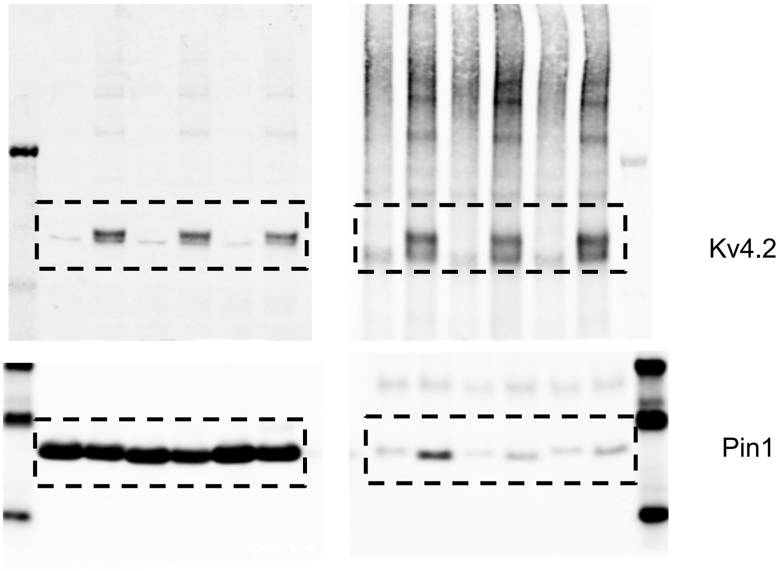

Fig. 1e

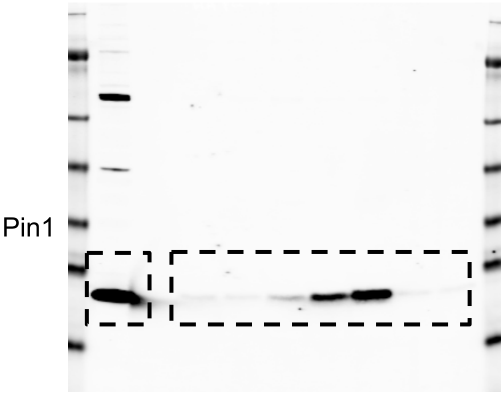

Fig. 1f

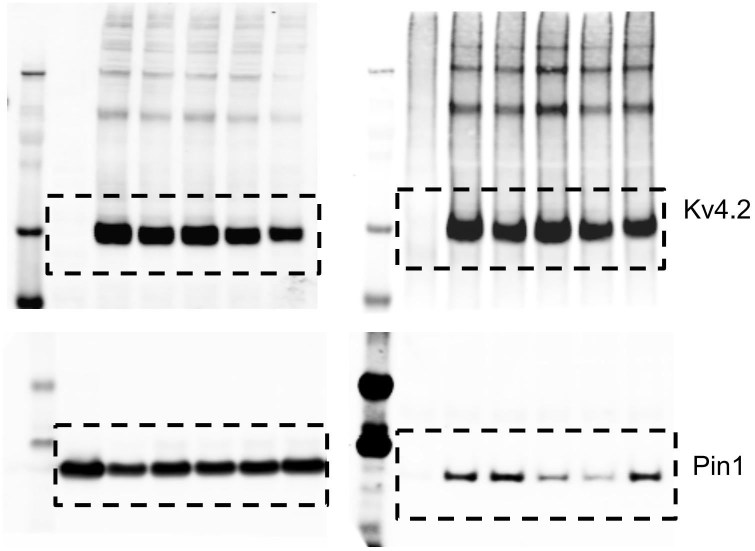

Fig. 1j

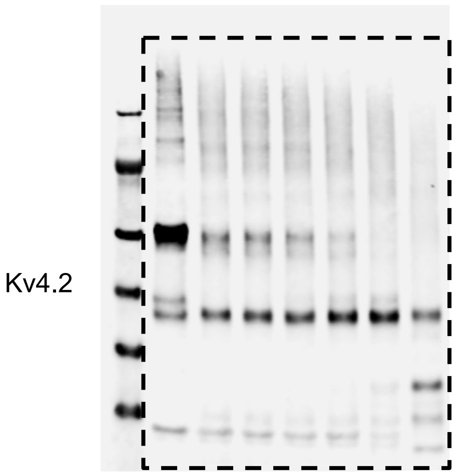

Fig. 1k

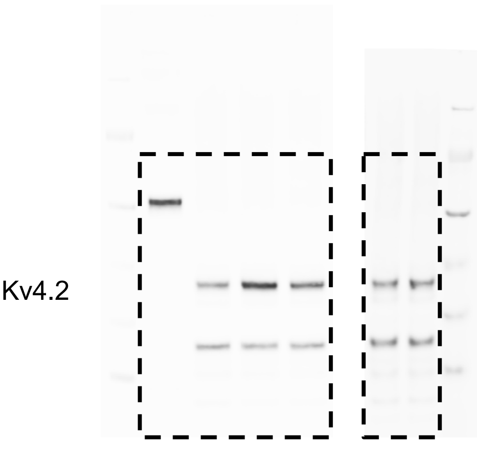

Fig. 2a

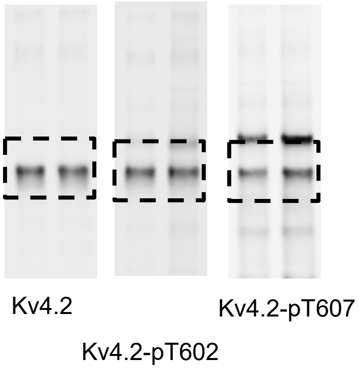

Fig. 2b

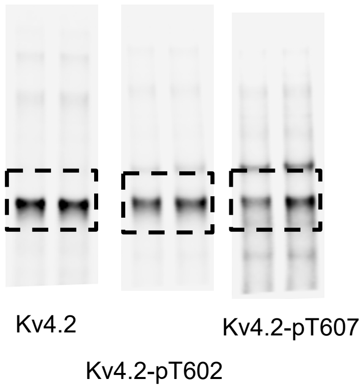

Fig. 2c

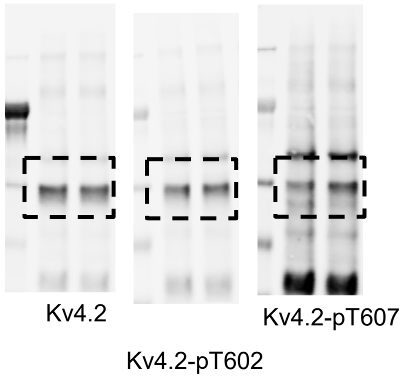

Fig. 2d

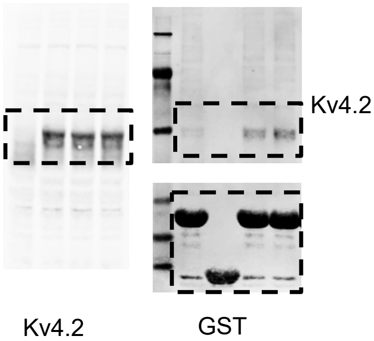

Fig. 2e

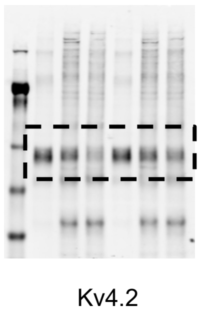

Fig. 2f

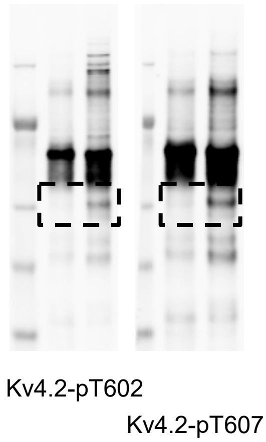

Fig. 2g

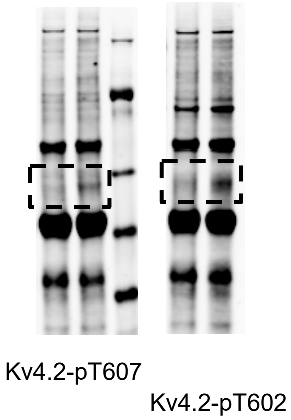

Fig. 3a

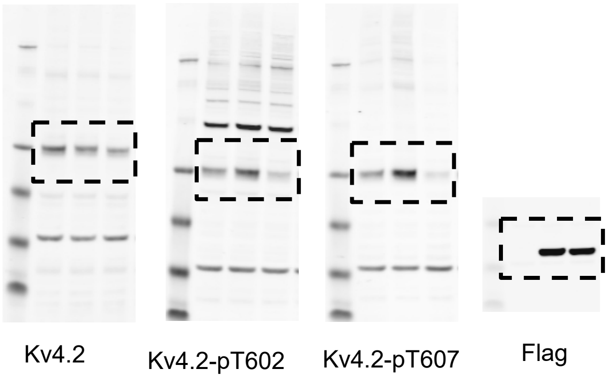

Fig. 3b

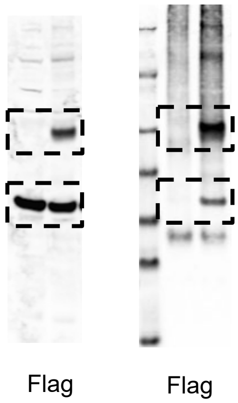

Fig. 3c

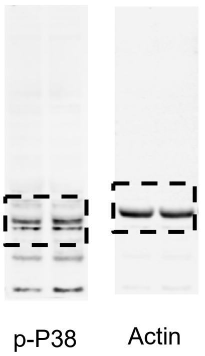

Fig. 3d

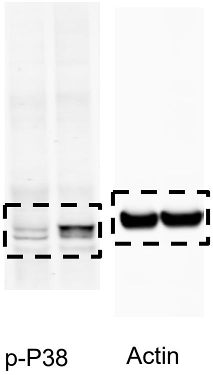

Fig. 3e

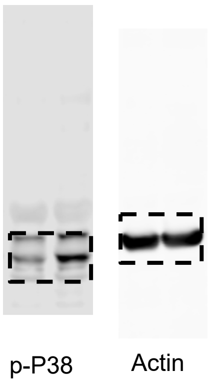

Fig. 3f

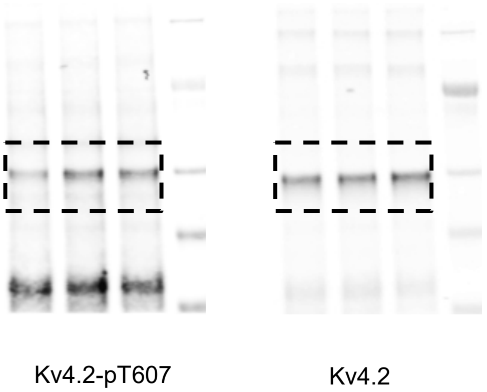

Fig. 3g

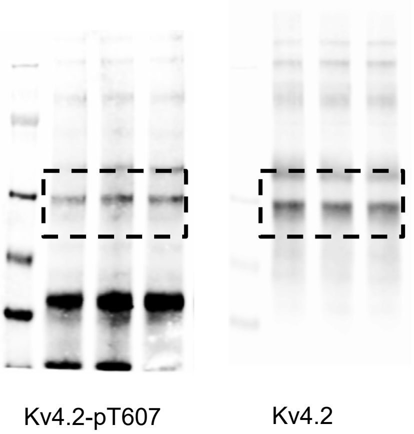

Fig. 4a

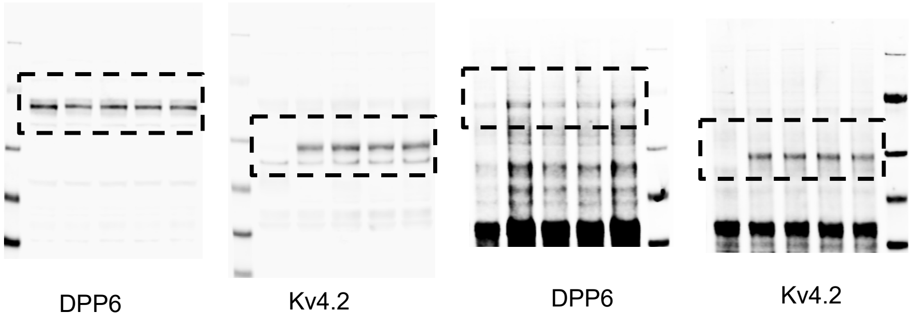

Fig. 4b

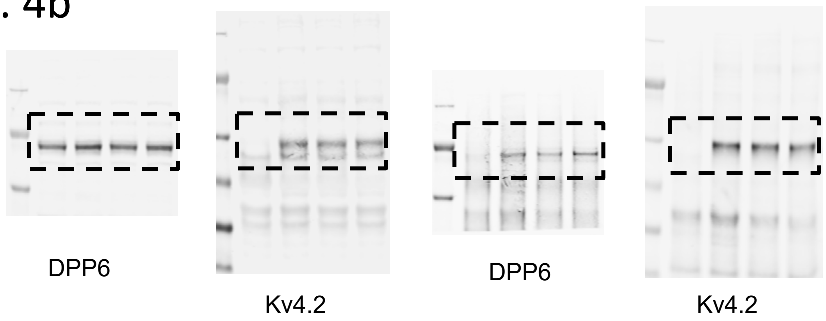

Fig. 4c

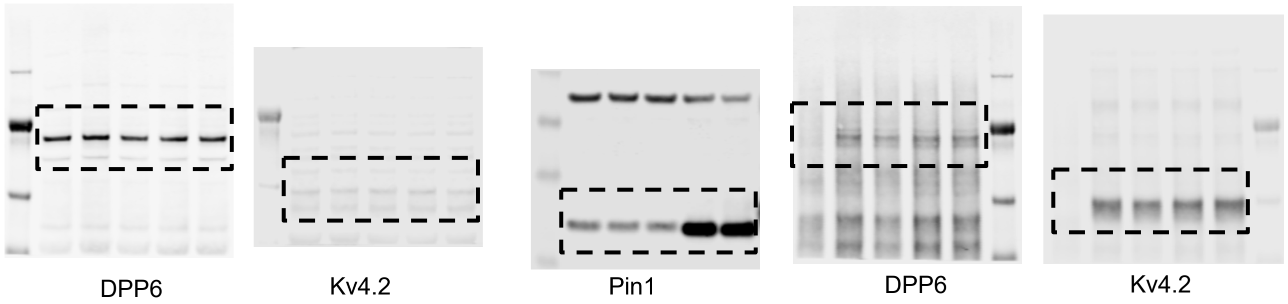

Fig. 4d

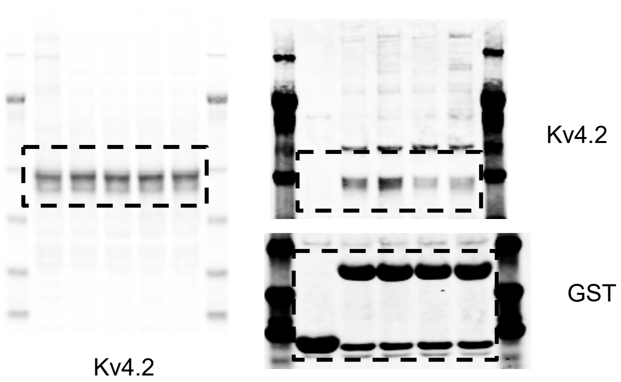

Fig. 4e

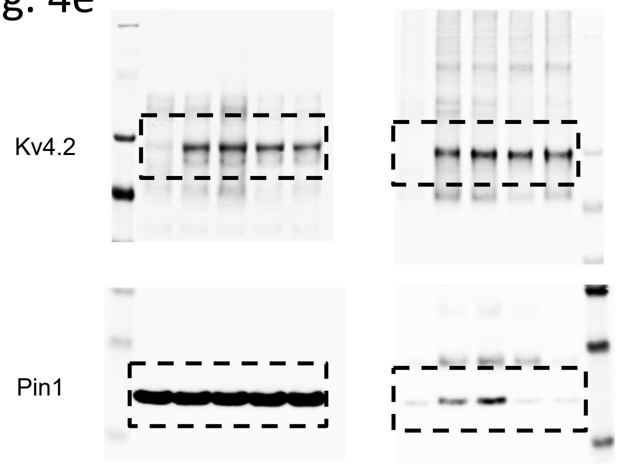

Fig. 4F

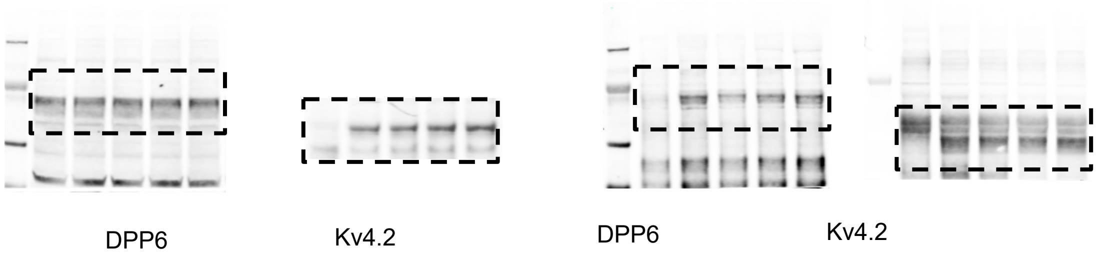

Fig. 5g

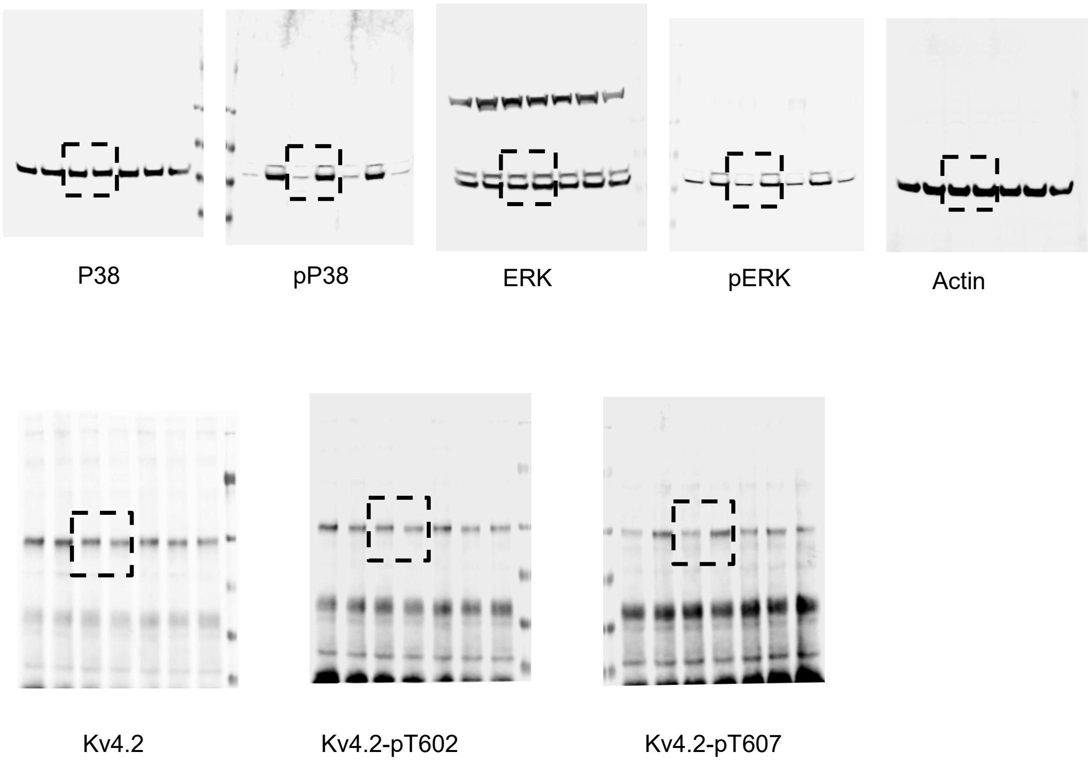

Supplementary Fig. 2a

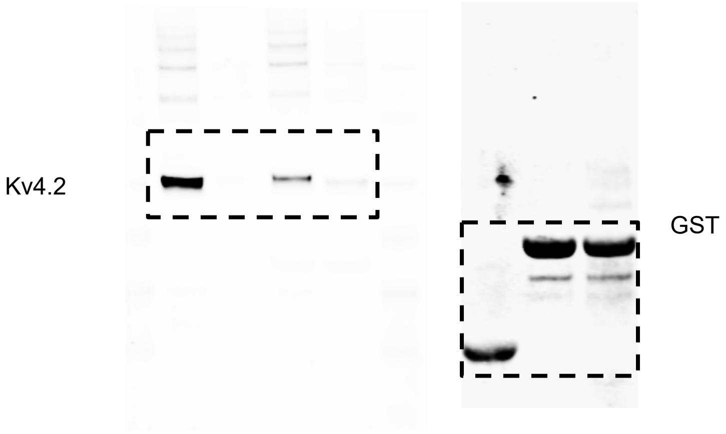

Supplementary Fig. 2b

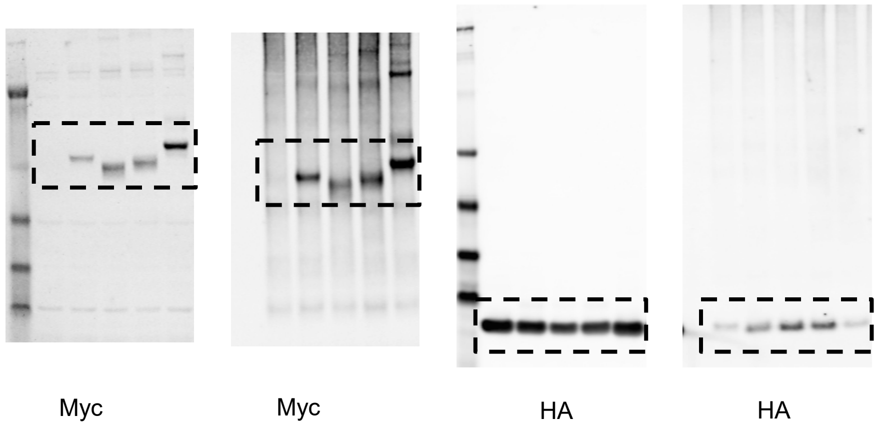

Supplementary Fig. 3b

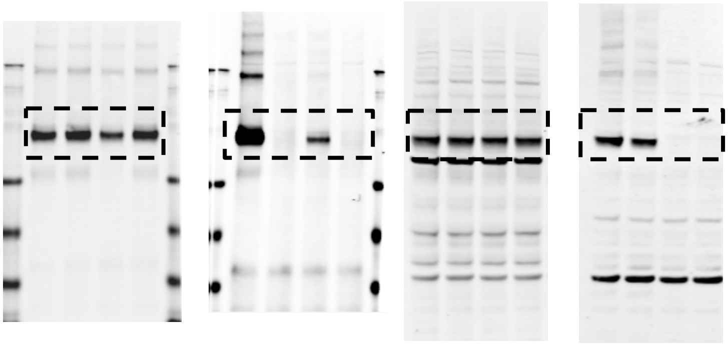

Kv4.2 Kv4.2-pT602 Kv4.2 Kv4.2-pT607

Supplementary Fig. 3c

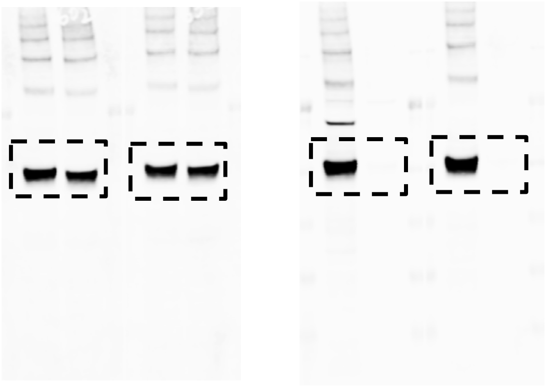

Kv4.2 Kv4.2-pT602 Kv4.2-pT607

Supplementary Fig. 4a

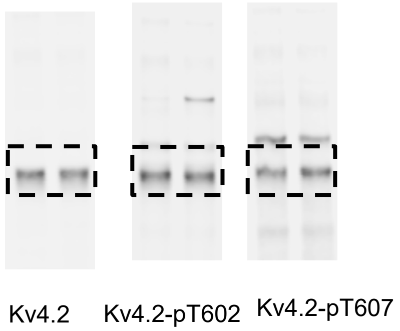

Supplementary Fig. 4b

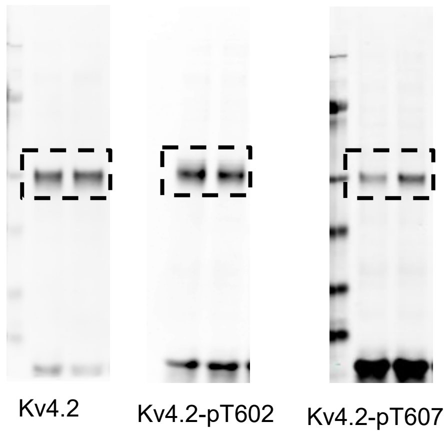

Supplementary Fig. 5b

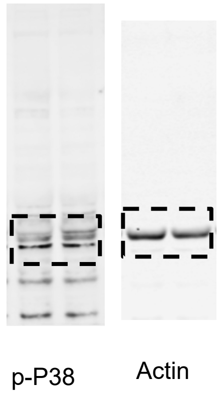

Supplementary Fig. 6c

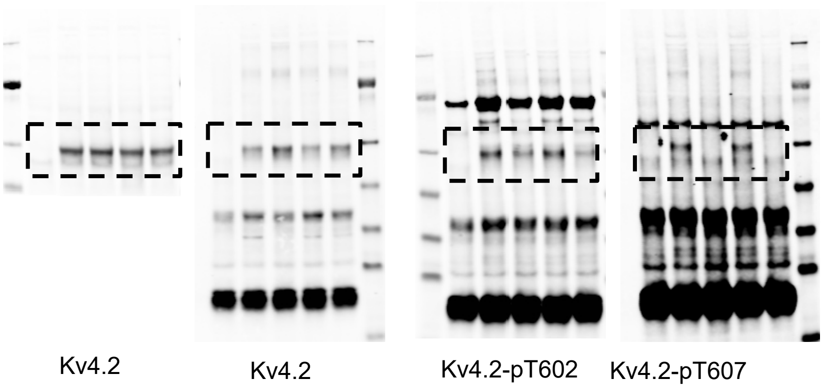

Supplement: Supplementary file 1 — Supplementary Information [file 41467_2020_15390_MOESM1_ESM.pdf]
