# Supplementary material for: Activity-dependent isomerization of Kv4.2 by Pin1 regulates cognitive flexibility
Source: Nat Commun. 2020 Mar 26;11:1567. doi: 10.1038/s41467-020-15390-x (PMC7099064; doi:10.1038/s41467-020-15390-x)
Supplement: Supplementary file 3 — Reporting summary [file 41467_2020_15390_MOESM3_ESM.pdf]

nature research

Corresponding author(s): Dax A. Hoffman

Last updated by author(s): 2/7/2020

Reporting Summary

Nature Research wishes to improve the reproducibility of the work that we publish. This form provides structure for consistency and transparency in reporting. For further information on Nature Research policies, see Authors & Referees and the Editorial Policy Checklist .

Statistics

For all statistical analyses, confirm that the following items are present in the figure legend, table legend, main text, or Methods section.

n/a	Confirmed	
☐	☒	The exact sample size (*n*) for each experimental group/condition, given as a discrete number and unit of measurement
☐	☒	A statement on whether measurements were taken from distinct samples or whether the same sample was measured repeatedly
☐	☒	The statistical test(s) used AND whether they are one- or two-sided<br>*Only common tests should be described solely by name; describe more complex techniques in the Methods section.*
☒	☐	A description of all covariates tested
☐	☒	A description of any assumptions or corrections, such as tests of normality and adjustment for multiple comparisons
☐	☒	A full description of the statistical parameters including central tendency (e.g. means) or other basic estimates (e.g. regression coefficient) AND variation (e.g. standard deviation) or associated estimates of uncertainty (e.g. confidence intervals)
☐	☒	For null hypothesis testing, the test statistic (e.g. *F*, *t*, *r*) with confidence intervals, effect sizes, degrees of freedom and *P* value noted<br>*Give P values as exact values whenever suitable.*
☒	☐	For Bayesian analysis, information on the choice of priors and Markov chain Monte Carlo settings
☒	☐	For hierarchical and complex designs, identification of the appropriate level for tests and full reporting of outcomes
☒	☐	Estimates of effect sizes (e.g. Cohen's *d*, Pearson's *r*), indicating how they were calculated

Our web collection on statistics for biologists contains articles on many of the points above.

Software and code

Policy information about availability of computer code

Data collection	Clampex 10.7, Anymaze 5.1, Zen2.3,
Data analysis	Origin 2018b, Statistica 9.0, Graphpad Prism 7.0, Clampfit 10.7, Image J 1.52a, CHARMM, UCSF Chimera, MATLAB R2018a

For manuscripts utilizing custom algorithms or software that are central to the research but not yet described in published literature, software must be made available to editors/reviewers. We strongly encourage code deposition in a community repository (e.g. GitHub). See the Nature Research guidelines for submitting code & software for further information.

Data

Policy information about availability of data

All manuscripts must include a data availability statement. This statement should provide the following information, where applicable:

- Accession codes, unique identifiers, or web links for publicly available datasets
- A list of figures that have associated raw data
- A description of any restrictions on data availability

PDB:2N10, PDB:2Q5A, and other data that support the findings of this study are available from the corresponding authors in reasonable request.

Field-specific reporting

Please select the one below that is the best fit for your research. If you are not sure, read the appropriate sections before making your selection.

☒ Life sciences ☐ Behavioural & social sciences ☐ Ecological, evolutionary & environmental sciences

For a reference copy of the document with all sections, see nature.com/documents/nr-reporting-summary-flat.pdf

Life sciences study design

All studies must disclose on these points even when the disclosure is negative.

Sample size	No statistical methods were used to predetermine sample sizes, but our samples are similar to or exceed those that are generally employed in the field.
Data exclusions	No data were excluded for statistical analyses.
Replication	All attempts at replication were successful. At least two experiments were performed independently as noted in the paper.
Randomization	The mice were randomly grouped into control and PTZ / KA treatment.
Blinding	For behavioral studies and measures of IA, investigators were binded to genotypes. All other cases were initial characterizations for which there was not a specific prediction.

Reporting for specific materials, systems and methods

We require information from authors about some types of materials, experimental systems and methods used in many studies. Here, indicate whether each material, system or method listed is relevant to your study. If you are not sure if a list item applies to your research, read the appropriate section before selecting a response.

Materials & experimental systems

n/a	Involved in the study	
☐	☒	Antibodies
☐	☒	Eukaryotic cell lines
☒	☐	Palaeontology
☐	☒	Animals and other organisms
☒	☐	Human research participants
☒	☐	Clinical data

Methods

n/a	Involved in the study	
☒	☐	ChIP-seq
☒	☐	Flow cytometry
☒	☐	MRI-based neuroimaging

Antibodies

Antibodies used	Mouse anti-Kv4.2 (NeuroMab, 75-016) was used at 1:2000 for western blot, 1:200 for immunostaining, Rabbit anti-Kv4.2 (Sigma, P0233) was used at 1:2000 for western blot, rabbit anti-Kv4.2 (Sigma, HPA029068) was used at 1:200 for staining, pT602 (Santa Cruz, SC-16983-R) was used at 1:1000 for western blot, pT607 (Santa Cruz, SC-22254-R) was used at 1:500 for western blot, Pin1 (Santa Cruz, SC-46660) was used at 1:100 for staining, Pin1 (Millipore, 07-091) was used at 1:3000 for western blot, p38 (Cell Signaling, 9212s) was used at 1:1000 for western blot, p-p38 (Cell Signaling, 4511s) at 1:1000 for western blot, DPP6 (Abcam, 41811) was used at 1:2000 for western blot, Myc (Millipore, 05-419) was used at 1:10000 for western blot, HA(Santa Cruz, SC-805) was used at 1:1000 for western blot, Actin (Sigma, A-1978) was used at 1:10000 for western blot; Alexa Fluor 488 goat anti-mouse (Invitrogen, A-11029) was used at 1:500; Alexa Fluor 488 goat anti-rabbit (Invitrogen, A-11034) was used at 1:500; Alexa Fluor 555 goat anti-mouse (Invitrogen, A-21424) was used at 1:500; Alexa Fluor 555 goat anti-rabbit (Invitrogen, A-21429) was used at 1:500; Alexa Fluor 680 goat anti-mouse (Invitrogen, A-21057) was used at 1:10000; Alexa Fluor 680 goat anti-rabbit (Invitrogen, A-21076) was used at 1:10000; IRDye 800CW goat anti-mouse (Licor, 926-32210) was used at 1:10000, IRDye 800CW goat anti-rabbit (Licor, 926-32211) was used at 1:10000.
Validation	Antibody validation was deferred to the manufacturers and was supported by multiple publications per statements on the manufacturer's website.

Eukaryotic cell lines

Policy information about cell lines

Cell line source(s)	HEK293T cell lines were originally purchased from ATCC.
Authentication	Cell lines were used without further authentication.
Mycoplasma contamination	Cell lines were not tested for mycoplasma contamination.
Commonly misidentified lines (See ICLAC register)	No commonly misidentified cell lines were used in this study.

Animals and other organisms

Policy information about studies involving animals; ARRIVE guidelines recommended for reporting animal research

Laboratory animals	Adult male C57BL6, Kv4.2TA and WT littermates, and B6D2F1 mice were used in this study.
Wild animals	The study did not involve wild animals.
Field-collected samples	All the behavioral experiments were performed in the behavior room located in the animal facility. No field-collected samples were used in the study.
Ethics oversight	All research involving animals was complied with protocols approved by the National Institute of Child Health and Human Development Animal Care and Use Committee and in accordance with NIH guidelines.

Note that full information on the approval of the study protocol must also be provided in the manuscript.
