# Supplementary material for: Activity-dependent isomerization of Kv4.2 by Pin1 regulates cognitive flexibility
Source: Nat Commun. 2020 Mar 26;11:1567. doi: 10.1038/s41467-020-15390-x (PMC7099064; doi:10.1038/s41467-020-15390-x)
Supplement: Supplementary file 2 — Peer Review [file 41467_2020_15390_MOESM2_ESM.pdf]

Reviewers' comments:

Reviewer #1 (Remarks to the Author):

Review Hu et al. “Activity-dependent isomerization of Kv4.2 by Pin1 regulates cognitive flexibility”

Overall the authors have implemented a wide range of techniques to provide an impressive amount of data on the proposed role for a p38-mediated phosphorylation that promotes Pin1 association with the C terminus of Kv4.2 at T607 and T602, a resulting isomerization, and final DPP6 dissociation from the Kv4.2 complex. This cascade of events could be triggered by a novel environment or either PTZ or KA induced seizures. They use HEK 293 cells, brain lysates and both cell cultures and in vitro slices of hippocampus to conduct a large series of protein biochemical and coIP tests and recordings to test different aspects of this cascade using blockers against p38, Pin1, and a CRISPR-generated mutant mouse with the phosphorylation sites mutated. A final set of behavioral tests documents a selective change in reversal learning in the water maze and lever pressing tests. In general the work is conducted very carefully in providing necessary controls in the form of the mutant Kv4.1TA mouse and complementary pharmacological tests and blockers (see below). Western blots are further quantified which adds a great deal of faith in the conclusions drawn from the blots presented. In the end I am convinced they have identified a new pathway by which p38-mediated phosphorylation of Kv4.2 channels can lead to dissociation of DPP6 and the attendant predicted changes in channel density and kinetic properties (see below. Key to these conclusions is that there is a basal level of phosphorylation of Kv4.2 that can be up or downregulated by different stimuli that include seizure activity (PTZ or KA-induced) an enriched environment, and specific forms of behavioral testing.Overall an impressive body of work.
However, there are key issues that need to be resolved, and in places the text and figure legends improved to make the work more understandable. My main criticisms and suggestions are listed below:

Main Issues:
- p38 vs ERK phosphorylation: While many of the tests conducted here included necessary controls there is a fundamental issue that was left out that needs to be addressed. The authors identify three sites on the C terminus for potential p38 phosphorylation that happen to also coincide with the established sites for ERK-mediated phosphorylation (Schrader et al., 2006). Unfortunately the potential for ERK to account for at least some of the results presented here were dismissed very early on on the basis of the relative level of banding on Western blots in HEK cells for ERK and a constitutively active form of ERK compared to expression of p38 (Figs. 3a, S4). It also not adequate to use the relative banding by p38 (Fig. 3a) vs ERK (Fig S4) to dismiss the potential role of ERK, which has also been shown to be activated by both KA- and PTZ-induced seizures (Kim et al., 1994; Zhu et al., 2017). I am thus not convinced that all of the results can be attributed to only p38 since no ERK blockers were ever applied. The authors need to repeat the tests in the presence of ERK blockers in (at least) Fig. 3f, Fig. 4, all of Fig 5, and infusion in vivo prior to behavioral tests to validate the extent to which the proposed p38 pathway can account for these results. The Kv4 current recordings should also be repeated for the ERK tests in Supp. Fig 4 along with protein biochemical tests of whether DPP6 dissociates in a similar fashion when this kinase is active and the p38 pathway is blocked.

DDP6 and Kv4.2 properties: There is one figure (Fig 4) where the association of DPP6 with Kv4.2 is shown to be reduced if the p38-Pin1 pathway is interrupted or prevented in tests with the Kv4.2TA knockin mutant mouse line. This is the basis for later interpretations of physiological tests using a solid battery of p38 and Pin1 blockers in Fig. 5 (how fast are these effects?). However, it has been shown that the effects of ERK-mediated phosphorylation on Kv4 channel properties is actually dependent on KChIPs that represent another important component of the Kv4 complex (Schrader et al., 2006). This needs to be considered and the results interpreted in light of this if KChIPs were not coexpressed in all the protein biochemical tests (or do these cells endogenously express KChIPs?). Could the effects of p38-mediated phosphorylation also be KChIP-dependent?

Secondly, there are no actual stimuli applied to invoke the changes in spike firing or Kv4 properties presented in Fig 5. If this supports the presence of a basal level of Pin1 activity this should be discussed. But I would have expected some number of these tests to have been conducted on slices from PTZ or KA treated hippocampus to be in line with the rest of the protein biochemical tests, with the advantage of applying some of the blockers they have available. In a similar manner, while all the final tests on Kv4 current are valuable and central to the interpretations applied throughout all of the tests are essentially on preventing the p38-Pin1-DPP6 cascade from being active. While the interpretations of a higher basal level of DPP6 association with the Kv4.2 population is consistent with the data, the study needs to provide a straightforward test to activate this pathway to confirm the proposed effects on Kv4 current as presented in Fig 7. The results shown for AMPA in Fig 4b would appear to provide a means to do this as could PTZ or KA pretreatment as in the rest of the study. Since this lab has made use of DPP6 KO animals in the past I was surprised not to see some direct tests on Kv4 current from these animals to compare to channels that have lost DPP6 in the manner proposed here. In the study of LinLin (2018) from this lab it is stated that DPP6 KO mice have altered water maze testing results. How do those compare to the dynamic loss of DPP6 and water maze results here? Use of shRNA to knock down DPP6 for Kv4 current recordings in hippocampal cultures? I would also like to see the effects of Pin1 blockers on Kv4 current recorded in wt vs Kv4.2TA in Fig. 5h. In the Discussion a section should be added to address the recorded effects on Kv4 activation and inactivation properties as these are affected by DPP6 expression but did not apparently change between wt and Kv4.2TA cells (Suppl Table).

Minor Comments:
- While the protein biochemical tests in the majority of figures are backed up by quantification Fig 1 stands out in not having that analysis on blots that are not as convincing as later tests.
- The manuscript suffers from a difficult writing style that often fails to introduce new terms or molecular paths sufficiently to allow readers to understand what is being stated. There is sometimes a mismatch in the conclusions being drawn between the main text and the Figure Legends, which themselves are not adequate to describe what is being shown. This is particularly the case in Fig 1 where the text correctly refers to a dramatic reduction in band intensity and the legend used the term "abolished" throughout. Fig 1 legend one also stands out as one of the weakest in terms of labeling and information available in the legend itself.
- Introduction - A large portion of the Introduction deals with the published effects of Kv4.2 KOs on LTD and LTP but these were ultimately not tested in the current study. Would recommend focusing on the known roles of Kv4.2 KOs on firing properties [[or other aspects of Kv4 function]] actually tested here.
- Figure 2 - The legend states that PTZ seizures do NOT induce phosphorylation of pT602 and yet the bar plot gives it a symbol of significance (Fig. 2c).
- Fig 3c - no Y axis label is present
- Figure 5 - no indication is made in the legend for Fig. 5g as to what is being compared in the two representative traces. I assume it is wt and Kv4.2TA but it is not indicated.

Kim YS, Hong KS, Seong YS, Park JB, Kuroda S, Kishi K, Kaibuchi K, Takai Y (1994) Phosphorylation and activation of mitogen-activated protein kinase by kainic acid-induced seizure in rat hippocampus. Biochem Biophys Res Commun 202:1163–1168.

Schrader LA, Birnbaum SG, Nadin BM, Ren Y, Bui D, Anderson AE, David Sweatt J (2006) ERK/MAPK regulates the Kv4.2 potassium channel by direct phosphorylation of the pore-forming subunit. American Journal of Physiology-Cell Physiology 290:C852–C861

Zhu X, Dong J, Han B, Huang R, Zhang A, Xia Z, Chang H, Chao J, Yao H (2017) Neuronal Nitric Oxide Synthase Contributes to PTZ Kindling Epilepsy-Induced Hippocampal Endoplasmic Reticulum Stress and Oxidative Damage. Front Cell Neurosci 11:377.

Reviewer #2 (Remarks to the Author):

Hu et al. present a novel potential mechanism by which the isomerase Pin1 may regulate the assembly of Kv4.2 with DPP6 and thereby the activity of Kv4.2 channels which impacts neuronal excitability and behaviour. Starting with the observation of Pin1 binding to Kv4.2 in a heterologous screening experiment, they further characterized the putative Pin1 binding motif(s) and found increased phosphorylation of Kv4.2 at position T607 and reduced interaction with DPP6 in paradigms of neuronal stimulation. p38 MAP kinase increased phosphorylation of T607 in a model peptide which enhanced binding to Pin1. The authors finally generated a Kv4.2(T607) mouse model to confirm these findings and found an effect of this mutation on reversal learning interpreted in line with their proposed mechanism.

Despite the employment of a multitude of techniques, the basic hypothesis and key findings are based on heterologous/recombinant experiments with poor quality antibody readout. In fact, IP-verified antibodies, Kv4.2 knockout mouse control and mass spectrometry are all available to perform sensitive, quantitative and stringently controlled state of the art biochemical experiments. Furthermore, important data and experimental details are missing (in particular on mass spectrometry, generation/verification of antibody tools and recombinant constructs).
I have substantial doubts about the validity of the basic hypothesis , interpretation and quality of the data and therefore can not recommend publication of this work.

To mention the main concerns / shortcomings:

- AP-MS analyses of native Kv4.2 have been carried out before with at least reasonable quality (e.g. Marionneau et al.) which offered a quite comprehensive view on the Kv4.2 interactome. This work should be acknowledged and discussed. It represents an experimental standard to be met.

- The authors fail to convincingly show that Pin1 binds to endogeneous Kv4.2 under native conditions (Fig. 1b shows a low quality Westernblot with a weak Pin1 signal that is also present in the KO control eluate). Modern mass spectrometry is more sensitive and quantitative than Westernblot and should be used as a readout for this experiment.

- The authors mention MS results from initial pulldowns from HEK cells and neuronal cultures but do not present any of that data. Furthermore, these experiments lack adequate controls and are - by their nature (overexpression, culture systems) – prone to artifacts. Explicitly, IgG or, worse, „empty" beads are insufficient controls for any affinity-based biochemical experiments.

- No compelling evidence is presented to verify the specificity of the polyclonal phospho-specific antibodies that were used throughout the paper for phosphorylated versus non-phosphorylated Kv4.2 wt or mutant protein. The experiment in S2 b shows Westernblot analysis of heterologously expressed Kv4.2 wt and point mutants. The phosphorylation status of these Kv4.2 proteins is not known, so it is not clear whether these antibodies are strictly phospho- or sequence-specific (or a mixture of both).

- Mass spectrometric identification of Kv4.2 phosphorylation sites should be at least confirmed with endogeneous Kv4.2 protein, and the respective pairs of annotated MS/MS spectra (i.e. of the peptides in phosphorylated and non-phosphorylated state) must be shown. Information on the search database, search algorithm and search parameters must be given.

- Throughout the manuscript, the significance of Westernblot signals is heavily overrated. Density-based quantification of Westernblot signals is not accurate enough to reliably quantify the relatively small changes claimed. No information is given on how Westernblots were actually performed, recorded and quantitatively evaluated.

- The effects of the Kv4.2 phosphosite mutations on Pin1-association (Fig. 1f) and DPP6 binding (Fig. 4a, b, d) seem to be much smaller than the difference in binding of the respective phosphopeptides to Pin1 (Fig. 1e). Apparently, binding is determined not only by phosphorylation but also by the primary sequence and the structural context. This has to be taken into account when interpreting the results from the Kv4.2(T607A) knockin model.

- As a general remark, stable binding of an isomerase to a protein is not expected if the protein is a substrate. The same applies to protein kinases. It should be verified by structural analysis that (phosphorylated) Kv4.2 is a real substrate of Pin1 (i.e. undergoes the expected structural change catalyzed by Pin1).

Reviewer #3 (Remarks to the Author):

KV4.2 is the primary A-type K channel and regulates neuronal excitability, which is implicated in synaptic plasticity, behaviors, and disorders. Hu et al. showed here that Kv4.2 interacts with Pin1 through the phosphorylation of KV4.2 by ERK p38. The KV4.2 phosphorylation level is altered in mouse with the enriched environment or seizure-inducing drugs. Furthermore, the KV4.2 phosphorylation regulates KV4.2 and DPP6 interaction. Finally, the knockin mice carrying the non-phospho mimic mutation in KV4.2 showed altered excitability and behaviors. From these results, the authors conclude the role of the macromolecular KV4.2 complex in regulating brain function. Overall, a significant amount of results were shown, and the proposed regulation of the KV4.2 complex is novel, but its conclusion is not fully supported.

Major
1, Protein complex
The authors proposed that Pin1 interaction with KV4.2 dissociates DPP6 from KV4.2 based on their data showing each interaction. However, dynamic regulation of the tripartite complex of the Pin1/KV4.2/DPP6 in vivo has not been shown. In another word, does Pin1 interaction dissociates DPP6 from KV4.2 or does Pin1 interact better with KV4.2 alone? More importantly, it remains unclear how the cytoplasmic Pin1 affects DPP6 interaction with KV4.2 through their transmembrane domains. Since this is the main point, this should be clearly shown.

2, Characterization of novel KV4.2 knockin mice.
The authors produced novel KV4.2 TA knockin mice and provided several phenotypes. Notably, changes in the firing frequency strongly indicate the significance of this phosphorylation sites. However, the data requires some critical control. Fig 5a.b, the authors concluded that the reduction in the firing frequency by Pin1 inhibitor and rescue by AmmTX3 suggests both on a similar pathway. However, AmmTX3 depolarizes more independent from the Pin1 pathway. To clarify this, it is important to provide AMMTx3 alone in Fig 5b.

3, Connection between the biochemical and physiological phenotypes. The authors showed that DPP6 and KV4.2 form a complex similarly in WT and KV4.2 TA mice before the drug treatment (Fig 4d). On the other hand, their firing frequency is different (Fig 5a). Does this mean that repetitive stimulation causes DPP6 dissociation from KV4.2? The authors need to explain this further, and this may require additional experiments.

Minor
Table of MS analysis of the KV4.2 complex should be shown. Especially, because PIN1 is identified from HEK proteomics, but not neuron proteomics, and because the Pin1 Co-IP on Fig 1b is modest.

Requires control, phosphatase-treated samples, to show phosphorylation-dependent interaction.

Knockin mice. The knockin mice at T607 showed altered phosphorylation at T602 and altered IP efficiency. Please discuss.

Fig 4. The title indicates that the main point is phosphorylation-dependent interaction with the other proteins. It is also required to show a blot with the pT607 antibody.

Discussion. The effect size in activity-dependent interaction is relatively modest. It should be discussed why the effect size is modest despite a lovely model on Fig 7.

Statistics. Was the data normality tested?

We thank all the reviewers for their time and helpful suggestions. Below we address each point raised. In this revision we have added the following experiments:

Biochemistry:

1. Colocalization of Kv4.2 and Pin1 in culture hippocampal neurons (Fig. 1b)
2. Statistical analysis of Kv4.2 coIP with Pin1 mutant (Fig. 1c)
3. Statistical analysis of Pin1 pull down by Kv4.2 peptides (Fig. 1e)
4. Statistical analysis of Pin1 coIP with Kv4.2 mutants (Fig. 1f).
5. Kv4.2 structural rearrangements by Pin1 (Fig. 1j, k)
6. P38 inhibitor effect on Kv4.2 phosphorylation at T607 (Fig. 3g)
7. MEK inhibitor effect on PTZ-induced dissociation of Kv4.2 and DPP6 (Fig. 4a)
8. P38 inhibitor effect on PTZ-induced dissociation of Kv4.2 and DPP6 (Fig. 4a)
9. Lambda protein phosphatase effect on Kv4.2 and Pin1 binding (Supplementary Fig. 2a)
10. Lambda protein phosphatase effect on Kv4.2 pT602 and pT607 antibodies (Supplementary Fig. 3c)
11. Slicing effect on p38 phosphorylation and Kv4.2 phosphorylation (Supplementary Fig. 3c)
12. DPP6 effect on KV4.2 and Pin1 binding (attached for the reviewers)

Electrophysiology:

1. AmmTX3 alone, effect on firing in WT slice (Fig. 5a, b and Tab. 1)
2. PiB effect on IA in WT slice (Fig. 5g-i)
3. MEK inhibitor effect on IA in WT slice (Fig. 5g-i)
4. P38 inhibitor effect on IA in WT slice (Fig. 5g-i)
5. PiB effect on IA in Kv4.2TA slice (Supplementary Fig. 7a-c)

We hope our revisions have addressed all your concerns, and that you can agree that the paper is now appropriate for publication in *Nature Communications*.

Reviewer #1 (Remarks to the Author):

Review Hu et al. "Activity-dependent isomerization of Kv4.2 by Pin1 regulates cognitive flexibility"

Overall the authors have implemented a wide range of techniques to provide an impressive amount of data on the proposed role for a p38-mediated phosphorylation that promotes Pin1 association with the C terminus of Kv4.2 at T607 and T602, a resulting isomerization, and final DPP6 dissociation from the Kv4.2 complex. This cascade of events could be triggered by a novel environment or either PTZ or KA induced seizures. They use HEK 293 cells, brain lysates and both cell cultures and in vitro slices of hippocampus to conduct a large series of protein biochemical and coIP tests and recordings to test different aspects of this cascade using blockers against p38, Pin1, and a CRISPR-generated mutant mouse with the phosphorylation sites mutated. A final set of behavioral tests documents a selective

change in reversal learning in the water maze and lever pressing tests. In general the work is conducted very carefully in providing necessary controls in the form of the mutant Kv4.1TA mouse
and complementary pharmacological tests and blockers (see below). Western blots are further quantified which adds a great deal of faith in the conclusions drawn from the blots presented. In the end I am convinced they have identified a new pathway by which p38-mediated phosphorylation of Kv4.2 channels can lead to dissociation of DPP6 and the attendant predicted changes in channel density and kinetic properties (see below. Key to these conclusions is that there is a basal level of phosphorylation of Kv4.2 that can be up or downregulated by different stimuli that include seizure activity (PTZ or KA-induced) an enriched environment, and specific forms of behavioral testing.Overall an impressive body of work.
However, there are key issues that need to be resolved, and in places the text and figure legends improved to make the work more understandable. My main criticisms and suggestions are listed below:

Main Issues:
- p38 vs ERK phosphorylation: While many of the tests conducted here included necessary controls there is a fundamental issue that was left out that needs to be addressed. The authors identify three sites on the C terminus for potential p38 phosphorylation that happen to also coincide with the established sites for ERK-mediated phosphorylation (Schrader et al., 2006). Unfortunately the potential for ERK to account for at least some of the results presented here were dismissed very early on on the basis of the relative level of banding on Western blots in HEK cells for ERK and a constitutively active form of ERK compared to expression of p38 (Figs. 3a, S4). It also not adequate to use the relative banding by p38 (Fig. 3a) vs ERK (Fig S4) to dismiss the potential role of ERK, which has also been shown to be activated by both KA- and PTZ-induced seizures (Kim et al., 1994; Zhu et al., 2017). I am thus not convinced that all of the results can be attributed to only p38 since no ERK
blockers were ever applied. The authors need to repeat the tests in the presence of ERK blockers in (at least) Fig. 3f, Fig. 4, all of Fig 5, and infusion in vivo prior to behavioral tests to validate the extent to which the proposed p38 pathway can account for these results. The Kv4 current recordings should also be repeated for the ERK tests in Supp. Fig 4 along with protein biochemical tests of whether DPP6 dissociates in a similar fashion when this kinase is active and the p38 pathway is blocked.

Response: We thank the reviewer for their introductory comments and agree with the reviewer that ERK blocker experiments would strengthen our findings on p38-dependent phosphorylation of Kv4.2 that primes the Pin1’s role in neuronal excitability. We have performed several

biochemical experiments and additional recordings using p38 inhibitor and MEK inhibitors and these are now in Figure 3g, 4a, and 5g-i. The findings show that MEK inhibitor SL327 does not block PTZ-induced phosphorylation of Kv4.2 at T607 (Fig. 3g). Furthermore, p38 inhibitor SB203580 (20 mg/kg, i.p.) blocks PTZ-induced Kv4.2-DPP6 dissociation while MEK inhibitor SL327 (30 mg/kg, i.p.) does not (Fig. 4a). In recordings, we showed that p38 inhibitor SB203580 (10 μM) significantly increases A-type current while MEK inhibitor PD98059 (20 μM) does not (Fig. 5g, 5h).

Many synaptic proteins are substrates of p38 and ERK MAPK. Therefore, the predictions of behavioral endpoints will be beyond Kv4.2-Pin1 pathway if the p38 and MEK inhibitors are administrated into mice. The reviewer also wants to see the effects of p38 and MEK inhibitors in HEK293T cells as we showed p38 and ERK expression increase Kv4.2 phosphorylation at T602 and T607. We anticipate that Kv4.2-DPP6 complex may not be similarly regulated by Pin1 in HEK293T cells as in neurons or that the results will be inconclusive. We have found, previously, that HEK cell recordings are not ideal for experiments examining activity dependent regulation of Kv4.2; in this case requiring expression of Kv4.2, DPP6, p38/ERK and Pin1.

DDP6 and Kv4.2 properties: There is one figure (Fig 4) where the association of DPP6 with Kv4.2 is shown to be reduced if the p38-Pin1 pathway is interrupted or prevented in tests with the Kv4.2TA knockin mutant mouse line. This is the basis for later interpretations of physiological tests using a solid battery of p38 and Pin1 blockers in Fig. 5 (how fast are these effects?). However, it has been shown that the effects of ERK-mediated phosphorylation on Kv4 channel properties is actually dependent on KChIPs that represent another important component of the Kv4 complex (Schrader et al., 2006). This needs to be considered and the results interpreted in light of this if KChIPs were not coexpressed in all the protein biochemical tests (or do these cells endogenously express KChIPs?). Could the effects of p38-mediated phosphorylation also be KChIP-dependent?

Response: The slices are incubated in the recovery solution which contains the blockers for an hour before recording so this would be the fastest we have tested. The reviewer wondered about the role of KChIPs. To our knowledge KChIPs are not expressed in HEK cells and were not coexpressed in our biochemical experiments performed in HEK cells but we have noted the Schrader results and possible role of KChIPs in the revised Discussion.

Secondly, there are no actual stimuli applied to invoke the changes in spike firing or Kv4 properties presented in Fig 5. If this supports the presence of a basal level of Pin1 activity this should be discussed. But I would have expected some number of these tests to have been conducted on slices from PTZ or KA treated hippocampus to be in line with the rest of the protein biochemical tests, with the advantage of applying some of the blockers they have available. In a similar manner, while all the final tests on Kv4 current are valuable and central to the interpretations applied

throughout all of the tests are essentially on preventing the p38-Pin1-DPP6 cascade from being active. While the interpretations of a higher basal level of DPP6 association with the Kv4.2 population is consistent with the data, the study needs to provide a straightforward test to activate this pathway to confirm the proposed effects on Kv4 current as presented in Fig 7. The results shown for AMPA in Fig 4b would appear to provide a means to do this as could PTZ or KA pretreatment as in the rest of the study.

Response: We thank the reviewer for the suggestion. We didn't see a change in the basal level of DPP6-Kv4.2 association in Kv4.2TA mice (Fig. 4e) However, we saw increased A-type current in slice recordings in Kv4.2TA mice (5j, 5k), as Reviewer 3 also mentioned. We hypothesized that this could result from the activation of p38 during slicing and recovery. We tested this by comparing p38 phosphorylation after slicing and 15min recovery at 32□ with un-sliced brain tissue. The results showed that slicing and recovery largely activates p38, and Kv4.2 phosphorylation at T607 is also increased after slicing and recovery. These data suggested that slicing and recovery process before recording has already activated p38-Pin1-Kv4.2 pathway, and the data is consistent with our hypothesis. The additional treatment with PTZ or KA may not yield a significant change since p38 is largely activated during the slicing and recovery. We added this finding in the Discussion and included the data in a Supplemental figure (Supplemental figure 9).

Since this lab has made use of DPP6 KO animals in the past I was surprised not to see some direct tests on Kv4 current from these animals to compare to channels that have lost DPP6 in the manner proposed here. In the study of LinLin (2018) from this lab it is stated that DPP6 KO mice have altered water maze testing results. How do those compare to the dynamic loss of DPP6 and water maze results here? Use of shRNA to knock down DPP6 for Kv4 current recordings in hippocampal cultures?

Response: The reviewer raised a question about the phenotypes of dynamic loss of DPP6 vs permanent loss of DPP6. This is an interesting question but there are significant differnces. In our previous paper, we showed decrease of dendritic Kv4.2 current and enhanced dendritic excitability in DPP6 KO CA1 pyramidal neurons while whole-cell recordings from the soma did not show changes in firing[1]. The Kv4.2-DPP6 complex is relatively stable in Kv4.2TA mice compared to WT (Fig. 4e), and thus we saw increase of Kv4.2 current and decreased excitability in somatic recordings in Kv4.2TA CA1 pyramidal neurons (Fig. 5). In Morris water maze, DPP6 KO showed impaired initial learning[2] while Kv4.2TA mice showed normal initial learning but improved reversal learning (Fig. 6). Kv4.2 current and neuronal excitability are direct readout of Kv4.2 channel complex, while behavioral level readout integrates related downstream effects. DPP6 modulates Kv4.2 but also functions independent of Kv4.2[3]. Therefore, the behavioral phenotypes may result from DPP6's function that is independent of Kv4.2. shRNA knockdown of DPP6 will have the same problems. We do plan in a follow up paper to investigate dendritic excitabililty and plasticity in Kv4.2TA mice in an attempt to uncover the mechanisms behind our observed enhancement of cognitive flexability.

I would also like to see the effects of Pin1 blockers on Kv4 current recorded in wt vs Kv4.2TA in Fig. 5h. In the Discussion a section should be added to address the recorded effects on Kv4 activation and inactivation properties as these are affected by DPP6 expression but did not apparently change between wt and Kv4.2TA cells (Suppl Table).

Response: We performed additional recordings using the Pin1 inhibitor, PiB, and this is now in Figure 5. PiB treatment increases Kv4 current in WT mice (Fig. 5g, 5h) and does not alter Kv4.2 current in Kv4.2TA mice (Supplementary Fig. 7). Comparison of Kv4 activation and inactivation properties between WT and Kv4.2TA cells is included in the Discussion.

Minor Comments:
- While the protein biochemical tests in the majority of figures are backed up by quantification Fig 1 stands out in not having that analysis on blots that are not as convincing as later tests.

Response: We performed additional experiments for Fig. 1c, 1e and 1f. The quantification data are now included in these figures.

- The manuscript suffers from a difficult writing style that often fails to introduce new terms or molecular paths sufficiently to allow readers to understand what is being stated. There is sometimes a mismatch in the conclusions being drawn between the main text and the Figure Legends, which themselves are not adequate to describe what is being shown. This is particularly the case in Fig 1 where the text correctly refers to a dramatic reduction in band intensity and the legend used the term "abolished" throughout. Fig 1 legend one also stands out as one of the weakest in terms of labeling and information available in the legend itself.

Response: Sorry for the confusion. We introduced new terms and added more background where they are appropriate. We changed the term "abolished" into "significantly reduced". We revised Fig. 1 legend to improve the labeling.

- Introduction - A large portion of the Introduction deals with the published effects of Kv4.2 KOs on LTD and LTP but these were ultimately not tested in the current study. Would recommend focusing on the known roles of Kv4.2 KOs on firing properties [[or other aspects of Kv4 function]] actually tested here.

Response: Thank you for the suggestion. We deleted the introduction on LTD and LTP.

- Figure 2 - The legend states that PTZ seizures do NOT induce phosphorylation of pT602 and yet the bar plot gives it a symbol of significance (Fig. 2c).

Response: The PTZ seizures do induce a small but significant increase of pT602 phosphorylation in hippocampus. But all the other tests that we performed (KA and novel enriched environment exposure) do not induce phosphorylation of pT602. We revised the description of this result to make it clear.

- Fig 3c - no Y axis label is present

Response: We added Y axis label in Fig 3c.

- Figure 5 - no indication is made in the legend for Fig. 5g as to what is being compared in the two representative traces. I assume it is wt and Kv4.2TA but it is not indicated.

Response: We added WT and Kv4.2TA labeling in Fig. 5g (now 5j). We also added it to Fig. 5j (now 5m).

Kim YS, Hong KS, Seong YS, Park JB, Kuroda S, Kishi K, Kaibuchi K, Takai Y (1994) Phosphorylation and activation of mitogen-activated protein kinase by kainic acid-induced seizure in rat hippocampus. Biochem Biophys Res Commun 202:1163–1168.

Schrader LA, Birnbaum SG, Nadin BM, Ren Y, Bui D, Anderson AE, David Sweatt J (2006) ERK/MAPK regulates the Kv4.2 potassium channel by direct phosphorylation of the pore-forming subunit. American Journal of Physiology-Cell Physiology 290:C852–C861

Zhu X, Dong J, Han B, Huang R, Zhang A, Xia Z, Chang H, Chao J, Yao H (2017) Neuronal Nitric Oxide Synthase Contributes to PTZ Kindling Epilepsy-Induced Hippocampal Endoplasmic Reticulum Stress and Oxidative Damage. Front Cell Neurosci 11:377.

Reviewer #2 (Remarks to the Author):

Hu et al. present a novel potential mechanism by which the isomerase Pin1 may regulate the assembly of Kv4.2 with DPP6 and thereby the activity of Kv4.2 channels which impacts neuronal excitability and behaviour. Starting with the observation of Pin1 binding to Kv4.2 in a heterologous screening experiment, they further characterized the putative Pin1 binding motif(s) and found increased phosphorylation of Kv4.2 at position T607 and reduced interaction with DPP6 in paradigms of neuronal stimulation. p38 MAP kinase increased phosphorylation of T607 in a model peptide which enhanced binding to Pin1. The authors finally generated a Kv4.2(T607) mouse model to confirm these findings and found an effect of this mutation on reversal learning interpreted in line with their proposed mechanism.

Despite the employment of a multitude of techniques, the basic hypothesis and key findings are based on heterologous/recombinant experiments with poor quality antibody readout. In fact, IP-verified antibodies, Kv4.2 knockout mouse control and mass spectrometry are all available to perform sensitive, quantitative and stringently controlled state of the art biochemical experiments.
Furthermore, important data and experimental details are missing (in particular on mass spectrometry, generation/verification of antibody tools and recombinant constructs).
I have substantial doubts about the validity of the basic hypothesis , interpretation and quality of the data and therefore can not recommend publication of this work.

Response: The antibodies used in our manuscript are verified and they are not poor quality antibodies. We have been using the Kv4.2 and DPP6 antibodies for many years and they are verified for western blot and IP by Kv4.2 and DPP6 knockout mice. The pT602 and pT607 antibodies were verified by point mutants (Supplementary Fig. 2, now Supplementary Fig. 3a,b) and Kv4.2TA mice (Supplementary Fig. 5c, now Supplementary Fig. 6c), and we added a phosphatase-dependent recognition experiment in the revised manuscript (Supplementary Fig. 3c). We used Kv4.2 knockout mice as control whenever approporate in our experiments, such as Fig. 1a (previous Fig. 1b), Fig. 2d, Fig. 4a, b, e (previous Fig. 4a, d). And the results are repeated and quantified. We have included the “Expression constructs”, “Antibodies” and “Tandem Affinity Purification-Mass spectrometry (TAP-MS) assay” in the Methods section. In this revised manuscript, we now included the TAP-MS data in Supplementary Fig. 1 and western blot and quantification in Methods section.

To mention the main concerns / shortcomings:

- AP-MS analyses of native Kv4.2 have been carried out before with at least reasonable quality (e.g. Marionneau et al.) which offered a quite comprehensive

view on the Kv4.2 interactome. This work should be acknowledged and discussed. It represents an experimental standard to be met.

Response: We thank the reviewer for the suggestion. We have cited Marionneau's work and compared it with our TAP-MS result in the revised manuscript.

- The authors fail to convincingly show that Pin1 binds to endogeneous Kv4.2 under native conditions (Fig. 1b shows a low quality Westernblot with a weak Pin1 signal that is also present in the KO control eluate). Modern mass spectrometry is more sensitive and quantitative than Westernblot and should be used as a readout for this experiment.

Response: We identified Pin1 as a binding partner using tandem affinity purification- mass spec (TAP-MS) technique that was recently developed [4] (Supplementary Fig. 1), and then we confirmed the association of Pin1 and Kv4.2 using co-immunoprecipitation (Fig. 1a). This is a standard work flow for protein-protein interaction identification. Modern mass spectrometry may be more sensitive, but as the reviewer also pointed out in the latter comments that "stable binding of an isomerase to a protein is not expected if the protein is a substrate", it will be challenging to identify Pin1 after two-step tendem affinity purification. Since we already know Pin1 is a potential binding protein for Kv4.2 based on our work of point mutations (Fig. 1c, 1f) and phospho-peptide pulldown (Fig. 1e), we would rather use a binding assay than a mass spec screen. In the revised manuscript, we included a new data that showed Pin1 colocalized with Kv4.2 in cultured hippocampal neurons (Fig. 1b).

- The authors mention MS results from initial pulldowns from HEK cells and neuronal cultures but do not present any of that data. Furthermore, these experiments lack adequate controls and are - by their nature (overexpression, culture systems) – prone to artifacts. Explicitly, IgG or, worse, „empty" beads are insufficient controls for any affinity-based biochemical experiments.

Response: Thank the reviewer for the suggestion. We added the TAP-MS data from cultured hippocampal neurons (Supplementary Fig. 1b) and HEK293T cells (Supplementary Fig. 1c, 1d). In our MS work, we used the recently developed technique—TAP-MS, which took the advantage of two-step tendem affinity purification so that the isolated protein complex is much purer than a one-step antibody pulldown [4]. We used Lenti-virus expressing TAP-Kv4.2-IRES-GFP for the Kv4.2 protein complex purification and control Lenti-virus expressing GFP as the purification control. The unconjugated beads were used as a negative control for Pin1 binding in Fig. 1e, but we also included the non-phospho-peptides as a control for phospho-peptides binding (Fig. 1e). We never only use "empty" beads as negative controls in our manuscript. For all the Kv4.2 co-IPs from mouse brains, we used Kv4.2 knockout mice as control as shown in Fig. 1a (previous Fig. 1b) and Fig. 4a, b, e (previous Fig. 4a, d).

- No compelling evidence is presented to verify the specificity of the polyclonal phospho-specific antibodies that were used throughout the paper for phosphorylated versus non-phosphorylated Kv4.2 wt or mutant protein. The experiment in S2 b shows Westernblot analysis of heterologously expressed Kv4.2 wt and point mutants. The phosphorylation status of these Kv4.2 proteins is not known, so it is not clear whether these antibodies are strictly phospho- or sequence-specific (or a mixture of both).

Response: To further verify the specificity of the phospho-specific Kv4.2 antibodies, we used Lambda protein phosphatase to dephosphorylate WT Kv4.2 protein before western blot. Both the anti-pT602 and T607 antibodies did not detect WT Kv4.2 after Lambda protein phosphatase treatment, suggesting these antibodies are strictly phospho-specific. This data is now included in the supplementary Fig. 3c.

- Mass spectrometric identification of Kv4.2 phosphorylation sites should be at least confirmed with endogeneous Kv4.2 protein, and the respective pairs of annotated MS/MS spectra (i.e. of the peptides in phosphorylated and non-phosphorylated state) must be shown. Information on the search database, search algorithm and search parameters must be given.

Response: In order to confirm all the Kv4.2 phosphorylation sites (total 12 sites) that we found in MS assay endogeneously, phospho-specific antibodies need to be generated and verified. Since this result is incomplete, we decided to remove it in the revised manuscript. This removal does not affect our conclusions.

- Throughout the manuscript, the significance of Westernblot signals is heavily overrated. Density-based quantification of Westernblot signals is not accurate enough to reliably quantify the relatively small changes claimed. No information is given on how Westernblots were actually performed, recorded and quantitatively evaluated.

Response: We disagree with the chararacterization of our western blot results. The literature is full of even smaller changes pubished in the highest impact journals. We added the requested western blot and quantification information in the Methods section.

- The effects of the Kv4.2 phosphosite mutations on Pin1-association (Fig. 1f) and DPP6 binding (Fig. 4a, b, d) seem to be much smaller than the difference in binding of the respective phosphopeptides to Pin1 (Fig. 1e). Apparently, binding is determined not only by phosphorylation but also by the primary sequence and the structural context. This has to be taken into account when interpreting the results from the Kv4.2(T607A) knockin model.

Response: As noted in the original submission, the reviewer is correct that sequence and structural context affect Pin1 binding. While the mutation was made to block phosphorylation, this could also lead to structural changes affecting Pin1 binding. None of this affects our conclusions regarding Pin1 regulation of Kv4.2's role in regulating reversal learning but we have added this possibility in the revised Discussion.

- As a general remark, stable binding of an isomerase to a protein is not expected if the protein is a substrate. The same applies to protein kinases. It should be verified by structural analysis that (phosphorylated) Kv4.2 is a real substrate of Pin1 (i.e. undergoes the expected structural change catalyzed by Pin1).

Response: Thank the reviewer for the suggestion. NMR and X-ray crystallography would be ideally used to determine directly the cis/trans state of prolines. We looked into companies to perform the NMR, but unfortunately, the service was cost-prohibitive. Alternatively, partial proteolysis by subtilisin, a protease particularly sensitive to substrate conformation, has been used to indicated structural rearrangement in certain proteins by Pin1[5-7]. We now performed the partial proteolysis assay by subtilisin on Kv4.2 to verify whether the prolyl-isomerase activity of Pin1 induces conformational changes in Kv4.2. We report in the revised manuscript that GST-Pin1 WT impaired Kv4.2 proteolysis by subtilisin while the isomerase-deficient mutant GST-Pin1C113S does not (Fig. 1k). Furthermore, when Kv4.2 was treated with Lambda protein phosphatase before proteolysis, GST-Pin1 lost the ability to impact Kv4.2 proteolysis (Fig. 1k). These data indicated that Pin1 elicits structural rearrangements in Kv4.2 in phosphorylation-dependent manner.

Reviewer #3 (Remarks to the Author):

KV4.2 is the primary A-type K channel and regulates neuronal excitability, which is implicated in synaptic plasticity, behaviors, and disorders. Hu et al. showed here that Kv4.2 interacts with Pin1 through the phosphorylation of KV4.2 by ERK p38. The KV4.2 phosphorylation level is altered in mouse with the enriched environment or seizure-inducing drugs. Furthermore, the KV4.2 phosphorylation regulates KV4.2 and DPP6 interaction. Finally, the knockin mice carrying the non-phospho mimic mutation in KV4.2 showed altered excitability and behaviors. From these results, the authors conclude the role of the macromolecular KV4.2 complex in regulating brain function. Overall, a significant amount of results were shown, and the proposed regulation of the KV4.2 complex is novel, but its conclusion is not fully supported.

Major
1, Protein complex
The authors proposed that Pin1 interaction with KV4.2 dissociates DPP6 from KV4.2 based on their data showing each interaction. However, dynamic regulation of the tripartite complex of the Pin1/KV4.2/DPP6 in vivo has not been shown. In another word, does Pin1 interaction dissociates DPP6 from KV4.2 or does Pin1 interact better with KV4.2 alone? More importantly, it remains unclear how the cytoplasmic Pin1 affects DPP6 interaction with KV4.2 through their transmembrane domains. Since this is the main point, this should be clearly shown.

Response: We appreciate the reviewer's suggestions. Our data indicated that Pin1 binding to Kv4.2 increases following exposure to novel environment or seizure, however, Pin1 isomerase activity is required for dissociation of DPP6 from Kv4.2 (Fig. 4b, c). Pin1 interaction with Kv4.2 is a necessary step for Pin1 to change the configuration of Kv4.2. Kv4.2-DPP6 form relative stable tetrameric complex[8] while Pin1 binding to Kv4.2 at T607 is relatively transient in response to neuronal activity. We show Pin1 binding to Kv4.2 is dependent on phosphorylation of Kv4.2 at T607 (and T602) and not dependent on DPP6. We performed a coIP experiment that showed DPP6 addition did not alter Kv4.2 and Pin1 binding. This data is attached as a figure for the reviewer.
The reviewer also raised the question about the mechanism of Kv4.2-DPP6 dissociation in response to Kv4.2 configuration change. We appreciate the reviewer's suggestion and enthusiasm. This is an interesting question but how the Kv4.2 cis/trans structural switch leads to dissociation of the Kv4.2-DPP6 complex is beyond the scope of this report. Our data shows that activity-dependent Pin1 binding and isomerization of Kv4.2 leads to dissociation of the Kv4.2-DPP6 complex, regulating neuronal excitability and cognitive function. We have discussed this in the revised manuscript.

2, Characterization of novel KV4.2 knockin mice.

The authors produced novel KV4.2 TA knockin mice and provided several phenotypes. Notably, changes in the firing frequency strongly indicate the significance of this phosphorylation sites. However, the data requires some critical control. Fig 5a.b, the authors concluded that the reduction in the firing frequency by Pin1 inhibitor and rescue by AmmTX3 suggests both on a similar pathway. However, AmmTX3 depolarizes more independent from the Pin1 pathway. To clarify this, it is important to provide AMMTx3 alone in Fig 5b.

Response: We thank the reviewer for the suggestion and have performed the neuronal excitability experiment using AMMTx3 alone. We show that AmmTX3 does not enhance exctitability any further than AmmTX3+PiB when applied alone (Figure 5a.b).

3, Connection between the biochemical and physiological phenotypes. The authors showed that DPP6 and KV4.2 form a complex similarly in WT and KV4.2 TA mice before the drug treatment (Fig 4d). On the other hand, their firing frequency is different (Fig 5a). Does this mean that repetitive stimulation causes DPP6 dissociation from KV4.2? The authors need to explain this further, and this may require additional experiments.

Response: We don't think repetitive stimulation causes DPP6 dissociation from Kv4.2 since the stimulation just takes a few seconds and such dissociation would result in excitability changes which would have been indentified by us or others in the field long ago.
We now think this difference results from different procedures for biochemistry and recording experiments. As noted in response to Reviewer 1 above, we tested this by comparing p38 phosphorylation after slicing and 15min recovery at 32□ with un-sliced brain tissue. The results showed that slicing and recovery largely activates p38, and Kv4.2 phosphorylation at T607 is also increased after slicing and recovery. These data suggested that slicing and recovery process before recording has already activated p38-Pin1-Kv4.2 pathway, and the data is consistent with our hypothesis. We added this finding in the Discussion and included the data in a Supplemental figure (Supplemental figure 9).

Minor
Table of MS analysis of the KV4.2 complex should be shown. Especially, because PIN1 is identified from HEK proteomics, but not neuron proteomics, and because the Pin1 Co-IP on Fig 1b is modest.

Response: Thank the reviewer for the suggestion. We added the TAP-MS data in Supplementary Fig. 1 in the revised manuscript. We also compared our TAP-MS data with a MS study of Kv4.2 complex from brain that mainly identified known Kv4.2 binding proteins [10]. We think Pin1-Kv4.2 binding is induced by neuronal activity and not stable so that it is challenging to identify Pin1 in endogenously.

Requires control, phosphatase-treated samples, to show phosphorylation-dependent interaction.

Response: As suggested by the reviewer, we performed GST-Pin1 pulldown of HEK-293T expressed Kv4.2 with and without Lambda protein phosphatase treatment. The result showed that Lambda protein phosphatase treatment significantly reduced Kv4.2 binding to Pin1. We included this data in Supplementary Fig. 2a in the revised manuscript.

Knockin mice. The knockin mice at T607 showed altered phosphorylation at T602 and altered IP efficiency. Please discuss.

Response: The Kv4.2TA mice seems reduced the pT602 detection but we didn't observe a consistent alteration of Kv4.2 IP efficiency (Fig. 4e and supplementary Fig. 6c). The reduced pT602 could result from the decrease of pT602 antibody recognition or pT607's impact on pT602. We added it in Supplementary Fig. 6 in the revised manuscript.

Fig 4. The title indicates that the main point is phosphorylation-dependent interaction with the other proteins. It is also required to show a blot with the pT607 antibody.

Response: The pT607 blot of Kv4.2TA mice was shown in supplementary Fig. 6c. The main point of Fig. 4 is that p38-Pin1-Kv4.2 pathway regulates the composition of the Kv4.2-DPP6 complex. We now revised the Fig. 4 title to "P38-Pin1-Kv4.2 pathway regulates the composition of the Kv4.2-DPP6 complex."

Discussion. The effect size in activity-dependent interaction is relatively modest. It should be discussed why the effect size is modest despite a lovely model on Fig 7.

Response: A certain activity or stimulation can only trigger a subset of neurons. We suggest that the modest effect is because the p38-Pin1-Kv4.2 mechanism only occurs in a subset of neurons that are activated by neuronal activity or other stimulations, while the biochemistry experiments collected all the neurons that included the un-activated neurons.

Statistics. Was the data normality tested?

Response: We did the normality test for all the data from the recordings.

Reviewers' comments:

Reviewer #1 (Remarks to the Author):

Notes to Authors:
The authors have directly addressed my major concerns experimentally, or through Discussion points. My issues with the original manuscript centered primarily around 4 points:

1) Whether much of the data could be accounted for by ERK-mediated phosphorylation compared to p38. The experimental tests cited in Fig. 3g, 4a and 5g,h convince me that a large portion of the responses could be attributed to the actions of p38. One proviso here is that they state that the data in Fig. 3g shows that a MEK inhibitor "does not block" PTZ-induced phosphorylation". In viewing this plot I would like them to be clear in concluding instead that "PTZ-induced phosphorylation is only partly reduced" by this MEK inhibitor since the degree of significance drop from two stars to one.

2) The potential role of KChiPs in mediating some of the effects here. The authors largely deflect this request but at least consider the Schrader results in the Discussion.

3) The use of DPP6 KO animals. A detailed response is given that makes it clear they are aware of differences between experimental paradigms which could reflect off target effects or even compensation that I am willing to accept.

4) Apply a Pin1 blocker to determine its affects on Kv4 current. Experimental evidence of an increase in Kv4.2 current is now provided in Fig. 5.

An area that was not addressed but was stated to be was a suggestion to remove the off-relevant topic of Kv4 to LTP and LTD originally described in the Introduction. The authors indicate they had removed that but it is still in my copy. While it is interesting data it has little to do with this study except perhaps as a Discussion point on excitability such that removing it would make the introduction more consistent with the current study

Reviewer #2 (Remarks to the Author):

Hu et al. added substantial data and revised the manuscript to address several of my concerns. However, they still did not provide compelling evidence for biochemical interaction of endogenous Pin1 and Kv4.2 proteins in neurons (see below). Since this is fundamental to the chain of logic and the conclusions drawn from subsequent experiments, I can not recommend publication of this work.

To the author responses:

>Response: We thank the reviewer for the suggestion. We have cited Marionneau's work and compared it with our TAP-MS result in the revised manuscript.

Concern resolved.

>Response: We identified Pin1 as a binding partner using tandem affinity purification- mass spec (TAP-MS) technique that was recently developed 4 (Supplementary Fig. 1), and then we confirmed the association of Pin1 and Kv4.2 using co-immunoprecipitation (Fig. 1a). This is a standard work flow for protein-protein interaction identification. Modern mass spectrometry may be more sensitive, but as the reviewer also pointed out in the latter comments that "stable binding of an isomerase to a protein is not expected if the protein is a substrate", it will be challenging to identify Pin1 after two-step tendem affinity purification. Since we already know Pin1 is a potential binding protein for Kv4.2 based on our work of point mutations (Fig. 1c, 1f) and phospho-peptide pulldown (Fig. 1e), we would rather

use a binding assay than a mass spec screen. In the revised manuscript, we included a new data that showed Pin1 colocalized with Kv4.2 in cultured hippocampal neurons (Fig. 1b).

The work flow the authors used and claim „a standard" is certainly not state of the art for reliable de-novo identification of native protein interactions (15 years after the introduction of high-resolution mass spectrometry!). Heterologous screens combined with western blot readout from native pulldowns have in fact strongly contributed to the large number of false-positive (in particular membrane) protein interactions in the literature and public databases.
As pointed out in my previous comment, the Pin1 signals in the co-immunoprecipitation experiment in Fig. 1a (and the original blot scan provided) have very low intensities over background and merely differ between WT and Kv4.2 knockout control (by a rough factor of 2 by densitometric evaluation, far below an acceptable ratio for specific co-purification). Recombinant binding assays (Fig. 1c, e, f) and the newly added co-localization data (Fig. 1b) do not substitute for confirmation by stringently controlled quantitative IP-MS analysis from native neurons/brain.

>Response: Thank the reviewer for the suggestion. We added the TAP-MS data from cultured hippocampal neurons (Supplementary Fig. 1b) and HEK293T cells (Supplementary Fig. 1c, 1d). In our MS work, we used the recently developed technique—TAP-MS, which took the advantage of two-step tendem affinity purification so that the isolated protein complex is much purer than a one-step antibody pulldown 4. We used Lenti-virus expressing TAP-Kv4.2-IRES- GFP for the Kv4.2 protein complex purification and control Lenti-virus expressing GFP as the purification control. The unconjugated beads were used as a negative control for Pin1 binding in Fig. 1e, but we also included the non-phospho-peptides as a control for phospho-peptides binding (Fig. 1e). We never only use "empty" beads as negative controls in our manuscript. For all the Kv4.2 co-IPs from mouse brains, we used Kv4.2 knockout mice as control as shown in Fig. 1a (previous Fig. 1b) and Fig. 4a, b, e (previous Fig. 4a, d).

The TAP-MS data provided in the new Supplementary Fig. 1 does demonstrate the mass spectrometric identification of Pin1 but lacks determination of specificity (number of other proteins identified, quantification in Kv4.2 versus control). No information is given on how/which gel bands were selected and how MS analysis was performed.

>Response: To further verify the specificity of the phospho-specific Kv4.2 antibodies, we used Lambda protein phosphatase to dephosphorylate WT Kv4.2 protein before western blot. Both the anti-pT602 and T607 antibodies did not detect WT Kv4.2 after Lambda protein phosphatase treatment, suggesting these antibodies are strictly phospho-specific. This data is now included in the supplementary Fig. 3c.

The data provided in Fig. 3c is convincing and answers my concern.

>Response: In order to confirm all the Kv4.2 phosphorylation sites (total 12 sites) that we found in MS assay endogeneously, phospho-specific antibodies need to be generated and verified. Since this result is incomplete, we decided to remove it in the revised manuscript. This removal does not affect our conclusions.

Agreed.

>Response: We disagree with the chararacterization of our western blot results. The literature is full of even smaller changes pubished in the highest impact journals. We added the requested western blot and quantification information in the Methods section.

I appreciate the addition of this important information to Methods although this does not change my scepticism concerning the western blot quantification results. I am aware of many poor (i.e. non-convincing) western blot results in the literature (with the negative impacts outlined above) but these should not be taken as a reference, in particular with quantitative mass spectrometry being available

as a superior readout.

>Response: As noted in the original submission, the reviewer is correct that sequence and structural context affect Pin1 binding. While the mutation was made to block phosphorylation, this could also lead to structural changes affecting Pin1 binding. None of this affects our conclusions regarding Pin1 regulation of Kv4.2's role in regulating reversal learning but we have added this possibility in the revised Discussion.

Concern resolved.

> Response: Thank the reviewer for the suggestion. NMR and X-ray crystallography would be ideally used to determine directly the cis/trans state of prolines. We looked into companies to perform the NMR, but unfortunately, the service was cost-prohibitive. Alternatively, partial proteolysis by subtilisin, a protease particularly sensitive to substrate conformation, has been used to indicated structural rearrangement in certain proteins by Pin15-7. We now performed the partial proteolysis assay by subtilisin on Kv4.2 to verify whether the prolyl-isomerase activity of Pin1 induces conformational changes in Kv4.2. We report in the revised manuscript that GST- Pin1 WT impaired Kv4.2 proteolysis by subtilisin while the isomerase-deficient mutant GST- Pin1C113S does not (Fig. 1k). Furthermore, when Kv4.2 was treated with Lambda protein phosphatase before proteolysis, GST-Pin1 lost the ability to impact Kv4.2 proteolysis (Fig. 1k). These data indicated that Pin1 elicits structural rearrangements in Kv4.2 in phosphorylation- dependent manner.

The added data (Fig. 1j, k) is not entirely convincing and raises some questions. Why was the 47 kDa fragment used to quantify proteolysis? It seems to be present (but why?) in the lysate before subtilisin addition (Fig. 1j) and further digested with higher concentrations of subtilisin (Fig. 1j). Appearance of the 33 kDa fragment should be a better measure but seems to be unchanged in Fig. 1k.

Reviewer #3 (Remarks to the Author):

This is a revised manuscript I reviewed before. The authors responded to several points adequately. However, they need further clarification.

Major
1, Protein complex
Upon reading the response, I still think that the author did not provide evidence to support their interaction model in Figure 7. The authors failed to show dynamic regulation of the tripartite complex of the Pin1/KV4.2/DPP6 in vivo. PTZ injection dissociates DPP6 from Kv4.2 (Fig 4b). Under this condition, did PTZ injection increase co-IP of KV4.2 and Pin1 (Refer the method in Fig 1a) specifically in WT, but not in KV4.2 T607A knock-in mice? Notably, GST-pull down (Fig 4d) does not support their in vivo interaction.

It is questionable to value "a coIP experiment that showed DPP6 addition did not alter Kv4.2 and Pin1 binding" due to micelles formed by detergents. DPP6 is a transmembrane protein, and the addition of DPP6 embedded in detergent micelles to another transmembrane protein complex in other micelles should not interact due to micelles.

I emphasize that I agree with significance of activity-dependent isomerization based on pharmacological data and KV4.2 T607 phosphorylation site based on knockin mice. But the proposed model in Figure 7 is not fully supported in vivo.
I think if the model Figure 7 is not fully supported, this should not be shown to avoid biasing readers.

2, Characterization of novel KV4.2 knockin mice.

The authors responded adequately to provide new control data.

3, Connection between the biochemical and physiological phenotypes.
Now the authors provided new results showing hyper-phosphorylation of p38 and T607 upon slice procedures before recording (Supplementary Figure 9). Supplementary Figure 9 is very critical for interpreting their results. Therefore, representative blots and a summary of quantification must be presented as a part of Figure 5.

Minor
All points except one were responded adequately.

Regarding the Minor point #1, it is more informative to show an entire, but not selected, list of proteins hit by MS analysis of the KV4.2 complex (Supplementary Figure 1). Readers need to know the robustness of Pin1 as interaction by evaluating Pin1 as the highest hit or one of many proteins.

Reviewers' comments:

Reviewer #1 (Remarks to the Author):

Notes to Authors:

The authors have directly addressed my major concerns experimentally, or through Discussion points. My issues with the original manuscript centered primarily around 4 points:

1) Whether much of the data could be accounted for by ERK-mediated phosphorylation compared to p38. The experimental tests cited in Fig. 3g, 4a and 5g,h convince me that a large portion of the responses could be attributed to the actions of p38. One proviso here is that they state that the data in Fig. 3g shows that a MEK inhibitor "does not block" PTZ-induced phosphorylation". In viewing this plot I would like them to be clear in concluding instead that "PTZ-induced phosphorylation is only partly reduced" by this MEK inhibitor since the degree of significance drop from two stars to one.

Response: We made the revisions as suggested.

2) The potential role of KChiPs in mediating some of the effects here. The authors largely deflect this request but at least consider the Schrader results in the Discussion.

Response: Thank you.

3) The use of DPP6 KO animals. A detailed response is given that makes it clear they are aware of differences between experimental paradigms which could reflect off target effects or even compensation that I am willing to accept.

Response: Thank you.

4) Apply a Pin1 blocker to determine its affects on Kv4 current. Experimental evidence of an increase in Kv4.2 current is now provided in Fig. 5.

Response: Thank you.

An area that was not addressed but was stated to be was a suggestion to remove the off-relevant topic of Kv4 to LTP and LTD originally described in the Introduction. The authors indicate they had removed that but it is still in my copy. While it is interesting data it has little to do with this study except perhaps as a Discussion point on excitability such that removing it would make the introduction more consistent with the current study

Response: We deleted one sentence in the last revision, and now we deleted all the LTP/LTD in the introduction.

Reviewer #2 (Remarks to the Author):

Hu et al. added substantial data and revised the manuscript to address several of my concerns. However, they still did not provide compelling evidence for biochemical interaction of endogenous Pin1 and Kv4.2 proteins in neurons (see below). Since this is fundamental to the chain of logic and the conclusions drawn from subsequent experiments, I can not recommend publication of this work.

To the author responses:

>Response: We thank the reviewer for the suggestion. We have cited Marionneau's work and compared it with our TAP-MS result in the revised manuscript.

Concern resolved.

Response: Thank you.

>Response: We identified Pin1 as a binding partner using tandem affinity purification- mass spec (TAP-MS) technique that was recently developed 4 (Supplementary Fig. 1), and then we confirmed the association of Pin1 and Kv4.2 using co-immunoprecipitation (Fig. 1a). This is a standard work flow for protein-protein interaction identification. Modern mass spectrometry may be more sensitive, but as the reviewer also pointed out in the latter comments that "stable binding of an isomerase to a protein is not expected if the protein is a substrate", it will be challenging to identify Pin1 after two-step tendem affinity purification. Since we already know Pin1 is a potential binding protein for Kv4.2 based on our work of point mutations (Fig. 1c, 1f) and phospho-peptide pulldown (Fig. 1e), we would rather use a binding assay than a mass spec screen. In the revised manuscript, we included a new data that showed Pin1 colocalized with Kv4.2 in cultured hippocampal neurons (Fig. 1b).

The work flow the authors used and claim „a standard" is certainly not state of the art for reliable de-novo identification of native protein interactions (15 years after the introduction of high-resolution mass spectrometry!). Heterologous screens combined with western blot readout from native pulldowns have in fact strongly contributed to the large number of false-positive (in particular membrane) protein interactions in the literature and public databases.

As pointed out in my previous comment, the Pin1 signals in the co-immunoprecipitation experiment in Fig. 1a (and the original blot scan provided) have very low intensities over background and merely differ between WT and Kv4.2 knockout control (by a rough factor of 2 by densitometric evaluation, far below an acceptable ratio for specific co-purification). Recombinant binding assays (Fig. 1c, e, f) and the newly added co-localization data (Fig. 1b) do not substitute for confirmation by stringently controlled quantitative IP-MS analysis from native neurons/brain.

Response: Mass spectrometry is just a screening tool to provide clues for protein-protein interaction. Co-immunoprecipitation and colocalization experiments should still be used to verify the interaction following the mass spectrometry. We have done this with co- immunoprecipitation and colocalization experiments for native protein-protein binding and many mutations and peptide pulldown to identify the binding sites as showed in Fig. 1. We think these data are solid evidence of a Pin1 and Kv4.2 interaction. The other reason that do not believe it would be useful to perform quantitative IP-MS analysis from native neurons/brain is that, unlike stable interactions such as with auxiliary subunits (e.g. DPP6, KChIPs), Pin1 and Kv4.2 binding is transient (isomerase and substrate) and hard to capture, as Reviewer 2 also pointed out in the initial comments. Therefore, even if Pin1 shows up in the IP-MS result, it won't be high on the hit list. The third reason is that IP-MS just shows two proteins could be in the same complex, but no further information, such as their direct interaction, will be provided. So, we think this is not an appropriate way to prove Pin1 and Kv4.2 binding. Moreover, we do not have the equipment or expertise to perform these experiments in our lab.

In this revision, we optimized the brain co-IP condition, and provided better evidence of native interaction between Kv4.2 and Pin1 (Fig. 1a).

>Response: Thank the reviewer for the suggestion. We added the TAP-MS data from cultured hippocampal neurons (Supplementary Fig. 1b) and HEK293T cells (Supplementary Fig. 1c, 1d). In our MS work, we used the recently developed technique—TAP-MS, which took the advantage of two-step tendem affinity purification so that the isolated protein complex is much purer than a one-step antibody pulldown 4. We used Lenti-virus expressing TAP-Kv4.2-IRES- GFP for the Kv4.2 protein complex purification and control Lenti-virus expressing GFP as the purification control. The unconjugated beads were used as a negative control for Pin1 binding in Fig. 1e, but we also included the non-phospho-peptides as a control for phospho-peptides binding (Fig. 1e). We never only use "empty" beads as negative controls in our manuscript. For all the Kv4.2 co-IPs from mouse brains, we used Kv4.2 knockout mice as control as shown in Fig. 1a (previous Fig. 1b) and Fig. 4a, b, e (previous Fig. 4a, d).

The TAP-MS data provided in the new Supplementary Fig. 1 does demonstrate the mass spectrometric identification of Pin1 but lacks determination of specificity (number of other proteins identified, quantification in Kv4.2 versus control). No information is given on how/which gel bands were selected and how MS analysis was performed.

Response: Pin1 is just one of many proteins since Kv4.2 has numerous intercellular binding partners and Pin1-Kv4.2 binding is transient. We added two lists of proteins (high molecular weight and low molecular weight) identified by mass spec as supplementary information (Fig. S1c, d). The samples were run on 10% SDS-PAGE gel (Novex/Invitrogen). The gels that contains the protein samples were excised, separated into high molecular sample and low molecular sample, and sent to the Taplin Mass Spectrometry Facility at Harvard University for in-gel digestion using trypsin and mass spectrometric analysis. We added this information in the Methods.

>Response: To further verify the specificity of the phospho-specific Kv4.2 antibodies, we used Lambda protein phosphatase to dephosphorylate WT Kv4.2 protein before western blot. Both the anti-pT602 and T607 antibodies did not detect WT Kv4.2 after Lambda protein phosphatase treatment, suggesting these antibodies are strictly phospho-specific. This data is now included in the supplementary Fig. 3c.

The data provided in Fig. 3c is convincing and answers my concern.

Response: Thank you.

>Response: In order to confirm all the Kv4.2 phosphorylation sites (total 12 sites) that we found in MS assay endogeneously, phospho-specific antibodies need to be generated and verified. Since this result is incomplete, we decided to remove it in the revised manuscript. This removal does not affect our conclusions.

Agreed.

Response: Thank you.

>Response: We disagree with the chararacterization of our western blot results. The literature is full of even smaller changes pubished in the highest impact journals. We added the requested western blot and quantification information in the Methods section.

I appreciate the addition of this important information to Methods although this does not change my scepticism concerning the western blot quantification results. I am aware of many poor (i.e. non-convincing) western blot results in the literature (with the negative impacts outlined above) but these should not be taken as a reference, in particular with quantitative mass spectrometry being available as a superior readout.

Response: Reviewer 2 is being unreasonable in our estimation in that he/she thinks that western blot signals are not suitable for quantification (both in his/her initial comments and the current comments). We don't think we can satisfy a reviewer that doesn't acknowledge such a widely used and basic research tool as western blot.

>Response: As noted in the original submission, the reviewer is correct that sequence and structural context affect Pin1 binding. While the mutation was made to block phosphorylation, this could also lead to structural changes affecting Pin1 binding. None of this affects our conclusions regarding Pin1 regulation of Kv4.2's role in regulating reversal learning but we have added this possibility in the revised Discussion.

Concern resolved.

Response: Thanks.

> Response: Thank the reviewer for the suggestion. NMR and X-ray crystallography would be ideally used to determine directly the cis/trans state of prolines. We looked into companies to perform the NMR, but unfortunately, the service was cost-prohibitive. Alternatively, partial proteolysis by subtilisin, a protease particularly sensitive to substrate conformation, has been used to indicated structural rearrangement in certain proteins by Pin15-7. We now performed the partial proteolysis assay by subtilisin on Kv4.2 to verify whether the prolyl-isomerase activity of Pin1 induces conformational changes in Kv4.2. We report in the revised manuscript that GST- Pin1 WT impaired Kv4.2 proteolysis by subtilisin while the isomerase-deficient mutant GST- Pin1C113S does not (Fig. 1k). Furthermore, when Kv4.2 was treated with Lambda protein phosphatase before proteolysis, GST-Pin1 lost the ability to impact Kv4.2 proteolysis (Fig. 1k). These data indicated that Pin1 elicits structural rearrangements in Kv4.2 in phosphorylation- dependent manner.

The added data (Fig. 1j, k) is not entirely convincing and raises some questions. Why was the 47 kDa fragment used to quantify proteolysis? It seems to be present (but why?) in the lysate before subtilisin addition (Fig. 1j) and further digested with higher concentrations of subtilisin (Fig. 1j). Appearance of the 33 kDa fragment should be a better measure but seems to be unchanged in Fig. 1k.

Response: The purpose of this proteolysis assay is to show if Pin1 plays a role in in vitro degradation of Kv4.2 that is dependent on structure. In the literature, the proteolysis of a full-length protein slowed down when Pin1 is present. The proteolysis of Kv4.2 full-length protein seems normal, but we see that Pin1 protects a proteolysis fragment (47kD) of Kv4.2 degradation. The possible reason might be the different location that Pin1 binds and isomerizes a substrate. In the literature that assayed for subtilisin proteolysis, Pin1 binds to either N-terminal or middle of a protein. However, Pin1 binds and isomerizes Kv4.2 at its C-terminal, which might result in structural change different from N-terminal configuration change. The two bands that run at about 47kD in the lysate before subtilisin addition is non-specific and are degraded after subtilisin addition. The 47kD band in subtilisin samples is generated by subtilisin

because the amount of the 47kD fragment increased when more subtilisin was added. If the band is original, it can only be degraded and not increased. We don't know why the 47kD fragment changed but not the full length or the 33kD fragment, but as long as there is a difference in the process of proteolysis when Pin1 is present, it indicates that Kv4.2 structure is altered in the present of Pin1.

Reviewer #3 (Remarks to the Author):

This is a revised manuscript I reviewed before. The authors responded to several points adequately. However, they need further clarification.

Major

1, Protein complex

Upon reading the response, I still think that the author did not provide evidence to support their interaction model in Figure 7. The authors failed to show dynamic regulation of the tripartite complex of the Pin1/KV4.2/DPP6 in vivo. PTZ injection dissociates DPP6 from Kv4.2 (Fig 4b). Under this condition, did PTZ injection increase co-IP of KV4.2 and Pin1 (Refer the method in Fig 1a) specifically in WT, but not in KV4.2 T607A knock-in mice? Notably, GST-pull down (Fig 4d) does not support their in vivo interaction.

It is questionable to value "a coIP experiment that showed DPP6 addition did not alter Kv4.2 and Pin1 binding" due to micelles formed by detergents. DPP6 is a transmembrane protein, and the addition of DPP6 embedded in detergent micelles to another transmembrane protein complex in other micelles should not interact due to micelles.

I emphasize that I agree with significance of activity-dependent isomerization based on pharmacological data and KV4.2 T607 phosphorylation site based on knockin mice. But the proposed model in Figure 7 is not fully supported in vivo.

I think if the model Figure 7 is not fully supported, this should not be shown to avoid biasing readers.

Response: We appreciate the acknowledgment of the significance of our results and the reviewer's suggestion. We performed the co-IP of Kv4.2 and Pin1 experiment with / without PTZ in WT and Kv4.2TA mice as the reviewer suggested. The result showed that Pin1-Kv4.2 association is induced by PTZ in WT mice but abolished in Kv4.2TA mice (Fig. 4e).

2, Characterization of novel KV4.2 knockin mice.

The authors responded adequately to provide new control data.

Response: Thank you.

3, Connection between the biochemical and physiological phenotypes.

Now the authors provided new results showing hyper-phosphorylation of p38 and T607 upon slice procedures before recording (Supplementary Figure 9). Supplementary Figure 9 is very critical for interpreting their results. Therefore, representative blots and a summary of quantification must be presented as a part of Figure 5.

Response: We did the quantification of Supplementary Figure 9. The original Fig. 5 is overwhelming, and so we split it into two figures: Fig. 5 for neuronal excitability and Fig. 6 for A-current. The Supplementary Figure 9 was moved into Fig. 5 as Fig. 5g.

Minor

All points except one were responded adequately.

Regarding the Minor point #1, it is more informative to show an entire, but not selected, list of proteins hit by MS analysis of the KV4.2 complex (Supplementary Figure 1). Readers need to know the robustness of Pin1 as interaction by evaluating Pin1 as the highest hit or one of many proteins.

Response: Pin1 is just one of the proteins since Kv4.2 has many intercellular binding partners and Pin1-Kv4.2 binding is transient. We added two lists of proteins (high molecular weight and low molecular weight) identified by mass spec as supplementary information (Fig. S1c, d).

REVIEWERS' COMMENTS:

Reviewer #3 (Remarks to the Author):

This is a revised manuscript I reviewed before. The authors responded, and I commented onto each response from myself (R3) and R2. Upon responding to these minor comments, I recommend this manuscript to publish on Nature Communication.

R3.
1, Protein complex
The authors provided new evidence supporting activity dependent association of Kv4.2 and Pin1. Very good.

In the figure 7, T607 could be highlighted with different color and labeled as “T607”.

3, Connection between the biochemical and physiological phenotypes.
I recommend to provide quantification of all blots in Fig 5g, since the data will support the quality of the experiments raised by the reviewer 2 and because the results exist in raw blots (Fig 5g)

Minor
Now the list of Mass spec is shown. Because Pin1 is not a robust interactor, two sentences should be required for readers around the page 4, line 93.
#1, describing that Kv4.2 has many intercellular binding partners
#2, a reason to choose Pin1 (did author cherry-picking or also tried other proteins?)

In addition, it is better to add one paragraph in discussion.
why so many proteins are isolated? Are they all Kv4.2 interactors?

R2.
1, Response: Mass spectrometry…..
The reviewer is asking quantification. It would be helpful to clarify further the Method section “Western blot and quantification”, how to estimate the linear range of detection.

2, Response: Pin1 is just one….
Adequately responded.

3, Response: Reviewer 2 is….
I think the reviewer 2 is taking about the linearity in the quantification of WB. Because the raw blots do not have standard curves on each gel, it is helpful to update the linearity in the method section as noted in the 1.

4, Response: The purpose of,,,
It would be helpful for readers to mark each band with arrow, arrowhead and asterisk (background). Then, explain in the figure legend. How the authors responded is OK, but it was difficult to figure out which bands the authors and R2 are talking about.

REVIEWERS' COMMENTS:

Reviewer #3 (Remarks to the Author):

This is a revised manuscript I reviewed before. The authors responded, and I commented onto each response from myself (R3) and R2. Upon responding to these minor comments, I recommend this manuscript to publish on Nature Communication.

R3.

1, Protein complex

The authors provided new evidence supporting activity dependent association of Kv4.2 and Pin1. Very good.

In the figure 7, T607 could be highlighted with different color and labeled as "T607".

Response: We thank the reviewer for their comments and suggestions. We highlighted T607 in the model figure, which is Figure 8 in the revised version.

3, Connection between the biochemical and physiological phenotypes.

I recommend to provide quantification of all blots in Fig 5g, since the data will support the quality of the experiments raised by the reviewer 2 and because the results exist in raw blots (Fig 5g)

Response: We quantified p38 and ERK as suggested. P38: Ctl: 100 ± 6.23%; slicing: 93.30 ± 2.80%, $p = 0.4262$. ERK: Ctl: 100 ± 3.85%; slicing: 100.36 ± 3.31%, $p = 0.9492$. These are now included in the Results section.

Minor

Now the list of Mass spec is shown. Because Pin1 is not a robust interactor, two sentences should be required for readers around the page 4, line 93.

#1, describing that Kv4.2 has many intercellular binding partners

#2, a reason to choose Pin1 (did author cherry-picking or also tried other proteins?)

In addition, it is better to add one paragraph in discussion.

why so many proteins are isolated? Are they all Kv4.2 interactors?

Response: We added the explanation in the Results and Discussion as suggested by the reviewer.

R2.

1, Response: Mass spectrometry…..

The reviewer is asking quantification. It would be helpful to clarify further the Method section "Western blot and quantification", how to estimate the linear range of detection.

Response: We added in the Methods section that linear range of detection is determined by using serial dilutions of a representative sample, as we reported previously (Hu JH et al, Neuron. 2010;68(6):1128-42; Hu JH et al, Nature neuroscience. 2012;15(6):836-44; Park JM et al. Cell. 2013; 154(3):637-50.)

2, Response: Pin1 is just one….

Adequately responded.

3, Response: Reviewer 2 is….

I think the reviewer 2 is taking about the linearity in the quantification of WB. Because the raw blots do not have standard curves on each gel, it is helpful to update the linearity in the method section as noted in the 1.

Response: See above.

4, Response: The purpose of,,,

It would be helpful for readers to mark each band with arrow, arrowhead and asterisk (background). Then, explain in the figure legend. How the authors responded is OK, but it was difficult to figure out which bands the authors and R2 are talking about.

Response: We added an arrow, arrowhead and asterisk to the figure to make it clear.
